# Selective Laser Trabeculoplasty After Medical Treatment for Glaucoma or Ocular Hypertension

**DOI:** 10.1001/jamaophthalmol.2024.6492

**Published:** 2025-02-20

**Authors:** Evgenia Konstantakopoulou, Gus Gazzard, David Garway-Heath, Mariam Adeleke, Gareth Ambler, Victoria Vickerstaff, Catey Bunce, Neil Nathwani, Keith Barton

**Affiliations:** 1 NIHR Biomedical Research Centre at Moorfields Eye Hospital and UCL Institute of Ophthalmology, London, United Kingdom; 2 Division of Optics and Optometry, University of West Attica, Athens, Greece; 3 Department of Statistical Science, University College London, London, United Kingdom; 4 PRIMENT Clinical Trials Unit, University College London, London, United Kingdom; 5 The Research Department of Primary Care and Population Health, University College London, London, United Kingdom; 6 Marie Curie Palliative Care Research Department, UCL Division of Psychiatry, University College London, London, United Kingdom; 7 Research Data and Statistics Unit, Royal Marsden NHS Foundation Trust, London, United Kingdom; 8 London School of Hygiene & Tropical Medicine, London, United Kingdom

## Abstract

**Importance:**

Primary selective laser trabeculoplasty (SLT) is a safe primary treatment for open-angle glaucoma (OAG) and ocular hypertension (OHT). However, there is limited evidence on its use as a secondary treatment, ie, after prior use of ocular hypotensive eye drops.

**Objective:**

To evaluate outcomes following SLT after using hypotensive eye drops for at least 3 years.

**Design, Setting, and Participants:**

This is a post hoc exploratory analysis of data from a multicenter randomized clinical trial conducted within the UK National Health Service. Participants were patients with OAG or OHT who participated in the LiGHT trial. Data were analyzed from February 2021 to December 2024.

**Intervention:**

Participants were initially randomized to either primary SLT or primary hypotensive eye drops and remained on the allocated treatment pathway for 3 years. Participants using eye drops were then allowed to have secondary SLT as a treatment switch (to reduce their medication load) or as a treatment escalation (if more intense treatment was needed). Participants were treated and monitored according to a predefined protocol.

**Main Outcomes and Measures:**

The outcomes of interest were rates of incisional glaucoma surgery, medication use, and intraocular pressure.

**Results:**

In total, 633 participants entered the extension of the LiGHT trial, and 524 participants (82.8%) completed the extension (72 months). Of 320 participants receiving primary hypotensive eye drops, 112 (35.0%) received SLT: 70 participants switched to SLT, 29 participants had SLT as a treatment escalation, and 13 participants had SLT as a treatment escalation in 1 eye and as a treatment switch in the other eye. Switching to SLT was associated with a reduction in the number of medications (mean [SD], 1.38 [0.62] to 0.59 [0.92] active ingredients; mean difference, 0.79 [95% CI 0.66 to 0.93] active ingredients; *P* < .001). At 72 months, 69 eyes that switched to SLT (60.5%) needed no medical or surgical treatment, and 62 eyes receiving 1 drug before switching (83.8%) needed no medical treatment. Escalating to SLT was associated with a mean intraocular pressure reduction of 4.6 mm Hg (21.8%), and 30 eyes (62.5%) reached target intraocular pressure at 72 months without the need for surgery; 9 eyes (18.7%) needed a trabeculectomy.

**Conclusions and Relevance:**

This secondary analysis of a randomized clinical trial found that secondary SLT was associated with a reduction in the medication load for stable, medically treated eyes. For medically uncontrolled eyes, there is evidence that SLT could provide additional intraocular pressure control, but the need for trabeculectomy was not eliminated.

**Trial Registration:**

isrctn.org Identifier: ISRCTN32038223

## Key Points

**Question:**

Is secondary SLT associated with improved disease control among patients with open-angle glaucoma or ocular hypertension after the use of topical intraocular pressure–lowering medication?

**Findings:**

In this post hoc secondary analysis of an extension of a randomized clinical trial including 524 patients, secondary SLT was associated with a reduction in medication load of previously medically treated, stable eyes. Secondary SLT was also associated with additional intraocular pressure control in uncontrolled eyes, but the need for trabeculectomy was not eliminated.

**Meaning:**

These results support the possibility that SLT can replace topical medical therapy in stable eyes and might be used as an additional treatment modality in some uncontrolled eyes.

## Teaser

This secondary analysis of a randomized clinical trial evaluated outcomes after selective laser trabeculoplasty among patients with open-angle glaucoma or ocular hypertension randomized to primary intraocular pressure–lowering eye drops.

## Introduction

Selective laser trabeculoplasty (SLT) is an outpatient procedure used to lower intraocular pressure (IOP) by increasing aqueous outflow through the trabecular meshwork. The use of SLT as a first-line treatment for open-angle glaucoma (OAG) and ocular hypertension (OHT) is supported by robust evidence from randomized clinical trials and meta-analyses,^1,2,3^ adopted by the European Glaucoma Society^4^ and American Academy of Ophthalmology,^5^ and mandated by the National Institute for Health and Care Excellence (NICE).^6^

The efficacy, safety, and cost-effectiveness of primary SLT are well proven, but there are no clear guidelines on the use of SLT as a secondary treatment, ie, after prior use of ocular hypotensive eye drops. It has been suggested that the efficacy of SLT is not affected by the previous use of eye drops^7^; SLT may reduce medication load in medically well-controlled eyes and provide IOP control in medically uncontrolled eyes for a short period and in high-risk populations.^8,9,10^ The usefulness of SLT in delaying the need for incisional glaucoma surgery remains unclear. The aim of this post hoc exploratory analysis is to describe the use of SLT as a secondary treatment following 3 years of protocolized medical treatment to predefined eye-specific IOP targets within a randomized clinical trial.^11^

## Methods

The Laser in Glaucoma and Ocular Hypertension (LiGHT) randomized clinical trial was approved by the City Road and Hampstead Research and Ethics Committee and by the local boards of the participating centers. All patients provided written informed consent before participation; no incentives or compensation for participation were offered. The study was conducted in accordance with good clinical practice guidelines and adhered to the tenets of the Declaration of Helsinki. This study is reported following the Consolidated Standards of Reporting Trials (CONSORT) reporting guideline. Details of the LiGHT trial and the extension design have been described previously^1,11^ and are provided in the trial protocol and statistical analysis plan in Supplement 2.

Newly diagnosed adult patients with previously untreated OAG or OHT were identified at 6 hospitals across the UK. OAG severity was limited to mean deviation visual field (VF) loss not worse than –12 dB in the better eye or –15 dB in the worse eye. Visual acuity was 6/36 or better in the treated eye or eyes, and eyes had no previous intraocular surgery except uncomplicated phacoemulsification at least 1 year before trial entry. Participant demographics were collected, including age, sex, and ethnic origin. Ethnic origin was self-reported and classified as Black, Asian, White, or other. Further details on ethnic origin are reported elsewhere.^1^

Patients were randomized to either primary SLT or primary eye drops using a web-based system. Treatment followed the NICE thresholds at the time^12^ and was incorporated into real-time web-based clinical decision-support software, based on optic disc analysis using Heidelberg retina tomography (Heidelberg Engineering), automated VF assessment with the Humphrey Field Analyzer Mark II Swedish interactive threshold algorithm standard 24-2 (Carl Zeiss Meditec), and IOP measurements (Goldmann applanation tonometry with daily calibration verification). Disease category and severity were specified at baseline.^13,14^ Eye-specific target IOP and participant monitoring intervals were based on the Canadian Target IOP Workshop,^13^ according to the disease severity stratification. The eye-specific target IOP was determined from both an absolute threshold and a percentage reduction (20%, 25%, or 30%, depending on clinical characteristics) from a single untreated baseline IOP measurement.^11^ Treatment escalation followed international guidelines of the European Glaucoma Society,^15^ the American Academy of Ophthalmology Preferred Practice Patterns,^16^ and South East Asia Glaucoma Interest Group at the time.^17^ Treatment was escalated when IOP was above the target IOP by more than 4 mm Hg at a single visit, there was strong evidence of deterioration (VF and/or optic disc^18^) irrespective of IOP, or IOP was above the target by less than 4 mm Hg in the presence of evidence of progression. Measurements influencing treatment decisions (VF, optic disc imaging, and IOP) were made by masked observers.

SLT was delivered according to a predefined protocol, to 360° of the trabecular meshwork, with 100 nonoverlapping shots (25 per quadrant; energy, 0.3-1.4 millijoules).^11^ Up to 2 SLT treatments were permitted; thereafter, medical therapy was initiated, if required. Primary medical treatment included initial single-drug eye drops. Different or additional eye drops were prescribed in the event of a treatment switch (eg, adverse reaction) or treatment escalation (eg, IOP above target or VF and/or disc progression). Drug classes for first-line, second-line, or third-line treatment were defined per NICE^12^ and European Glaucoma Society guidance.^15^

After diagnosis and randomization, participants remained on the allocated primary treatment pathway for 3 years. After the initial 3-year period, participants who received primary eye drops were allowed to cross over, whereby they could choose to have SLT as a treatment switch, ie, OHT/OAG was controlled, but the participant wished to reduce medication load, or as a treatment escalation, ie, OHT/OAG was not controlled (either IOP, VF or optic disc deterioration), and the participant wished to avoid increasing medication load or to delay or avoid surgery. All participants were permitted a total of 3 SLT treatments over the trial’s total 6-year period. Adverse events (AEs) have been reported previously.^1,2^ During the extension of the LiGHT trial, monitoring and follow-up of participants followed the original LiGHT trial protocol.^1,18^ Following SLT, eye drop reduction was done according to a prespecified protocol: participants switching to SLT were advised to stop 1 drug 2 weeks before the 2-month follow-up appointment after SLT. Participants using 2 or more drugs were recommended to stop the drug added last into their regimen or the 1 causing the most AEs. Medication was reintroduced according to clinical need, following the LiGHT trial protocol for treatment escalations. The LiGHT trial protocol did not include minimally invasive glaucoma surgery with or without cataract surgery or tube shunt procedures.

### Statistical Analysis

The analyses presented here were not included in the trial’s initial statistical analysis plan and are thus post hoc exploratory.^19^ Formal statistical tests were carried out for some comparisons. *P* values were 2-sided, and statistical significance was set at *P* ≤ .05. The *P* values are not corrected for multiple comparisons. All analyses were performed in Stata version 14 (StataCorp). Data were analyzed from February 2021 to December 2024.

## Results

**Figure 1.**
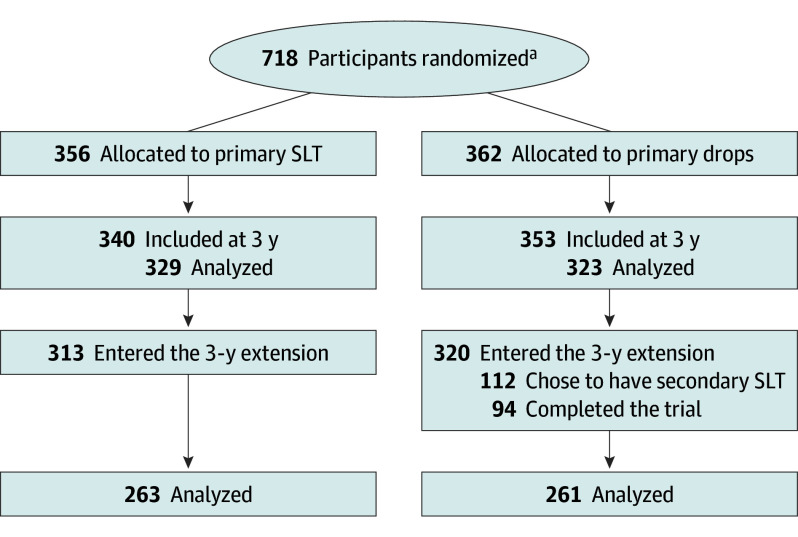
Laser in Glaucoma and Ocular Hypertension Randomized Clinical Trial Participant Recruitment Flowchart SLT indicates selective laser trabeculoplasty. ^a^Two participants were initially randomized twice due to a software failure, where the initial randomization was not visible and subsequently a second randomization was carried out. One of these participants was initially randomized to medication but was subsequently randomized to, and received, SLT. The other was initially randomized to SLT but was subsequently randomized to, and received, medication. These participants are included in the diagram according to the second randomizations.

A total of 718 participants were randomized for the initial LiGHT trial; 329 participants receiving primary SLT and 323 participants receiving primary eye drops contributed to the final analysis of the original LiGHT trial. A total of 633 participants (88.2%) entered the 3-year study extension; of those, 313 participants (547 eyes) had received primary SLT and 320 participants (549 eyes) received primary eye drops. A total of 524 participants (73.3%; 910 eyes) completed the study extension (72 months) (Figure 1**)**.

Of 320 participants receiving primary eye drops, 112 participants (35%; 176 eyes) chose to receive SLT after the end of the 3-year monitoring period (Figure 1; eFigure 1 and eTable 1 in Supplement 3); 70 participants (115 eyes) switched to SLT to reduce medication load (median [IQR] 42.7 [38.6-46.4] months from baseline), and 29 participants (35 eyes) chose to have SLT as a treatment escalation, ie, due to uncontrolled IOP and/or disease progression (median [IQR] 42.4 [37.2-50.5] months from baseline). Another 13 participants (26 eyes) had SLT as a treatment escalation in 1 eye and as a treatment switch in the other eye. Of 112 participants who received secondary SLT, 94 (83.9%) completed the study extension to 72 months. Participants continuing with primary eye drops (ie, choosing not to have secondary SLT) were comparable to those switching or escalating to SLT, as well as those initially treated with SLT in terms of their sex, ethnic origin, family history of glaucoma, and diagnosis (eTable 2 in Supplement 3). The mean (SD) age of the participants continuing with eye drops only was 63.4 (11.7) years, those having secondary SLT 62.7 (11.0) years, and those who had primary SLT was 63.1 (12.0) years, and there were more men than women (112 men [54.8%], 58 men [51.8%], and 178 men [56.8%], respectively). Participants across groups were similar by ethnic origin (eTable 2 in Supplement 3).

Baseline ocular variables were also overall comparable among the eyes that continued primary eye drops, those that received secondary SLT after 3 years of medical treatment, and those that had primary SLT (Table 1). At the time of secondary SLT, eyes that had SLT as a treatment escalation overall had more severe disease than those with SLT as a treatment switch; 20 of 48 eyes with treatment escalation (41.7%) had moderate or severe OAG, compared with 22 of 128 eyes with treatment switch (17.2%) (eTable 1 in Supplement 3). Eyes that had SLT as a treatment escalation were also receiving more intense medical treatment compared with eyes that had SLT as a treatment switch (mean [SD], 1.94 [0.83] drugs [ie, active ingredients] vs 1.38 [0.62] drugs; mean difference, 0.56 [95% CI, 0.30 to 0.82] drugs; *P* < .001) (Table 2). Most eyes receiving secondary SLT after primary eye drops had a single SLT treatment (120 of 128 eyes that switched [93.8%] and 44 of 48 eyes that escalated [91.7%]) (Table 2).

**Table 1.** Diagnosis and Ocular Characteristics at Trial Initiation for the Eyes Continuing With Eye Drops and Eyes Receiving Secondary SLT After a Period of at Least 3 Years and Eyes Receiving Primary SLT

Characteristic	Eyes, No. (%)		
	Primary medical treatment (n = 549 eyes; 320 participants)		Primary SLT (n = 547 eyes; 313 participants)
	Eye drops only (n = 373 eyes)	Eye drops then SLT (n = 176 eyes)	
Diagnosis at trial initiation			
Ocular hypertension	107 (28.7)	53 (30.1)	168 (30.7)
Mild OAG	199 (53.3)	96 (54.5)	280 (51.2)
Moderate OAG	44 (11.8)	21 (11.9)	60 (11.0)
Severe OAG	23 (6.2)	6 (3.4)	39 (7.1)
Refractive error, mean (SD), spherical D	−0.3 (2.9)	−0.1 (2.6)	−0.5 (3.2)
Visual acuity, mean (SD)	0.1 (0.1)	0.0 (0.1)	0.1 (0.2)
Visual field mean deviation, mean (SD), dB	−2.9 (3.6)	−2.9 (3.1)	−3.0 (3.4)
HRT rim area, mean (SD), mm^2^	1.2 (0.4)	1.1 (0.4)	1.2 (0.4)
Intraocular pressure, mean (SD), mm Hg	24.1 (5.2)	24.9 (4.8)	24.5 (5.2)
CCT, mean (SD), μm	552.7 (36.6)	550.2 (36.3)	550.1 (39.1)
Pseudoexfoliation	8 (2.1)	4 (2.3)	4 (0.7)
Pseudophakia	16 (4.3)	11 (6.3)	32 (5.9)

Abbreviations: CCT, central corneal thickness; OAG, open-angle glaucoma; SLT, selective laser trabeculoplasty.

**Table 2.** Treatment Outcomes for Eyes Switching and Escalating to SLT After a Period of at Least 3 Years, Compared With Eyes That Continued IOP-Lowering Eye Drops and Those Treated With Primary SLT

Treatment	Eyes, No. (%)				*P* value
	Primary IOP-lowering eye drops			Primary SLT (n = 547)	
	Eye drops only (n = 373)	Switch to SLT (n = 128)	Escalation to SLT (n = 48)		
Target IOP at 72 mo	282 (94.9)	108 (94.7)	39 (92.9)	437 (94.2)	.16^a^
SLT treatments, No.					
1	0	120 (93.8)	44 (91.7)	343 (62.7)	NA
2	0	8 (6.3)	2 (4.2)	169 (30.9)	NA
3	0	0	2 (4.2)	32 (5.9)	NA
4	0	0	0	3 (0.5)	NA
Treatment intensity per eye at target IOP at 72 mo					
No medications	21 (6.9)	72 (63.2)	13 (31.0)	338 (71.9)	NA
No medications and no surgery	10 (3.3)^b^	69 (60.5)	4 (9.5)^c^	328 (69.8)	<.001^a^
1 Drug^d^	171 (56.3)	18 (15.8)	7 (16.7)	56 (11.9)	NA
2 Drugs^d^	62 (20.4)	12 (10.5)	13 (31.0)	31 (6.6)	NA
3 Drugs^d^	25 (8.2)	6 (5.3)	6 (14.3)	11 (2.3)	NA
4 Drugs^d^	3 (1.0)	0	0	1 (0.2)	NA
Drugs used, No.^d^					
Before SLT					
Mean (SD)	NA	1.38 (0.62)	1.94 (0.83)	NA	<.001^e^
Median (IQR)	NA	1 (1 to 2)	2 (1 to 2)	NA	
At 72 mo					
Mean (SD)	1.43 (0.78)	0.59 (0.92)^f^	1.73 (1.01)	0.40 (0.79)^f^	<.001^g^
Median (IQR)	1 (1 to 2)	0 (0 to 1)	2 (1 to 2)	0 (0 to 1)	
*P* value (pre/post SLT)		<.001^h^	.17^h^		
Trabeculectomy^i^	20 (5.4)	3 (2.3)	9 (18.7)	13 (2.4)	<.001^a^
Visual acuity	0.1 (0.2)	0.1 (0.2)	0.1 (0.1)	0.1 (0.2)	
IOP	15.4 (3.8)	15.9 (3.5)	14.6 (5.3)	16.3 (4.0)	
Mean deviation	−3.8 (4.4)	−3.7 (4.2)	−5.3 (4.6)	−4.0 (4.5)	

Abbreviations: IOP, intraocular pressure; NA, not applicable; SLT, selective laser trabeculoplasty.

^a^ Calculated using χ^2^ test.

^b^ Two eyes had clear lens extraction due to angle closure that developed during the trial, and 8 eyes had phacoemulsification surgery.

^c^ All 4 eyes had a response to SLT that made the use of medical treatment redundant.

^d^ *Drugs* refers to active components.

^e^ Rank sum test.

^f^ The mean number of medications at 72 months was statistically different between primary SLT eyes and eyes that switched to SLT (rank sum test *P* = .03). Seven eyes in the eye drops only group and 6 eyes in the primary SLT group were missing at-target information.

^g^ Calculated using Kruskal-Wallis test.

^h^ Wilcoxon signed rank test.

^i^ Excluding eyes that had trabeculectomy at any point during the trial.

**Figure 2.**
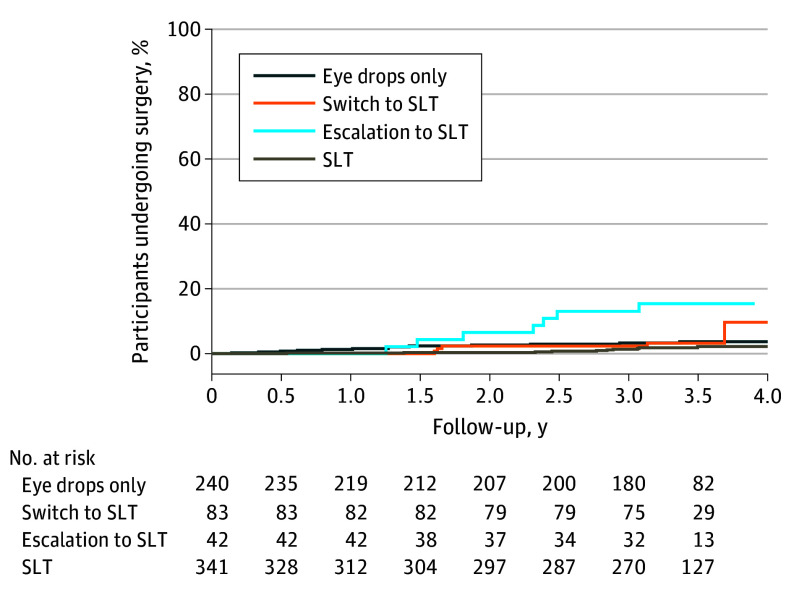
Kaplan-Meier Plot of Time to Surgery During the Study’s Extension Eyes receiving primary medical treatment have been split into eye drops only, escalation to selective laser trabeculoplasty (SLT), and switch to SLT; the unit of analysis is by eye. Month 0 signifies the initiation of the study’s extension and the offer of SLT; month 36 signifies the end of the study’s extension (the total trial monitoring period is 6 years).

Of eyes that switched to SLT to reduce medication load after 3 years of primary eye drops, 69 (60.5%) were both free of eye drops and needed no surgery at the end of the trial follow-up (Table 2). Of eyes receiving 1 drug before switching to SLT, 62 (83.8%) needed no medical treatment at the end of the trial, and there was no change in the medical treatment of 9 eyes (12.2%) (Table 3). Topical medication was reduced in 15 eyes that switched to SLT while using 2 drugs (60.0%) and in 2 eyes that switched to SLT while using 3 drugs (33.4%), although this result should be treated with caution due to the limited number of eyes in this group (Table 3). Protocolized eye drop reduction for eyes controlled with primary medical treatment, subsequently switching to SLT and needing no medical or surgical treatment at 72 months, maintained 33.9% lower IOP than baseline at a mean of 2.4 (0.7) years after switching to SLT (eTable 3 in Supplement 3). Eyes that escalated to SLT had a mean IOP reduction of 4.6 mm Hg (21.8%) (eTable 5 in Supplement 3). Of eyes escalating to SLT, 30 (62.5%) reached target IOP at the end of the trial without the need for surgery (eTable 4 in Supplement 3), while 9 eyes (18.7%) needed a trabeculectomy during the trial (Table 2 and Figure 2).

**Table 3.** Treatment Intensity of the Eyes Switched to SLT After Primary Medical Treatment That Were at Target Intraocular Pressure at 72 Months Without Having Undergone a Trabeculectomy, by Treatment Intensity Before SLT

Drugs used before SLT	Eyes, No.	Eyes at 72 mo, No. (%)			
		Needing no medical treatment	With 1 drug	With 2 drugs	With ≥3 drugs
1 Drug	74	62 (83.8)	9 (12.2)	2 (2.7)	1 (1.4)
2 Drugs	25	6 (24.0)	9 (36.0)	9 (36.0)	1 (4.0)
≥3 Drugs	6	1 (16.7)	0	1 (16.7)	4 (66.7)

Abbreviation: SLT, selective laser trabeculoplasty.

Before receiving SLT, patients used a mean (SD) of 1.38 drugs (ie, active components) for the eyes switched to SLT. After SLT, they used a mean (SD) of 0.59 (0.92) drugs at the end of the monitoring period (at 72 months) (mean difference, 0.79 [95% CI, 0.66 to 0.93] drugs; *P* < .001). The number of medications between eyes having primary SLT and eyes switched to SLT were different at 72 months (mean difference, −0.19 [95% CI, −0.36 to −0.02] drugs; *P* = .03) (Table 2; eFigure 2 in Supplement 1). Eyes that had SLT as a treatment escalation did not have a significant reduction in drugs used, from a mean (SD) of 1.94 (0.83) drugs to 1.73 (1.01) drugs (mean difference, −0.21 [95% CI, −0.52 to 0.10] drugs; *P* = .17) (Table 2). Four eyes did not need eye drops following escalation to SLT.

Visual acuity was comparable across all subgroups. At the end of the 6-year monitoring period, eyes that had escalated to SLT had more advanced VF loss and lower IOP than other groups (mean deviation [SD], −5.3 [4.6] dB vs −3.8 [4.4] dB for eyes using primary eye drops only; −3.7 [4.2] dB for eyes that switched to SLT; −4.0 [4.5] dB for eyes that received primary SLT; mean [SD] IOP, 14.6 [5.3] mm Hg vs 15.4 [3.8] mm Hg for eyes using primary eye drops only; 15.9 [3.5] mm Hg for eyes that switched to SLT; 16.3 [4.0] mm Hg for eyes that received primary SLT) (Table 2). SLT adverse events and rates of cataract surgery have been reported previously.^2^

## Discussion

In this post hoc exploratory analysis of the LiGHT randomized clinical trial, of 320 participants entering the 3-year study extension initially treated with eye drops, 112 (35.0%) chose to have SLT after 3 years. Eye drop freedom, treatment burden, and related ocular AEs have been previously identified as important to participants^20,21^ and may have been drivers for participants to alter their therapeutic regimen in this trial. We found that SLT was associated with discontinuation of eye drops or reduced medication load for at least 3 years in eyes with mild disease that was well controlled with medical treatment. These findings also suggest that SLT can be an alternative to additional eye drops in eyes needing further IOP reduction while already using eye drops (treatment escalation) but may not prevent the need for trabeculectomy in eyes with more advanced disease.

When SLT was offered as a treatment switch in disease controlled by eye drops, 69 of 114 eyes (60.5%) achieved freedom from eye drops nearly 3 years after laser treatment, without the need for glaucoma surgery. A switch to SLT was also associated with a reduction in medication and 79 of 105 eyes (72.5%) used fewer eye drops. Disease control without eye drops and reduced total medication need were more often achieved in eyes requiring less intensive medical treatment at the time of SLT; 62 of 74 eyes receiving 1 drug (84%) were eye drop–free after SLT, while medications load was reduced in 15 of 25 eyes receiving 2 drugs (60%) and in 2 of 6 eyes receiving 3 drugs (33%); this was associated with a single SLT treatment in 120 of 128 eyes switched to SLT (94%). Eyes switched to SLT after 3 years of primary eye drops had a medication load that was clinically comparable to eyes initially treated with primary SLT. The number of medications between eyes having had primary SLT and eyes switched to SLT were different, and it is possible that primary SLT offers a lighter medication load compared with secondary SLT. The difference between the groups was 0.19 medications; however, this is a small reduction that should be interpreted with caution.

With the cost of glaucoma prescriptions in England reaching £114.2 million (approximately US $139.7 million) in 2018,^22^ switching to SLT may promote more cost-effective services for the NHS and the large numbers of patients still treated medically. Treatment burden has been well defined by participants in qualitative studies^21,23,24^ and is now understood to differ from the burden of the disease itself. As such, where relevant, clinicians should be aware of the difficulties posed by daily treatments and offer alternatives to medical therapy.

This study found that secondary SLT was associated with IOP lowering in eyes controlled with eye drops (33.9% reduction), likely comparable to primary laser treatment (31.4% reduction),^25^ and compatible with the IOP reduction requirements adopted by landmark medical and surgical clinical trials, such as the Ocular Hypertension Treatment Study, Early Manifest Glaucoma Trial, and Collaborative Initial Glaucoma Treatment Study.^13,26,27,28^ Eyes with advanced and/or progressing disease requiring very low IOPs are more likely to need trabeculectomy^29^ to reduce VF progression,^30^ and clinicians might be cautious attempting to use SLT to avoid incisional glaucoma surgery.

Eyes escalated to SLT, because IOP was above the target range and/or because glaucoma was progressing, had more severe disease and more intensive treatment at the time of laser than eyes having SLT as a treatment switch. Escalation to SLT was not associated with a reduction of the medical treatment intensity. Our findings suggest that secondary SLT can lead to adequate IOP control in some of eyes progressing or uncontrolled with eye drops (30 of 48 eyes reaching the end of the trial [62.5%]). Escalating treatment with SLT in these eyes may have delayed the need for trabeculectomy for some, but surgery was still required during the trial’s duration for almost 1 in 5 of these eyes; this might to be considered by clinicians and communicated to participants before offering SLT as a therapeutic option.

In total, 120 of 128 eyes switching to secondary SLT (93.8%) and 44 of 48 eyes escalating to SLT (91.7%) had a single SLT treatment, while there was a wider distribution of the number of SLT treatments performed in eyes receiving primary SLT. As the crossover was only possible after 3 years, participants who received primary SLT had a longer period of follow-up and possibly more time to need a second or third SLT. Patients’ experience and sense of security with eye drops cannot be excluded for those in the primary eye drops group having secondary SLT.

This study follows from previously published clinical trial data on the outcomes of primary SLT in reducing IOP without the need for eye drops in nearly 70% of treated eyes^2^; similar efficacy has been reported by other clinical trials.^3,10,31^ Previous LiGHT trial data also support SLT is safe, with only 1% of laser treatments leading to an IOP spike and no serious laser-related AEs in nearly 1000 procedures.^2^ SLT has been shown to have greater efficacy in eyes with higher baseline IOP^32,33^; recently, the association between baseline IOP and the probability of more than 20% IOP reduction was found to be different between SLT and prostaglandin analogue eye drops, with SLT being more successful at baseline IOP greater than 22.5 mm Hg.^34^ Lower SLT success rates have been reported by Khawaja et al^35^ in a mixed cohort of patients with low pre-SLT IOP (22.0 mm Hg) and predominantly failing IOP control in medicated eyes, based on retrospective data with inconsistent follow-up. Such data may reflect the complexities of drawing conclusions on efficacy from mixed populations and/or using SLT as a treatment escalation, as shown in this study.

### Limitations

This study has some limitations. The analyses presented here are exploratory and are proposing hypotheses not yet tested in a designed-for-purpose trial. Exploratory data can provide insight into the subpopulations for which a trial may not be clinically or financially feasible.^36^ This study reports data on a limited number of eyes, particularly those with advanced glaucoma and/or escalating with secondary SLT, and results should be interpreted with caution. For those eyes, it is possible that regression to the mean may have led to an overestimation of the treatment effect.

## Conclusions

The findings of this post hoc secondary analysis of a randomized clinical trial suggest that SLT in stable, medically treated eyes was associated with eye drop–free IOP and disease control for at least 3 years with a single treatment in most eyes with primarily mild disease and reduced medication intensity in many more. In eyes with uncontrolled IOP, this analysis supports the possibility that adjunctive SLT could take the place of additional medication, but for eyes with progressive glaucoma or advanced disease, SLT might not prevent the need for definitive surgical pressure lowering.

## Notes

Supplement 1.
The LiGHT Trial Study Group

Supplement 2.
Trial Protocol and Statistical Analysis Plan

Supplement 3. **eFigure 1.** Histogram of Timing of the Secondary SLT Treatments in eyes That Had Originally Received Primary Medical Treatment (IOP-Lowering Eye-Drops) **eFigure 2.** Medication-Load Profile at 6 Years for the Eyes at Target That Were Switched to SLT Following Primary Medical Treatment Compared to the Eyes That Continued Medical Treatment Alone **eTable 1.** Timing and Disease Severity at the Time of Secondary SLT for Eyes That Switched to SLT and Eyes That Escalated to SLT After a Period of at Least 3 Years in Primary Medical Treatment **eTable 2.** Characteristics for the Participants Remaining on IOP Lowering Eye-Drops or Receiving Secondary SLT After a Period of at Least 3 Years and Those Receiving Primary SLT **eTable 3.** Mean and Percentage IOP Reduction (From Baseline) for the 69 Eyes Switched to SLT After Primary Medical Treatment, Needing No Medication or Surgery at the End of the Trial **eTable 4.** Number of Eyes Reaching Target IOP at the End of the Trial and Need for Subsequent Trabeculectomy in Eyes Escalating to SLT, by Treatment Intensity Before Escalation **eTable 5.** Absolute and Percentage IOP Reduction Before and After SLT for Eyes That Escalated to SLT

Supplement 4.
Data Sharing Statement
