# Supplementary material for: Selective Laser Trabeculoplasty After Medical Treatment for Glaucoma or Ocular Hypertension
Source: JAMA Ophthalmol. 2025 Feb 20;143(4):295–302. doi: 10.1001/jamaophthalmol.2024.6492 (PMC11843460; doi:10.1001/jamaophthalmol.2024.6492)
Supplement: Supplement 1. — The LiGHT Trial Study Group [file jamaophthalmol-e246492-s001.pdf]

Supplemental Online Content: Nonauthor Collaborators
*First name, last name, and suffix (if applicable) are required and will appear in PubMed.

***Group Name(s): the LiGHT Trial Study Group**

*First Name and Middle Initial(s)	*Last Name	*Suffix (eg, Jr, III)	Academic Degrees	Institution	Location (city, state/province, country)	Role or Contribution, eg, chair, principal investigator	Group (if more than 1 Group listed in the byline) and/or Subgroup (eg, Steering Committee)
Rupert	Bourne			Hinchingbrooke Hospital		Local PI	
David	Broadway			Norfolk & Norwich University Hospital		Local PI	
Marta	Buszewicz			University College London		Trialist, co-investigator	
David	Crabb		PhD	City University London		Vf analysis	
Amanda	Davis		PhD	Moorfields Eye Hospital NHS Foundation Trust		Trial manager	
Anurag	Garg		MD	Guy's and St Thomas' NHS Foundation Trust		Trail fellow	
Daniel	Hornan					Local PI	
Rachael	Hunter			University College London		Health economist	
Hari	Jayaram			Moorfields Eye Hospital NHS Foundation Trust		Local PI	
Yuzhen	Jiang						
Sheng	Lim			Guy's and St Thomas' NHS Foundation Trust		Local PI	
Joanna	Liput			York Teaching Hospital		Local PI	
Timothy	Manners					Local PI	
Giovanni	Montesano		PhD	Moorfields Eye Hospital NHS Foundation Trust		Trial fellow	
Stephen	Morris			University College London		Co-investigator	
Giovanni	Ometto		PhD	City University London		VF analysis	
Gary	Rubin			UCL Institute of Ophthalmology		Co-investigator	
Nicholas	Strouthidis		PhD	Moorfields Eye Hospital NHS Foundation Trust		Local PI	
Sarah	Wilson			Belfast Health and Social Care Trust		Local PI	
Richard	Wormald			Moorfields Eye Hospital NHS Foundation Trust		Co-investigator	
David	Wright		PhD	Queen's University Belfast		VF analysis	
Haogang	Zhu		PhD			Algorithm developmen	
