# Supplementary material for: Selective Laser Trabeculoplasty After Medical Treatment for Glaucoma or Ocular Hypertension
Source: JAMA Ophthalmol. 2025 Feb 20;143(4):295–302. doi: 10.1001/jamaophthalmol.2024.6492 (PMC11843460; doi:10.1001/jamaophthalmol.2024.6492)
Supplement: Supplement 2. — Trial Protocol and Statistical Analysis Plan [file jamaophthalmol-e246492-s002.pdf]

NIHR HTA Programme

18 October 2013

The NIHR Evaluation, Trials and Studies Coordinating Centre (NETSCC), based at the University of Southampton, manages evaluation research programmes and activities for the NIHR

Health Technology Assessment Programme
National Institute for Health Research
Evaluation, Trials and Studies Coordinating Centre
University of Southampton, Alpha House
Enterprise Road, Southampton, SO16 7NS

tel: +44(0)23 8059 5586
fax: +44(0)23 8059 5639

email: hta@hta.ac.uk
web: www.hta.ac.uk

LiGHT Trial Protocol 2.0, 31st March 2013

Full title of trial

“Health-Related Quality of Life in two treatment pathways for newly diagnosed open angle glaucoma and ocular hypertension: an unmasked, multi-centre, randomised controlled trial of initial selective laser trabeculoplasty versus conventional medical therapy.”

Short title: Laser-1st vs Drops-1st for Glaucoma and Ocular Hypertension

Version and date of protocol: Version 1.4 of “LiGHT Trial” Protocol, 25th June 2012

Sponsor: Moorfields Eye Hospital NHS Foundation Trust

Sponsor protocol number: GAZG1001

Funder: National Institute of Health Research Health Technology Assessment Panel (NIHR HTA)

Funder’s reference: HTA 09/104/40 - LiGHT

Clinical Trial no: ISRCTN32038223

Central Coordinating Trial Site: Moorfields Eye Hospital NHS Foundation Trust

Collaborating Sites:

- Belfast Health and Social Care Trust
- Norfolk and Norwich NHS Foundation Trust
- St Thomas’ NHS Foundation Trust
- Hinchinbrook Health Care NHS Trust
- York Teaching Hospital NHS Foundation Trust

Chief investigator: Gus Gazzard MA MD FRCOphth

Consultant Ophthalmic Surgeon,

Moorfields Eye Hospital Foundation NHS Foundation Trust,

City Rd, London, EC1V 2PD

Signatures

The Chief Investigator and the Moorfields R&D unit have discussed this protocol. The investigators agree to perform the investigations and to abide by this protocol except in case of medical emergency (see section 9.4.6 for the recording and reporting of deviations, violations, potential serious breaches, serious breaches and urgent safety measures) or where departures from it are mutually agreed in writing.

The investigator agrees to conduct the trial in compliance with the protocol, GCP and UK Regulations, the Data Protection Act (1998), the Trust Information Governance Policy (or other local equivalent), the Research Governance Framework (2005), the Sponsor's SOPs, and other regulatory requirements as appropriate.

Chief investigator

Gus Gazzard

Moorfields Eye Hospital
NHS Foundation Trust

Signature ____________________ Date ____________________

Sponsor Representative

Maria Hassard

Moorfields Eye Hospital
NHS Foundation Trust

Signature ____________________ Date ____________________

Contents

"Health-Related Quality of Life in two treatment pathways for newly diagnosed open angle glaucoma and ocular hypertension: an unmasked, multi-centre, randomised controlled trial of initial selective laser trabeculoplasty versus conventional medical therapy." 1

Signatures 2

List of abbreviations 9

1 Trial personnel 11

Summary 13

2 Introduction 15

2.1 Background: the burden of the problem & its importance to the NHS 15

2.2 Investigational medicinal product(s): Not applicable 16

2.3 Preclinical data: Not applicable 16

2.4 Clinical data: Not applicable 16

2.5 Rationale and risks/benefits 16

2.5.1 The condition and its treatment 16

2.5.2 Why choose SLT as a first treatment for OAG/OHT? 17

2.5.3 Benefits to society and Generalisability 18

2.6 Assessment and management of risk 19

3 Objectives 19

3.1 Hypotheses 19

3.2 Primary: 20

3.3 Secondary: 20

4 Trial design 20

4.1 Overall design 20

4.2 Recruitment of participants 21

4.3 Proposed duration of the intervention 21

4.4 Justification of unmasked study design 21

4.5 Methods to protect against other sources of bias 22

5 Selection of Subjects 22

5.1 Inclusion criteria 22

5.1.1 Diagnosis of OAG 22

5.1.2 Diagnosis of OHT 23

5.2 There are no exclusions based upon concurrent medical treatments 24
5.3 Concomitant medication 24
6 Recruitment 24
6.1 Methods used to ensure recruitment to the study 25
6.2 Recruiting centres & new patient numbers 25
6.2.1 Poll of new glaucoma patients at Moorfields 25
6.2.2 Rates of medical/laser interventions for new patients 25
6.2.3 Recruiting centres & new patient numbers 25
6.3 Internal Pilot Study 26
6.3.1 Strategic plan for unanticipated low-recruitment: 26
7 Study procedures and schedule of assessments 27
7.1 Informed consent procedure 27
7.2 Randomisation and allocation of participants to trial groups 28
7.3 Emergency un-blinding 28
7.4 Screening assessments 28
7.5 Baseline assessments 28
7.6 Treatment procedures 29
7.6.1 Standardisation of Interventions & Incremental Escalation of Treatment 29
7.6.2 Trial arm 1 : "Laser-1st" Pathway 29
7.6.3 Trial arm 2: "Medicine-1st" Pathway 29
7.7 Computerised decision algorithm in OpenEyes 30
7.8 Setting Individualised Patient Treatment-Targets 31
7.9 Treatment Changes 32
7.9.1 Definition of 'Failure to Meet Target' 32
7.9.2 Process for Treatment-Target Reassessment 33
7.9.3 Treatment escalation to glaucoma surgery 33
7.10 Detection of Progressive Glaucoma Damage 33
7.10.1 Visual Field Progression 33
7.10.2 Optic Disc Progression 34
7.11 Proposed frequency and duration of follow-up 34
7.11.1 Follow up procedure 34
7.12 Subsequent assessments 36

7.13 Notes on Clinical Assessments 37
7.14 Table of study assessments 37
7.15 Methods 38
7.14.1 Training of researchers and collection of data 38
7.14.2 Laboratory procedures 38
7.14.3 Radiology or other procedures 38
7.15 Gantt Chart 38
7.16 Definition of end of trial 38
7.17 Discontinuation/withdrawal of participants and ‘stopping rules’ 38
8 Name and description of all drugs used in the trial 39
8.1 ‘Name and description of each IMP’ 39
8.2 ‘Source of IMPs including placebo’ 39
8.3 ‘Accountability procedures for the IMP, including placebo/comparator’ 39
8.4 ‘Route of administration, dosage, dosage regimen, and treatment period of the IMP’ 39
8.5 Dose modifications 39
8.6 Assessment of compliance 40
8.7 Post-trial IMP arrangements 40
8.8 Name and description of each NIMP 40
9 Recording and reporting of adverse events and reactions 42
9.1 Definitions 42
9.2 ‘Expected Adverse Events’ 42
9.2.1 Prostaglandin analogues (PGA): 42
9.2.2 Beta blocker (once in the morning or in a PGA combination): 43
9.2.3 Topical carbonic anhydrase inhibitor (CAI): 43
9.2.4 Alpha-agonist: 43
9.2.5 Anti-inflammatories- steroids: 43
9.2.6 Anti-inflammatories- NSAIDs: 43
9.2.7 Antibiotics: 43
9.2.8 Acetazolamide: 43
9.2.9 Any preservative-free topical lubricants (‘artificial tears’): 43
9.2.10 SLT: intra-ocular pressure spike: 43
9.3 Recording adverse events 44

9.4 Procedures for recording and reporting Serious Adverse Events 45
9.4.1 Notification of deaths 45
9.4.2 Reporting SUSARs 45
9.4.3 Annual safety reports 45
9.4.4 Annual progress reports 46
9.4.5 Pregnancy 46
9.4.6 Reporting Urgent Safety Measures 46
9.4.7 Notification of Serious Breaches to GCP and/or the protocol 46
9.5 The type and duration of the follow-up of subjects after adverse events. 47
10 Data management and quality assurance 47
10.1 Confidentiality 47
10.2 Data collection tools and data handling 48
11 Record keeping and archiving 48
12 Statistical Considerations 49
12.1 Outcome Measures 49
12.1.1 Primary Outcome Measure: Health-related Quality of Life 49
12.1.2 Secondary Outcome Measures 50
12.2 Sample size and recruitment 53
12.2.1 Sample size calculation 53
12.2.2 Planned recruitment rate 54
12.3 Statistical analysis plan 54
12.3.1 Summary of patient flow 54
12.3.2 Primary endpoint analysis 54
12.3.3 Secondary endpoint analysis 55
12.4 Randomisation 55
12.5 Interim analysis 56
12.6 Other statistical considerations 56
13 Committees involved in LiGHT 56
13.1 Study Co-Ordination In London (Central Trial Office) 56
13.2 Local Organisation In Centres 57
13.3 Trial Steering Committee (TSC) 57
13.4 Data and Safety Monitoring (DMC) 57

14 PPI and Lay Advisory Group ~ Service User Input 58
15 Direct Access to Source Data/Documents 58
16 Ethics and regulatory requirements 58
17 Monitoring plan for the trial 59
18 Finance 60
19 Insurance 60
20 Statement of compliance 60
21 Greenhouse Gas Emissions 60
22 Protocol Training Log 61
23 PI's Log of Protocol &/or GCP Deviations/Violations/Potential serious breaches/Serious breaches/Urgent safety measures 63
24 References 65
25 Appendices Contents 77
26 Appendix 1 ~ Trial Design Flow-chart 78
27 Appendix 2 ~ Predicted recruitment rate 79
28 Appendix 3 ~ Stratification of Patients by Severity of Their Disease 80
29 Appendix 4 ~ Frequency of Follow-Up According to Severity of Disease 82
30 Appendix 5 ~ Details of Treatment 83
30.1 Appendix 5a ~ Trial arm 1: "Laser-1st" Pathway, SLT 83
30.2 Appendix 5b ~ Trial arm 2: "Medicine-1st" Pathway, Drops 85
30.2.1 Maximum Medical Therapy – MMT 85
30.2.2 Choice of Agent 85
30.2.3 To Add or Switch Medicines? 86
30.2.4 Training and Education in Drop Treatment 86
30.2.5 Other Ophthalmic Medications 87
30.3 Appendix 5c ~ Details of Other Surgical Treatments 88
31 Appendix 6 ~ Changing Treatment & Defining Failure 90
31.1 Definition of 'Failure to Meet Target' (See flow-charts, Appendix 8) 90
31.2 Process for Treatment-Target Reassessment 91
31.3 Treatment escalation to glaucoma surgery 91
32 Appendix 7 ~ Detection of Progressive Glaucoma Damage 93
32.1 Visual Field Progression 93

32.2 Optic Disc Progression 93

32.3 Resetting of Visual Field and Optic Disc Baselines........ 94

32.4 Unreliable or Unavailable VF & HRT – dealing with missing data........ 94

32.4.1 Missing VF/HRT Data........ 94

32.4.2 Unreliable VF/HRT Data........ 94

33 Appendix 8 ~ How to set the Treatment Target IOP 95

34 Appendix 9 ~ If Target is not met: when to escalate treatment and when to reassess Target for OHT (9a) and POAG (9b) 97

34.1 Appendix 9a ~ If Target is not met (OHT) : when to escalate treatment and when to reassess Target........ 99

34.2 Appendix 9b ~ If Target is not met (POAG): *when* to escalate treatment and when to reassess Target........ 100

35 Appendix 10 ~ *How* to Escalate Treatment........ 101

36 Appendix 11 ~ Questionnaires........ 102

36.1 Delivery and Follow-up 102

36.2 Questionnaire Content........ 102

36.2.1 EQ-5D [74] 102

36.2.2 Glaucoma Utility Index [83] (GUI)........ 104

36.2.3 Glaucoma Symptom Scale [84] (GSS) 104

36.2.4 Glaucoma Quality of Life - 15 [8] (GQL-15) 105

36.2.5 Client Services Receipt Inventory [99;100] (CSRI) 106

36.2.6 Concordance / Compliance........ 106

37 Appendix 12 Project Gantt Chart 108

38 Appendix 13 “Guidelines on questions to ask in clinic for clinical management”
109

39 Appendix 14 " Video Script"........ 109

List of abbreviations

AA	Alpha Agonist
AAO	American Academy of Ophthalmology
AE	Adverse Event
AGIS	Advanced Glaucoma Intervention Study
ALT	Argon Laser Trabeculoplasty
AMD	Age related macular degeneration
AR	Adverse Reaction
ASR	Annual Safety Report
CA	Competent Authority
CAI	Carbonic Anhydrase Inhibitor
CCT	Central Corneal Thickness
CDR	Cup Disc Ratio
CERA	Centre for Eye Research, Australia
CGS	Canadian Glaucoma Study
CI	Chief Investigator
CIGTS	Collaborative Initial Glaucoma Treatment Study
CNTGS	Collaborative Normal Tension Glaucoma Study
CRF	Case Report Form
CRO	Contract Research Organisation
CSRI	Client Service Receipt Inventory
CTA	Clinical Trial Authorisation
CTIMP	Clinical Trial of Investigational Medicinal Product
DMC	Data Monitoring Committee
EAGLE	Evaluation of Angle Closure Lens Extraction
EC	European Commission
EGPS	European Glaucoma Prevention Study
EGS	European Glaucoma Society
EMEA	European Medicines Agency
EMGT	Early Manifest Glaucoma Treatment Study
EQ-5D	EuroQol questionnaire
EU	European Union
EUCTD	European Clinical Trials Directive
EudraCT	European Clinical Trials Database
EudraVIGILANCE	European database for Pharmacovigilance
GAfREC	Governance Arrangements for NHS Research Ethics
GAT	Goldmann Applanation Tonometry
GCP	Good Clinical Practice
Glau-QoL	Quality of Life in Glaucoma Questionnaire
GMC	General Medical Council
GMP	Good Medical Practice
GON	Glaucomatous Optic Neuropathy
HRQL	Health-Related Quality of Life
HTA	Health Technology Assessment panel
HVF	Humphrey Visual Field
IB	Investigator Brochure
ICF	Informed Consent Form
ICT	Irido-trabecular contact
IGA	International Glaucoma Association
IMP	Investigational Medicinal Product
IMPD	Investigational Medicinal Product Dossier
IOP	Intra-Ocular Pressure
ISF	Investigator Site File
ISRCTN	International Standard Randomised Clinical Trial Number

LT	Laser Trabeculoplasty
MA	Marketing Authorisation
MAI	Massof Activity Index
Main REC	Main Research Ethics Committee
MD	Mean Deviation
MHRA	Medicines and Healthcare products Regulatory Agency
MMC	Mitomycin C
MMT	Maximum Medical Treatment
MRC	Medical Research Council
MS	Member State
NHS	National Health Service
NHS R&D	National Health Service Research & Development
NICE	National Institute Clinical Excellence
NIH	National Institutes of Health (US)
NIHR	National Institute of Health Research (UK)
NTG	Normal Tension Glaucoma
OAG	Open Angle Glaucoma
OHT	Ocular Hypertension
OHTS	Ocular Hypertension Treatment Study
OR	Odds Ratio
PGA	Prostaglandin Analogue
PI	Principal Investigator
PIS	Participant Information Sheet
PPP	Preferred Practice Pattern
QA	Quality Assurance
QALY	Quality Adjusted Life Year
QC	Quality Control
QP	Qualified Person for release of trial drug
RCT	Randomised Controlled Trial
REC	Research Ethics Committee
SAE	Serious Adverse Event
SAP	Standard Automated Perimetry
SAR	Serious Adverse Reaction
SD	Standard Deviation
SDV	Source Document Verification
SEAGIG	South East Asian Glaucoma Interest Group
SITA	Swedish Interactive Thresholding Algorithm
SLT	Selective Laser Trabeculoplasty
SmPC	Summary of Product Characteristics
SOP	Standard Operating Procedure
SSA	Site Specific Assessment
SSAR	Suspected Serious Adverse Reaction
StAP	Statistical Analysis Plan
SUSAR	Suspected Unexpected Serious Adverse Reaction
TM	Trabecular Meshwork
TMG	Trial Management Group
TPC	‘Treat in Pursuit of Control' design
TSC	Trial Steering Committee
UCLP	University College London, Partners
UKGTS	UK Glaucoma Treatment Study
US	United States
VCDR	Vertical Cup Disc Ratio
VF	Visual Field
VFD	Visual Field Defect
VFL	Visual Field Loss

1 Trial personnel

Chief Investigator (CI)	**Mr Gus Gazzard**
	e-mail: gusgazzard@gmail.com
	tel: 020 7566 2087fax: [fax no.]
Research Manager	Dr Amanda Davis
	e-mail: amanda.davis@moorfields.nhs.uk
	tel: 020 7566 2823
Trial Optometrist	Mr Neil Nathwani
	e-mail: neil.nathwani@moorfields.nhs.uk
Co-Investigators	**Prof David Garway-Heath**
	e-mail: david.garway-heath@moorfields.nhs.uk
	**Mr Keith Barton**
	e-mail: keith@keithbarton.co.uk
	**Mr Richard Wormald**
	e-mail: r.wormald@ucl.ac.uk
	**Prof Stephen Morris** (UCL Health Economist)
	e-mail: steve.morris@ucl.ac.uk
	**Ms Rachael Hunter** (UCL Health Economist)
	e-mail: r.hunter@ucl.ac.uk
	**Prof Gary Rubin**
	e-mail: g.rubin@ucl.ac.uk
	**Dr Marta Buszewicz** (PRIMENT triallist)
	e-mail: m.buszewicz@ucl.ac.uk
	**Dr Gareth Ambler** (Lead Statistician)
	e-mail: gareth@stats.ucl.ac.uk
	**Dr Catey Bunce** (collaborating Statistician)
	e-mail: c.bunce@ucl.ac.uk
Sponsor's representative	**Maria Hassard**
	e-mail: Maria.Hassard@moorfields.nhs.uk
	tel: 020 7566 2059 fax: 020 7566 2059
Statistician	**Dr Gareth Ambler**

	e-mail: gareth@stats.ucl.ac.uk
	tel: 020 3447 2178
Clinical Trials Unit (CTU)	**PRIMENT (UCL)**
	Wendy Wang
	Research Design Service London/PRIMENT Clinical Trials Unit
	Department of Primary Care and Population Health
	UCL Medical School
	Royal Free Campus
	London NW3 2PF
	e-mail: yuepeng.wang@ucl.ac.uk
	tel: **0207 7940 500 ext. 36724**
Local PIs, Collaborating Centres	**Miss Sarah Wilson**
	e-mail: sarahj.wilson@belfasttrust.hscni.net
	**Mr David Broadway**
	e-mail: davidbroadway@waitrose.com
	**Mr Sheng Lim**
	e-mail: shenglim@gmail.com
	**Prof Rupert Bourne**
	e-mail: rb@rupertbourne.co.uk
	**Mr Timothy Manners**
	e-mail: Timothy.Manners@York.NHS.UK
Routine local laboratories	Not applicable
Central laboratories	Not applicable

Pharmacies: no specific trial pharmacy is involved as there is no IMP

Summary

Title: Health-Related Quality of Life in two treatment *pathways* for newly diagnosed open angle glaucoma and ocular hypertension: an unmasked, multi-centre, randomised controlled trial of initial selective laser trabeculoplasty versus conventional medical therapy.

Short title: LiGHT: Laser in Glaucoma & Ocular Hyper-Tension

Trial medication: *No IMPs in this study*

Phase of trial: *Not Applicable*

Objectives: To establish whether initial treatment with Selective Laser Trabeculoplasty (SLT) of patients with newly diagnosed open angle glaucoma (OAG) or ocular hypertension (OHT) is superior to current standard initial treatment with topical medication alone, in terms of: (a) better Health-Related Quality of Life (HRQL) at 3 years; (b) less cost; (c) equally good intra-ocular pressure (IOP) control with less need for topical medication and (d) better patient tolerance.

Outcomes: Quality Adjusted Life Years by EQ-5D health states, cost and cost-effectiveness, disease and treatment-related symptoms, visual function and objective measures of pathway efficiency.

Type of trial: Unmasked, multi-centre, pragmatic randomised controlled trial in newly diagnosed patients with open angle glaucoma and ocular hypertension.

Trial design and methods: This is an RCT with two treatment arms: 'initial SLT followed by conventional medical therapy as required' ('Laser-1st') and 'medical therapy without laser ('Medicine-1st'). It compares HRQL in the two arms at three years, while also examining the incremental cost and cost-effectiveness of Laser-1st versus Medicine-1st. A 'Treat in Pursuit of Control' design (TPC) compares two different routes (pathways) to a pre-defined target IOP. It is a pragmatic study that uses published guidelines to make the complex clinical treatment choices faced in managing glaucoma, standardised between treatment arms by use of computer treatment algorithms. A NICE-compliant evidence-based IOP Treatment Target [1] is set for each patient, according to the study treatment algorithms. They then proceed through stepped increments of treatment intensity (up to and including surgery) until a predetermined Target IOP is reached. Target IOP is reassessed in the light of objective clinical evidence of stability of glaucomatous optic neuropathy (GON) and visual function. HRQL and secondary outcomes are compared for patients in each pathway.

**Trial duration per participant:**	3 years
**Estimated total trial duration:**	5 years (with 6 months set-up and wind-up periods)
**Planned trial sites:**	Multi-site with 5 sites
**Total number of participants:**	718
**Main inclusion criteria:**	Diagnosis of open angle glaucoma (defined as an open drainage angle *and* reproducible glaucomatous visual field defects as tested by the SITA algorithm on the Humphrey Visual Field *or* glaucomatous optic neuropathy) or ocular hypertension (intra-ocular pressure above 21mmHg *and* requiring treatment as per NICE Guidelines) with a decision to treat made by a Consultant Glaucoma Specialist. Age over 18 years and able to provide informed consent.
**Statistical methodology and analysis:**	A single main analysis will be performed at the end of the trial based on all participants as randomised, irrespective of compliance with allocated treatment (Intention To Treat Analysis). The primary outcome will be compared between treatment arms using regression methods (analysis of covariance) that adjust for the randomisation factors: severity and centre. We will use mixed models to investigate how primary and secondary outcomes change over time to allow analysis of repeated outcome measurements data while taking into account correlation between measurements from the same patient. Patients will be lost to follow-up and our sample size assumes 15% of patients would not provide an evaluable 36 month EQ-5D. Missing data statistical modelling techniques will be used to make use of outcome assessments prior to 36 months and sensitivity analyses will be conducted to assess the appropriateness of the treatment estimates to these approaches. The unit of analysis for the primary outcome is the patient, with bilateral disease included as a covariate. Presentation of all findings will be in accord with the latest CONSORT statement.

2 Introduction

2.1 Background: the burden of the problem & its importance to the NHS

Glaucoma is a common, irreversible, optic neuropathy affecting the vision of predominantly older adults that slowly progresses over a period of years. It affects over 2% of those over 49 years, rising to 4% of white and 15% of black populations by the age of 80 [2]. In the UK glaucoma affects over half a million individuals, with over quarter a million over 65 [3]. It is a leading cause of visual morbidity, accounting for 12% of blind registrations [4] (despite fewer than 50% of the glaucoma-blind being registered). It is a significant cause of falls (increased risk of falls, odds ratio (OR) 3.71 [5]), road traffic accidents (OR 6.72 [5]) and loss of independence in the elderly in even mild asymptomatic disease [5;6]. Glaucoma significantly reduces quality of life, which is worse with more severe field loss [7-10].

OHT is a state of raised IOP without optic nerve damage which progresses to OAG in a some patients [11;12]. Around 1.2 million individuals have raised IOP in the UK [13]. Those with higher IOP, thinner central corneal thickness (CCT) and a family history of OAG are more at risk [14]. IOP reduction reduces the risk of conversion to OAG (from 9.5% to 4.4% over 5 years; hazard ratio 0.40) [12]. NICE recommends treating OHT in those most at risk of developing OAG [1].

The treatment of OAG/OHT and the resulting blindness impose significant costs. The total annual costs in Australia for 2005 were $1.9 billion, of which $355 million were health system costs [15]. Direct treatment costs in the UK were estimated at $1337 per patient per year in 1999 [16], up to 61% of which were for medications [17]. Both direct and indirect costs are higher for more severe disease (US$623 for mild to US$2511 for severe [18]) and thus more effective control of IOP early in the disease is likely to reduce later costs as well as improve vision-dependent HRQL. Rein has modelled the cost effectiveness of early medical treatment in the US system at $28-46,000 / QALY [19].

Studies that have attempted to estimate the relative costs of Argon Laser Trabeculoplasty (ALT) or SLT [15;17;20;21] have used either modelling or estimates of the treatment cost rather than direct cost assessment, and none have been performed in the NHS setting. In the US, Cantor, Katz et al compared the treatment costs of uncontrolled glaucoma treated with either further medications, SLT followed by further medications or surgery [20]. They used Markov modelling, with US cost assumptions based on Medicare fee schedules: the 5-year cumulative costs per patient were $6571, $4838 and $6363 in the medication, SLT and surgery arms, respectively. An Australian study modelled the cost benefit of any laser trabeculoplasty (LT) as primary treatment compared to conventional medical treatment followed by laser then surgery [15;21] and found a saving of $2.50 for every $1 spent on laser treatment, compared to initial medical therapy.

There are an estimated 11,054 new cases of definite glaucoma in people aged 40 to 70 per year in the UK [22;23] and according to recent NICE Guidance [23] a further 344,000

patients a year are referred to hospital as OHT or glaucoma-suspects, of whom a proportion require treatment. 2.3% of the over 40s in the UK receive treatment for OAG or OHT [24]. While limited existing data are very difficult to apply to the UK population, Cantor's data [20] would suggest annual savings to the NHS of £2.4m in direct treatment costs for new OAG patients alone from a Laser-1st paradigm. This rises to £16.8m per year if a conservative 20% of new OHT/glaucoma suspects require treatment. Australian data give far higher predictions. Were SLT to be extended to previously diagnosed patients, as is common practice in the US, cost savings would be up to 20 times higher. Indirect cost savings (e.g. reduced visual loss) are of course greater still.

2.2 Investigational medicinal product(s): Not applicable

2.3 Preclinical data: Not applicable

2.4 Clinical data: Not applicable

2.5 Rationale and risks/benefits

2.5.1 The condition and its treatment

Progressive visual loss can be halted or slowed at all stages of glaucoma. IOP is the only modifiable risk factor proven to alter the disease course and thus the associated morbidity. Laser, medicines and surgery can all successfully reduce IOP [25-29]. If medical treatment is selected, the installation of drops needs to be lifelong. Surgery, while effective, carries significant operative risks and is usually reserved for those who continue to lose vision despite other treatments. It also has a significant failure rate, often causes permanent ocular discomfort and rarely chronic pain [30;31].

Medical (eye-drop) therapies are widely used (1.2 million prescriptions per month in the UK; prevalence of topical treatment for OHT or OAG is 2.3% of all those > 40 years in 2003 [24]). They can be effective, but a significant minority require more than one type of drop with a third of patients in the UK using more than one drug (Allergan, on file). Drops are expensive with side effects that limit acceptability and impair HRQL[32-34;35]. Long-term topical medications are often associated with pain on instillation, multiple side effects (such as stinging, eye irritation, red eyes, hyper-pigmentation of eye-lid skin, iris colour changes, allergy to drug or preservative and accelerated cataract formation), poor patient compliance and multiple hospital visits [32-34;36-39]. Drop use requires regular monitoring and frequent adjustment: 22% of changes to drop regimes are due to adverse reactions [37]; 434 new patients with OAG still under follow-up at three years had a mean of 6.9 visits (Huntingdon, personal communication).

Complex treatment schedules and local side effects reduce compliance with treatment [40]. Reported non-compliance rates range from 24% to 80% depending on definition [38-42]: half of those started on glaucoma treatment had discontinued eye-drops by six months in one study [38]. Some of the most effective and widely used drugs – topical beta-blockers – are

associated with impaired exercise tolerance, airways obstruction, falls and excess mortality [32;43;44] due to respiratory and cardio-vascular side effects. Long-term drop use is also a very powerful risk factor for later surgical failure, due to conjunctival fibroblast activation by medications or preservatives [45-50].

LT is uncommonly performed in the UK, and NICE has identified a lack of evidence governing its use [1]. It involves the single, painless outpatient application of laser to the trabecular meshwork using a contact lens. There are two main forms of LT potentially used to treat glaucoma: Argon LT (ALT) and selective laser trabeculoplasty SLT. ('Selective' refers to the greater localisation of effects on target tissues and reduced thermal destruction with SLT).

The older ALT a uses a small laser spot size that requires very precise placement by a highly skilled operator, and causes greater tissue destruction than SLT. ALT carries significant risk of uncontrollable IOP-spikes and permanent trabecular meshwork damage (peripheral anterior synechiae) and can adversely influence later surgical success. SLT uses bursts of nanosecond pulses with a larger spot and is much easier to perform. SLT creates minimal coagulative damage [51-56] that generates a pressure lowering effect through increased macrophage activity and trabecular tissue remodelling, which lower IOP [57-59].

ALT as initial treatment is better than topical beta-blockers [60] at preventing visual field loss and it controlled IOP for 5 years in one third of patients with previously uncontrolled progressive disease [61]. SLT is at least as good at lowering IOP as ALT with fewer side effects [62-64]. It is safer and more effective in re-treatment [62;64;65] than ALT. The IOP-lowering effects of SLT are comparable to current first-line treatment, prostaglandin analogues [66;67]. SLT lowers IOP by 20% or more in c. 80% of patients and delays or prevents the need for glaucoma drops [66-69]. The effects are not permanent but SLT does not prejudice the effectiveness of later medical or surgical treatments.

2.5.2 *Why choose SLT as a first treatment for OAG/OHT?*

The recent NICE guidance on glaucoma [1] and the Cochrane Collaboration [70] have both clearly identified a need for robust randomised controlled trial (RCT) evidence of efficacy and cost effectiveness of LT. This study is an answer to those direct research recommendations.

Initial treatment with LT potentially offers a 'drop-free window' of several years, removes concerns about compliance and probably reduces the need for multiple drops even years later. Even when insufficient as sole therapy, LT reduces the intensity of subsequent medical treatment and possibly need for later surgery. More effective long-term IOP control in glaucoma leads to better visual outcomes and less blindness. Drop usage is itself associated with poorer HRQL in glaucoma patients [35]. A single outpatient treatment is likely to be more acceptable to patients than daily self-administration of eye-drops for many years.

SLT is likely to be more acceptable to patients, with 100% compliance from those attending for treatment, fewer hospital visits and fewer side effects than drop therapy alone. Although it is an existing technology proven to lower IOP [62;64;66;71] neither HRQL nor cost-effectiveness have been compared with patients started on drops. A Laser-1st [66-68] pathway allows a drop-free period and, later, lower-intensity treatment. This is likely to be associated with greater HRQL, improved patient acceptability and better treatment compliance with fewer patient visits due to treatment changes and adverse events compared to patients treated with Medicine-1st.

Economic modelling predicts that it will do this at much lower cost [15;17;20;21].

Uptake of LT by surgeons in the UK has so far been limited due to their past experiences with older ALT technology. SLT is delivered in an outpatient setting using topical anaesthesia, is quick and pain-free. It is simple and safe to deliver by anyone competent in gonioscopy and has a wide safety margin. Delivery of SLT by optometrists would allow wide-scale implementation with limited additional training and no workforce expansion, thereby further reducing costs (pilot study underway at Moorfields) and in keeping with NICE and Department of Health proposals for changing models of glaucoma care. Around one quarter of glaucoma medication changes are due to adverse reactions [37]. Use of SLT as first-line treatment would facilitate community-based glaucoma monitoring by non-prescribing optometrists because it would lower adverse drug reaction rates (from around 10% patients per year [37]).

Widespread uptake of SLT has the potential to substantially improve HRQL for many patients and produce substantial cost savings to the NHS (from lower drug costs and reduced side effects, fewer hospital visits and less surgery and indirect savings from care-costs for fewer visual impaired patients).

Most studies of medical therapy are funded by the pharmaceutical industry, with far higher rates of compliance than seen in clinical practice, so may over-estimate the success of medical treatment.

2.5.3 *Benefits to society and Generalisability*

This study answers direct research recommendations of both recent NICE Guidance on Glaucoma [1] and the Cochrane Collaboration [70]. More effective long-term IOP control in glaucoma leads to better visual outcomes [25-29], less blindness and, it is presumed, better HRQL. Even similar long-term control of IOP but a reduced need for medications may produce an improved HRQL (fewer visits to obtain medications and fewer side effects) and lowered costs (fewer visits [72] for treatment changes and lower drug costs). Reduced drug requirements, even for those taking drops, improves compliance through simpler treatment regimes and fewer side effects [38;40].

Widespread uptake of SLT could potentially produce substantial cost savings to the NHS from lower drug costs, fewer hospital visits and postponed / less surgery. Approximately half a million patients with glaucoma and a similar number with OHT are potentially eligible for this treatment. More indirect savings would be from the reduced patient costs from

fewer visits, lowered costs of care for the fewer visually impaired patients and reduced costs of caring for the complications of poor vision such as falls [5] and hip fractures etc.

SLT is simple and safe to deliver by anyone competent in gonioscopy and optometrist as opposed to ophthalmologist delivery of SLT would allow wide-scale implementation with limited additional training and no workforce expansion. SLT could be readily undertaken in all non-specialist general ophthalmic units and community-based clinics, as well as specialist centres. Approximately eighty SLT lasers are in use in the UK already, mostly in private hospitals and specialist units. The technology is the current laser standard but the evidence base is inadequate to justify roll-out to all NHS units without this trial.

2.6 Assessment and management of risk

There are no safety concerns for either pathway that extend beyond normal clinical practice as outlined in national and international treatment guidelines (NICE [1] and European Glaucoma Society (EGS) guidance [73]). Exposure to medication and laser entails risk, but as in standard medical practice these are mitigated through specific enquiry into existing contra-indications and careful assessment for possible side effects. The potential 4 week interval between identification and treatment (the maximum permissiable delay, if requested by the participant) theoretically carries risk, but glaucoma is a chronic disease with a time-course of many years and this puts patients at no significant additional risk from progression of GON. To minimise the hypothetica*l* risk of a rare IOP-related vascular occlusion while treatment is pending, all subjects with untreated IOP >40mmHg will be reviewed within 72 hours of identification. Patients with very severe disease who might be at a small but significant risk of deterioration from treatment delay are not included in this study.

Adverse events (AEs) will be managed as per our standard CTU SOPs (see section 9.4). Any unexpected AE will reported by either the Yellow Scheme (for medicinal products) or via the MHRA online reporting scheme (for devices, i.e. laser) and through internal hospital critical incident reporting if applicable.

Unblinding is not an issue in this trial as it is unblinded.

The study will be conducted according to principles of good clinical practice provided by MRC Good Clinical Practice Guidelines and Research Governance Guidelines.

3 Objectives

3.1 Hypotheses

That for patients with ocular hypertension (OHT) or open angle glaucoma (OAG) lowering IOP with SLT as the primary treatment (‘Laser-1st’) leads to a better health-related quality of life than for those started on IOP-lowering drops as their primary treatment (‘Medicine-1st’) and that this is associated with: reduced costs and improved tolerability of treatment.

3.2 Primary:

To determine whether, in a pragmatic study that mirrors the realities of clinical decision-making, Laser-1st delivers a better HRQL at 3 years than does Medicine-1st in the management of patients with OAG and OHT.

3.3 Secondary:

To determine whether a Laser-1st treatment pathway:

a) Costs less than the conventional treatment pathway of Medicine-1st,

b) Achieves the desired level of IOP with less intensive treatment over the course of the study,

c) Leads to equivalent levels of visual function after 3 years,

d) Is better tolerated by patients.

4 Trial design

4.1 Overall design

This non-commercial study compares HRQL (EQ-5D [74]) at 3 years for two treatment pathways that are identical except for the use of SLT as the initial step in the Laser-1st arm, after which patients who fail to meet their IOP Target enter the medical treatment algorithm. Criteria for re-intervention and further treatment are identical in each arm. It is an unmasked, multi-centre, randomised controlled trial.

The 'Treat in Pursuit of Control' design (TPC) compares two different routes (pathways) to a pre-defined target IOP. Individualised IOP Targets and triggers for escalation of treatment are stringently and clearly defined and set through clinical algorithms that attempt to capture the complexities of clinical practice. To define 'Failure to Meet Target' we take into account the complexities of measurement error and diurnal fluctuation in IOP measurement in assessing whether deviations from Target are clinically significant. (See flow-charts in Appendices). With each Failure to Meet Target or worsening of glaucoma ('progression') an additional increase in intensity of treatment is made, up to and including surgery. If there is a Failure to Meet Target either without progression or on maximum treatment then the Target IOP will be reassessed in the light of these new data.

The conventional approach makes comparisons of IOP between fixed treatments to look at treatment *efficacy*. Direct efficacy comparisons (laser-with-laser or laser-with-medication) have been made [62;64;66;67;71;75]. Although important to help guide treatment decisions, they are too constrained to include the actual range of management options. They cannot adequately represent clinical practice and so cannot form the basis for realistic assessments of HRQL or cost.

The 'Treat in Pursuit of Control' (TPC) design mirrors the realities of clinical decision making, where choices are made between all available treatments to reach a desired IOP. We are looking at the difference treatment *effectiveness* makes to HRQL and health costs. Although it is a more difficult design, it more closely reflects real-life clinical care.

For a flow-chart of overall trial design see section 26.

4.2 Recruitment of participants

Subjects not previously treated for OAG / OHT will be recruited from 5 collaborating specialist glaucoma clinics at large ophthalmic centres in the UK (participation agreed) by the Trial Fellow for that centre. Individuals will be invited to participate when treatment is first offered, frequently but not always, the first visit.

4.3 Proposed duration of the intervention

The RCT has a total duration of 6 years (6 months set-up from successful ethics application; Internal Pilot of 9 months included in total recruitment 2 years; follow-up 3 years; analysis 6 months). The 'intervention' is the whole treatment pathway and persists for the duration of the study. Any shorter follow-up might fail to capture the full period of the SLT treatment / retreatment cycle in the majority of patients and could provide misleading treatment costs.

4.4 Justification of unmasked study design

This randomised controlled study will be unmasked to the initial treatment. This is necessary to remove the distorting effect of placebo or sham laser both on our primary outcome measure, HRQL, and secondary outcomes including ocular symptoms. An important benefit of LT is a "drop-free treatment window", lasting years in some patients. Any placebo arm, as required by a masked study, would negate this important potential benefit and would expose patients to preservatives, the burden of regular treatment and the need to refill repeat prescriptions.

We also aim to match the real-world setting in which treatment decisions take place and to capture fully the effects on HRQL of treatment. One of the major concerns with medical treatment is poor compliance. Patients' knowledge of prior treatment may influence medication-taking behaviour: initial treatment with even 'sham' laser might affect patients' later compliance.

An unmasked study design has been accepted where the use of placebo or masking itself might affect the outcome. In this case concern about possible confounding effects of placebo treatment and altered compliance from sham laser require an unmasked design. Steps to minimise bias are outlined below.

4.5 Methods to protect against other sources of bias

There is a tension in this study between minimising potential bias from the unmasked clinicians (e.g. by rigidly codifying all treatment decisions) and maximising its generalisability and pragmatic structure (e.g. by allowing free rein in decision-making). Although the subjects and observers are of necessity unmasked, treatment decisions are masked. Assignment of subjects to a severity-category, setting of the Treatment Target and its reassessment, treatment escalation decisions (stepped incremental increases in response to carefully defined triggers) and determination of follow-up interval are all determined according to structured protocols using computerised evidence-based decision algorithm. This will be written by the existing programmer for the OpenEyes electronic patient record glaucoma-module (funding by Moorfields Special Trustees agreed).

The treatment pathways and follow-up schedules in this study are more structured than in clinical reality. This will apply equally to each arm and should not influence inter-group comparisons. Disproportionate recruitment of different disease severities into one treatment arm is a potential source of bias. To stratify randomisation by the 4 severity categories of the Canadian Target IOP Workshop [76] would present significant logistical problems, risks sparse allocation categories and could jeopardize concealment. We shall therefore dichotomise patients and stratify based on diagnosis (the OHT / OAG groups are likely to require the greatest differences in treatment intensity) as well as centre.

5 Selection of Subjects

5.1 Inclusion criteria

We have used the NICE recommended thresholds for initiating treatment [1], with stringent diagnostic definitions of disease (OAG or OHT) for entry into the study.

Uniocular patients are eligible.

5.1.1 Diagnosis of OAG

Primary Open Angle Glaucoma is defined as an open drainage angle [no irido-trabecular contact on non-indentation gonioscopy in primary position, trabecular meshwork visible over 360 degrees], with no secondary causes (such as trauma),

(1) **and** reproducible glaucomatous visual field (VF) defects as tested by the SITA algorithm on the Humphrey Visual Field Analyser (HVF) (i.e. reproducible defect, in at least, of two or more contiguous points with P < 0.01 loss or greater, or three or more contiguous points with P<0.05 loss or greater, or abnormal Glaucoma Hemifield Test, GHT);

(2) **or** GON with localised absence of the neuro-retinal rim or, cup disc ratio of 0.7 or more, or asymmetry of cup disc ratio of 0.2 or more in similar sized eyes / optic discs.

and deemed to require treatment in the opinion of the treating (fellowship-trained) glaucoma specialist.

Subjects with pseudo-exfoliation are eligible (as for the EMGT study [77]).

Subjects with GON and IOP in the normal range are therefore eligible. 'Phasing' (diurnal IOP pressure measurements) will be performed at the discretion of the treating clinician and if performed the maximum IOP recorded will be used as that day's measurement.

5.1.2 Diagnosis of OHT

OHT with IOP above 21mmHg and requiring treatment as per NICE Guidelines [1]. NICE OHT guidelines treat 4 categories of OHT on the basis of central corneal thickness (CCT) and age (the rest are monitored for 3-5 years).

Participants are required to have *either* OAG *or* OHT *newly requiring treatment* and:

1. A decision to treat has been made by a Consultant Glaucoma Specialist.
2. Age over 18 years and able to provide informed consent.
3. Able to complete quality of life, disease-specific symptom and cost questionnaires in English (physical help with completion and assistance with reading will be permitted as long as an interpreter is not required).
4. An ability to perform a visual field test in the study eye(s) with <15% false positives.

5.2 Exclusion criteria

This is a pragmatic study aiming to match clinical practice as far as possible and to be as generalisable as possible. We have therefore kept the exclusion criteria to a minimum.

1. Advanced glaucoma in the potentially eligible eye as determined by EMGT criteria [77]: visual field loss mean deviation worse than -12dB in the better or -15dB in the worse eye.
2. Secondary glaucoma (e.g. pigment dispersion syndrome, rubeosis, trauma etc) or any angle closure.
3. Any contra-indication to selective laser trabeculoplasty (e.g. unable to sit at the laser-mounted slit-lamp; past history of or active uveitis, neovascular glaucoma, inadequate visualisation of TM etc).
4. Unable to use topical medical therapy due to e.g. physical infirmity *and* a lack of carers able to administer daily eye-drops.
5. Previous treatment for OAG or OHT.
6. Congenital or early childhood glaucoma
7. Visually significant cataract in symptomatic patients who want cataract surgery. Patients with lens opacity who are happy with their current acuity may be enrolled.

8. Under current, active treatment for another ophthalmic condition in the Hospital Eye Service. (This applies to *both* eyes, even if one is not in the trial, as the fellow eye might determine the patient's visit frequency).
9. Any history of retinal ischaemia, macular oedema or diabetic retinopathy.
10. Age-related macular degeneration with neovascularisation in either eye *or* geographic atrophy *and* VA worse than 6/36 in a study eye.
11. Visual acuity worse than 6/36 in a study eye. *Non-progressive* visual loss better than 6/36 due to any comorbidity is permitted provided that it does not affect response to treatment or later surgical choices and is not under active follow-up (e.g. an old, isolated retinal scar no longer under review, amblyopia).
12. Any previous intra-ocular surgery, except uncomplicated phaco-emulsification at least one year before. (This applies to *both* eyes, even if one is not in the trial, as it may affect the required treatment intensity for any glaucoma in the fellow eye).
13. Current pregnancy or intention to become pregnant within the duration of the trial. [Unanticipated pregnancy will lead to the normal limitation of the choice of medication available but not cause the participant to leave the trial until such as a time as a choice between surgery or SLT arises (when it would be unethical to withhold SLT and progress to surgery without anti-metabolites).]
14. Medically unfit for completion of the trial – e.g. suffering from a terminal illness or too unwell to be able to attend hospital clinic visits.
15. Recent involvement in another interventional research study (within 3 months).

5.2.1 There are no exclusions based upon concurrent medical treatments

5.3 Concomitant medication

There are no limitations on concurrent medical therapies for non-ophthalmic indications.

Acetazolamide ('Diamox') will be permitted as a temporary (up to 1 month) treatment only while waiting for surgery to be performed, if the eye pressure is dangerously high despite medical treatment.

Pilocarpine will not be permitted as treatment for glaucoma in this study

6 Recruitment

Subjects not previously treated for OAG / OHT will be recruited from 5 collaborating specialist glaucoma clinics at large ophthalmic centres in the UK by the local Trial optometrist or PI for that centre. Individuals will be invited to participate when treatment is first offered, frequently but not always, the first visit.

Patients with OHT who have been monitored for some time but only reach trial treatment criteria during the recruitment period (ie conversion to glaucoma or IOP rises) will be eligible.

Patients with OHT or OAG that was previously treated but treatment was discontinued for any reason will not be eligible as their past experience might affect future reported quality of life results.

Patient recruitment at any site will only be done when the trial has Documented REC, Regulatory and Local Trust R&D approval, a signed site agreement and the site has sent back to the TC the PI self-monitoring template and been initiated by the Sponsor.

6.1 Methods used to ensure recruitment to the study

Every patient attending for the first treatment of OAG / OHT will be assessed for eligibility before treatment and, if eligible, informed of the study by the local Trial Coordinator (backed up by written information), with an interpreter where required. To maximise potential coverage of all eligible patients a trial staff member will be available daily to attend clinics and counsel potential subjects. Local trial staff will screen all new referrals (by letter or electronic patient record) and flag those possibly eligible with reminders for the clinic staff. Regular education of clinical staff and clinic-wide information posters for staff and patients will raise awareness of the study and remind clinicians of the opportunity for recruitment. The pragmatic trial structure without burdensome additional visits or tests beyond standard care should maximise acceptability – as supported by a recent anonymous survey of new patients.

6.2 Recruiting centres & new patient numbers

6.2.1 Poll of new glaucoma patients at Moorfields

Consecutive new patients seen in the Moorfields Glaucoma Service were informed of the study design and asked (anonymously) "If it were available to you today would you consider being a part of the study described?". 70% expressed a willingness to participate, identical to the 70% *actual* acceptance rate for the *much* more intensive placebo-controlled multi-centre RCT 'UKGTS'.

6.2.2 Rates of medical/laser interventions for new patients

Review of the electronic records of all new referrals with possible glaucoma to one participating centre in 2009 (Huntingdon) showed that 17% of 435 new patients were started on treatment for OHT / OAG

6.2.3 Recruiting centres & new patient numbers

Moorfields received over 8,000 new referrals for glaucoma in 2009/10, of which 4685 were seen at the two local recruiting sites (1231 SGH + 3454 City Rd). Four specialist glaucoma

centres have agreed to participate (Huntingdon, St Thomas' Hospital, Norwich Hospital, and Belfast) all of whom have experience of RCTs and SLT. Approximate annual rates of new patient referral to these clinics are, respectively, 440, 1000, 1000 and 500 per year, with very comparable rates of newly diagnosed OHT/OAG requiring treatment (c.20%). If 17% of these require treatment and 70% of them were willing to participate, as our figures suggest, then of the 1296 patients requiring treatment, 908 would be potentially willing per year. A small proportion (<5%) will be ineligible due to co-pathology (e.g. severe AMD, diabetic retinopathy [78-81]) leaving 862 per year, i.e. 1724 over the two years of recruitment.

To recruit 718 in 2 years we need to recruit an average 30 patients per month, from all 5 sites. This represents 27% of those eligible and 40% of those provisionally willing to participate in research. We assume an attrition rate of 15% at 3 years, based on our UKGTS experience and since this is a pragmatic trial without additional visits or examinations.

Moorfields receives a further 3000 new referrals per year at additional sites in London that will also be considered for inclusion. These patients would experience longer journeys for follow-up so they have not been included in the projected recruitment rates but would yield an additional 339 eligible and willing patients *per year* (total 678) to whom we will offer participation. Projected recruitment is shown in detail on the Gantt chart in the Appendices.

6.3 Internal Pilot Study

We will conduct a 9 month Internal Pilot at Moorfields Eye Hospital (the Central Trial Site and largest recruiting site) to refine data handling, randomisation and treatment algorithms. It will ensure that recruitment rates are adequate and patient acceptance of randomisation is acceptable, before roll-out to other sites. Data collected will include: number of eligible patients approached, proportion entering the trial and recruitment rates. If recruitment at 9 months is <70% of expected (<60) we will call a TSC meeting to discuss recruitment strategies and whether we should continue the Trial, but recruitment below 50% would lead to the Trial being terminated. Predictions are based on: electronic patient records of proportions needing treatment; surveys of new patients and experience of recruitment rates into the UKGTS trial.

6.3.1 Strategic plan for unanticipated low-recruitment:

We conservatively included only the two largest Moorfields sites (City Rd & St George's) in our recruitment calculations. Of 10 other sites at least three will have on-site lasers and all receive new glaucoma patients. These 10 are not included in recruitment projections, but are a safe-guard in the event of low recruitment. Our collaborating centres include 3 of the most successful recruiters into UKGTS and EAGLE trials, and have systems to screen routinely all new patients for RCT eligibility.

7 Study procedures and schedule of assessments

7.1 Recruitment and informed consent procedure

Eligible patients will be identified by a member of the Trial team. Patients will be approached and introduced to the aims, methods, anticipated benefits and potential hazards of the study and eventually invited to participate.

Introducing the patients to the study and inviting them to participate will be done either by face to face discussions with trial team members or by the use audiovisual material (video); the video will convey the same information as the face to face discussions with the trial team members, but delivered by the consultant Ophthalmologist leading the trial (video content/script is shown in Appendix 14). The use of the video in the recruitment process will maximise the time efficiency of the recruiters, as often more than one patients have to be approached simultaneously[†].

All patients will be given a Patient Information Leaflet (PIL) and a copy of the Informed Consent. All patients will be given the opportunity to have a face to face discussion with the trial team member and to ask questions. The Investigator or designee will explain the patients are under no obligation to enter the trial and that they can withdraw at any time during the trial, without having to give a reason.

“Ample time” will be given for consideration by the patient before taking part; private face to face discussions with trial team members (with family members present where desired by the potential participant) will be followed by at least a day to reflect and discuss with other family members and a further opportunity to ask questions. The Investigator or designee will record when the PIL has been given to the patient.

All staff taking consent will have to have signed the Protocol Training Log, see Section 22. After the invitation to participate, written informed consent will be obtained by either the GCP-trained local trial ophthalmologist (PI) or local trial optometrist who has been delegated this duty by the CI/PI on the delegation log.

Consent will be obtained with the support of extensive clearly written information (in English) that has been reviewed and approved by our patient-led Trial Lay Advisory Group.

Vulnerable groups who would have difficulty in giving informed consent will not form part of this study.

It is the responsibility of the Investigator, or a person delegated by the Investigator to obtain written informed consent from each subject prior to participation in the trial, following adequate explanation of the aims, methods, anticipated benefits and potential hazards of the study.

[†] The use of the audiovisual material for recruiting participants may be the subject of a separate randomised controlled trial in the future, for which separate Ethical approval will be sought

A copy of the signed Informed Consent will be given to the participant. The original signed form will be retained at the study site.

If new safety information results in significant changes in the risk/benefit assessment, the consent form should be reviewed and updated if necessary. All subjects, including those already being treated, should be informed of the new information, giving a copy of the revised form and give their consent to continue in the study.

No study treatments are experimental - all are current clinical practice offered in specialist ophthalmic units.

7.2 Randomisation and allocation of participants to trial groups

Randomisation will be carried out according to a trial specific SOP. Online randomisation (with blocking with random block sizes) will be used to randomise at the level the patient and be stratified by diagnosis (OHT/OAG) and treatment centre as stratification covariates. The primary analysis will adjust for the stratification factors used in randomisation. Participants will randomised to one of the two study groups in equal proportion using a web-based randomisation service provided by a specialist company to achieve full allocation concealment (www.sealedenvelope.co.uk), available 24/7. 'SealedEnvelope' will also hold the randomisation list. A backup telephone service will be available.

7.3 Emergency un-blinding

This is not applicable as this trial is unblinded.

7.4 Screening assessments

Screening assessments will usually take place at the patient's first visit when the diagnosis of OAG or OHT requiring treatment is confirmed. All screening procedures would be done as part of routine care and include a history with routine clinical assessment: a slit-lamp examination (including intra-ocular pressure check, gonioscopy and optic nerve assessment), automated visual field test and scanning laser ophthalmoscope disc imaging (HRT).

7.5 Baseline assessments

The baseline assessments will be done once the patient has been entered into the trial and before their first treatment. These will be the same as the screening assessment (slit-lamp examination, automated visual field test and HRT disc imaging) with the addition of self-completed baseline questionnaires (EQ5-D [82], Glaucoma Utility Index [83] (GUI), Glaucoma Symptom Scale [84] (GSS) and Glaucoma Quality of Life - 15 [8] (GQL-15; a visual *function* measure).

7.6 Treatment procedures

7.6.1 Standardisation of Interventions & Incremental Escalation of Treatment

In practice, the interventions offered tend to vary widely between clinicians. To retain the widest possible generalisability we have used NICE recommended thresholds for *initiating* treatment [1] (inclusion criteria). To minimise bias for *escalating* treatment we use standardised criteria for any additional intervention according to a protocol following international guidelines (European Glaucoma Society [73], American Academy of Ophthalmology Preferred Practice Pattern [85], SEAGIG [86]). See Appendices 9 & 10. All interventions and examinations will be conducted according to a manual of standard operating protocols (SOP). The treatment pathways are identical *except* for the use of SLT as the initial step in the Laser-1st arm, after which patients who fail to meet their IOP Target enter the medical treatment algorithm.

7.6.2 Trial arm 1 : "Laser-1st" Pathway

SLT will be delivered to 360º of the trabecular meshwork with one 180° re-treatment as the first escalation of treatment if required. Which model of SLT laser may be used is not restricted, the wavelength and spot size are the same. To ensure quality control of SLT delivery and minimise variation between surgeons, standardisation will be achieved by a stringent protocol defining laser settings and technique including the range of acceptable powers (see Appendix 5a, section 30.1). All treating surgeons are already experienced in delivering SLT. Nonetheless, they will be given training before recruitment and at least one laser treatment directly observed by the CI. *After two SLT treatments the Laser-1st Pathway embarks on medical treatment and follows the Medicine-1st algorithms (below).*

A copy of the device product characteristics and the CE-mark certificates for the lasers to be used in this study is submitted with the Ethics application and will be available online (https://www.dropbox.com/login?cont=https%3A//www.dropbox.com/link/17.QXngvCHUyX%3Fk%3DHgPAlcOrdgLrvmh_7Jme20eNz_rn6Cdg%26eh%3D1b2214f).

Significant complications of laser treatment if they occurred would prevent a second treatment with SLT (e.g. corneal oedema, intra-ocular haemorrhage, severe uveitis, IOP spike greater than 15mmHg, peripheral anterior synechiae). Other new medical conditions (such as a new history of uveitis or rubeosis) would prevent repeat SLT.

7.6.3 Trial arm 2: "Medicine-1st" Pathway

Medical treatment of glaucoma involves several distinct steps that require standardisation: choice of drugs; number of agents permitted; rules for switching between or adding drugs. International best practice guidelines [73;85;86] advocate changing medication if the target is not reached, with the addition or switching of medication (based on the magnitude of initial response). Surgery is offered once maximum treatment intensity is reached: "Maximum

Medical Therapy (MMT)". This varies between patients, but requires definition to minimise inter-surgeon variation.

7.6.3.1 Maximum Medical Therapy – MMT

MMT is the most intensive combination of drops a given individual can reasonably, reliably and safely use. MMT varies between patients depending on co-morbidities, side effects and patient-specific compliance factors. While there is surgeon variation in attitudes to poly-pharmacy it is widely accepted that additional medications result in lower percentage reductions in IOP. Evidence shows there are profound reductions in compliance with complex dosage schedules. NICE Guidance [1] recommends offering surgery after only two drugs have failed to control IOP. We shall limit treatment with multiple different medications and define MMT in terms of the maximum number of drugs (3) and dosages per day (5 drops). MMT will often be less, due to drug intolerance, contra-indications and patient factors.

7.6.3.2 Choice of Agent:

No mainstream medications are prohibited but drugs classes for 1st, 2nd, or 3rd line treatment are defined as per NICE [1] and European Glaucoma Society (EGS) guidance [73]: 1st line: Prostaglandin analogue (PGA), 2nd line: Beta blocker (once in the morning or in a PGA combination), 3rd or 4th line: Topical carbonic anhydrase inhibitor (CAI) or alpha-agonist. Systemic CAIs are only permitted as a temporising measure while awaiting surgery and will not influence treatment escalation. Pilocarpine is not an accepted medication for OAG. Novel medications / preparations will likely be available during the study. To maximise generalisability we shall permit new medications, after discussion at the Trial Steering Committee of the available published evidence.

7.6.3.3 Add or Switch?

The Incremental Escalation of Treatment protocol defines stepwise increases in treatment. Patients may be *switched* if the pre- and post- treatment IOP difference is no greater than measurement error. If there is a greater reduction but the eye is still not at Target then the next medication may be *added*. Progression of GON when at Target will also trigger a stepwise increase of treatment *and* a lowering of the target (as above).

7.7 Computerised decision algorithm

Complex interventional decisions, such as Target IOP-setting and changing, will made with point-of-care decision support software which has replaced the Trial Reading Centre of the original application. However, there will be telephone back-up from the Trial Management Office in the event of IT failure. The few anticipated decisions concerning treatment escalations required on the basis of disc or VF progression will undergo masked review by the Trial Management team to ensure strict agreement with the trial progression criteria. Masked data (VF or HRT) will be presented to the panel by the Trial Fellow within 5 days of the latest patient visit. The treatment escalation decision will not be delayed for confirmation except in the case of possible surgery.

All algorithm decisions to escalate treatment will be reviewed by the treating clinician to exclude other causes (such as retinal vein occlusions etc).

7.8 Setting Individualised Patient Treatment-Targets

(See flow-charts for details, section 33, Appendix 8).

As with usual practice, the decision to start treatment is made by the clinician in discussion with the patient on the basis of perceived risk to the patient's vision, if left untreated. Once the decision to treat is made, in accordance with NICE thresholds for treatment, a 'Treatment Target IOP' ('Target') is set. This is the "highest IOP level that is expected to prevent further glaucomatous damage or that can slow disease progression to a minimum" [73] or "a range of IOP that is adequate to stop progressive pressure-induced injury" [85]. It is related to: untreated IOP, severity of glaucoma, age & life expectancy, rate of progression during follow-up, other risk factors (e.g. family history; pseudo-exfoliation; myopia) and risk to vision (eyes with central visual field defects may require lower targets than those with more peripheral loss) [76;87-89]. Data from large clinical trials of IOP reduction have attained variable risk reduction with different levels of IOP reduction (Ocular Hypertension Treatment Study (OHTS) [12] 20%; Early Manifest Glaucoma Treatment study (EMGT) [90] 25%; Collaborative Normal Tension Glaucoma Study (CNTGS) [25] 30%; Collaborative Initial Glaucoma Treatment Study (CIGTS) [27] >35%; Advanced Glaucoma Intervention Study (AGIS) [28] <18mmHg).

We are using the Canadian Target IOP Workshop's algorithm [76]: it has clear criteria and a robust evidence base drawing on multiple large RCTs [11;27-29;90;91]. We have added a definition of central visual field loss lacking from the original, as per Mills *et al* [92]. The Target IOP will be either an absolute reduction to below a specified level, or a percentage reduction from baseline, whichever is lower (see section 33, Appendix 8). Greater reductions are required for greater disease severity as defined by Canadian Glaucoma Study criteria [93] (see section 28 Appendix 3). In accordance with NICE [1], "Glaucoma Suspects" in whom OHT is present but a definite diagnosis of GON cannot be either made or ruled out will be treated according to the OHT category. Surgical risks increase with low Targets. The lowest permitted Target is 8mmHg for POAG and 18mmHg for OHT. The 'Target' is objectively defined to avoid bias from unmasked treating ophthalmologists. Targets will be set and adjusted by application of the computerised decision algorithm. The Target is eye-specific.

We recognise that CCT has an effect on IOP measurement and risk of progression. However the true magnitude of this interaction is unknown because of complex non-linear interactions between CCT, 'true' IOP and corneal material properties, the latter two being unknown variables. There is as yet no evidence to include CCT in 'Target IOP' algorithms in a quantitative manner We have therefore not included the measurement of CCT in our algorithm for setting Target IOP. IOP measurement error will apply in both treatment arms and will be approximately consistent pre- and post- treatment; inclusion of a *percentage* reduction will largely mitigate this. The possible direct relationship of CCT to risk of GON progression is unproven. Additional potential risk factors such as myopia, family history

etc. are not included in this algorithm as data on the effect size of these risk factors on progression rates are weak.

The decision to *treat* is made by the clinician, per eye; the algorithm helps set the IOP Target, per eye.

7.9 Treatment Changes

Treatment will be escalated under the following circumstances:

- "Strong Evidence" of progression (as defined below) irrespective of IOP.
- IOP above Target by more than a certain threshold at a single visit (irrespective of evidence for progression)
- IOP above Target by less than threshold plus "Less Strong Evidence" for progression. If the IOP is above Target by less than threshold with no evidence for progression, then the 'Treatment Target IOP' will be re-evaluated. More detail of the indications for treatment escalation and 'Treatment Target IOP' re-evaluation, to deal with specific clinical scenarios, is given in Appendices 6 & 8 (sections 31, 34).

7.9.1 Definition of 'Failure to Meet Target'

(See flow-charts in Appendices 6 & 9, sections 31, 34.1, 34.2 for details).

Diurnal fluctuation and measurement error both lead to variation in measured IOP. We shall minimise the former by performing follow-up tests at a similar time of day. We shall minimise the latter through regular instrument calibration, careful observer training and robust mechanisms to demonstrate good inter-observer agreement. Kotecha *et al* have shown that inter-visit variation may nonetheless be as much as +/-4mmHg. To prevent an inappropriate escalation to more intensive treatment it is therefore important to repeat measurements that deviate only slightly from Target. Criteria for failure to meet target and to reassess Target follow those of the CGS [93], with additional steps where not specified in the CGS. The specific deviations in IOP that trigger treatment changes are described in Appendix 6 (section 31). Failure to meet Target can be due to poor compliance as well as a lack of drug efficacy. As in normal practice, compliance will be discussed and patients counselled at each visit, using validated 'Ask-Tell-Ask' techniques [94-96]. Patients will be given standard written information from a patient support charity (IGA), face-to-face instruction in drop administration and the offer of further nurse-led support.

Where poor compliance is thought to be the contributing factor then education with written information and repeated face-to-face instruction in drop administration will be given. If the decision is made to educate rather than escalate a patient who is not at target then the reason for an algorithm over-ride must be recorded ('non-compliance') and the patient recalled after 8 weeks for a repeat IOP-check visit.

7.9.2 Process for Treatment-Target Reassessment

(See flow-charts in Appendices 6 & 9, sections 31, 34.1, 34.2 for details).

Accurately predicting a safe level of IOP for a given patient is inherently difficult before individual data on rates of nerve damage are available. International Treatment Guidelines recommend that IOP Targets are revised as further data are collected [73;85;86]. That is, guidelines derived from population data are refined for the individual, based on data from that individual.

7.9.3 Treatment escalation to glaucoma surgery

More stringent criteria are applied before undergoing surgery than laser or medical treatment. This reflects the greater risk to vision from surgical complications. Strong evidence of progression +/- failure to meet Target is usually required in all but the most severe disease. However, extreme elevations of IOP may require surgery without progression, with lower thresholds in more damaged eyes. We define '*Maximal IOP'* as that above which surgery *may* be offered without progression (but need not be, at the discretion of the treating surgeon): OHT 35mmHg; Mild glaucoma: 24mmHg; Moderate and Severe glaucoma 21mmHg. Any patient who is at or above Maximal IOP must have their case reviewed (in person or remotely) by the PI for this decision to be made.

In accordance with patient-centred care the decision to operate is always a collaboration between clinician and patient.

When an intra-ocular pressure lowering surgical intervention is indicated, cataract surgery will be permitted (in the presence of cataract, i.e. not clear lens extraction) when this is the consultant's usual practice.

7.10 Detection of Progressive Glaucoma Damage

Detection of progressive nerve damage is a trigger to increasing treatment intensity. We follow NICE recommendations on follow-up intervals [1], with Humphrey Visual Field (HVF) tests and Heidelberg Retina Tomography (HRT) digital optic disc imaging at trial entry and each visit.

7.10.1 Visual Field Progression

Worsening of visual field loss (VFL) will be defined as 'Likely' or 'Possible', in the absence of any identifiable retinal or neurological cause. The 'minimum dataset' to determine VF progression is 2 reliable baseline VF measurements followed by 3 follow-up VF and will take 1 to 2 years from enrolment to confirm. '*Likely VF Progression'* is 3 points or more on the HVF Glaucoma Progression Analysis (GPA) software at <0.05 probability for change on 3 consecutive occasions. '*Possible VF Progression'* is 3 points or more on HVF GPA software at p <0.05 probability for change on 2 consecutive occasions. VF series will be

independently assessed for progression using the automated algorithm software at each visit.

These standard GPA criteria weight central and peripheral field locations equally whereas clinical practice is to use a lower threshold for central field loss. Thus if any of the 4 para-central point's shows a triangle then the algorithm will recommend treatment escalation when 2 GPA triangles (rather than 3) indicate deterioration on 2 or more consecutive occasions (rather than 3), with the second triangle being any point that is contiguous with the affected central one (thus including the other central 3 but also the neighbouring less central points).

Any treatment escalation triggered by worsening visual field loss will require senior clinician verification that there is no retinal or neurological cause.

7.10.2 Optic Disc Progression

Chauhan showed sequential HRT-3 disc assessment did as well or better than 'experts' judging monoscopic photos [97]. Simultaneous stereoscopic disc photography has been considered a gold-standard but is rarely available. Worsening of disc damage will be defined as a rate of neuro-retinal rim loss exceeding 1% of baseline rim area/year on a minimum of 5 repeat HRT images. This slope value is selected as approximately double that of age-related rim area loss [98] and gives a similar specificity to VF trend analyses. Images will be independently assessed by masked observers at the Trial Management Centre using automated algorithms.

Progression of Glaucoma is defined as: '*Strong evidence'*: GPA 'Likely progression' and/or HRT rim area >1% per year (p <0.001); '*Less strong evidence'* = GPA 'Possible progression' and/or HRT rim area >1% per year (p <0.01).

7.11 Proposed frequency and duration of follow-up

7.11.1 Follow up procedure

Patients experience a greater requirement for hospital visits with drop treatment (due to changes in therapy and adverse reactions). These may influence HRQL and form a significant proportion of treatment pathway costs so will help determine important secondary outcomes. Follow-up intervals will lie within the ranges specified by NICE Guidance [1] and will be independently determined on the basis of IOP control or adverse reactions, to minimise bias.

The main driver for follow-up frequency is Treatment in Pursuit of Control (TPC). We will consider disease stability with all available data, but testing for progression does not independently determine follow-up. Anticipated frequency of follow-up is set at entry to the study, based on disease severity and lifetime risk of loss of binocular vision. Subjects who require medication changes or additional laser, suffer adverse events or show progression of glaucoma will be seen sooner and revert to schedule when stable.

See Appendix 4 (section 29) for detailed schedule of assessment intervals for different disease severities.

The worst or more unstable of the patient's two eyes will determine follow-up interval while clearly treatment will be individualised to the needs of each eye.

7.11.1.1 Initial patient contact: assessment of eligibility; written trial information given.

7.11.1.2 Baseline investigations: Consent & Randomisation visit with first treatment.

Informed consent, baseline investigations and initial treatment will take place at a separate visit to the assessment of eligibility & invitation to participate (to minimise regression to the mean); at least 24 hours after initial contact (to allow time for reflection) but within 4 weeks of identification (to minimise delay in treatment). Other than a single extra assessment visit this study mirrors two alternative current medical care pathways and does not include additional tests or visits.

7.11.1.3 Follow-up schedule after changes to treatment:

After any laser the Laser-1st group will be reviewed at 2 weeks and 8 weeks post-laser. Thereafter, and for all treatment changes in the Medicine-1st group, the subjects will be reviewed at 2 months *except* for Severe OAG who will be reviewed at 4 weeks: followed by *either* treatment change (with consequent early assessment of response to 2nd Treatment) *or* entry into disease severity-tailored routine follow-up schedule.

7.11.1.4 Follow-up schedule with suspected progression:

If an eye shows *possible* progression then the follow-up will be intensified to every 3-4 months, until progression is confirmed or ruled out with additional VF / HRT.

7.11.1.5 Additional eye clinic visits:

All contacts with medical professionals and optometrists will be captured for cost data. Contact with healthcare providers will be collected via a Client Service Receipt Inventory [99;100] (CSRI) – a validated method of collecting healthcare cost data (see Appendix 11, section 36 for specific questions asked). A judgement will be made as to the ophthalmic relevance. "Related visits" will be recorded with details while others will be logged in summary form.

7.11.1.6 Timing of Follow-up Investigations

See (section 7.14 below) for greater detail & table of investigations.

After full baseline assessment all patients will undergo VF and HRT to assess progression at each follow-up visit except those immediately following a treatment change. The time intervals are determined by The Pursuit of Control methodology as described above.

Additional visits for IOP check alone after treatment changes are not associated with additional tests. EQ-5D will be assessed at baseline and 6 monthly thereafter, with additional questionnaires as outlined in Appendix 11 (section 36) and the schedule of investigations below (section 7.14).

7.12 Subsequent assessments

All study procedures and assessments are part of routine care.

The figures for the timing of follow-up visits are all provisional durations. They depend on disease severity (more severe more often, following defined criteria) and assume that the patient is at Target IOP without additional visits for IOP-checks or possible progression etc. Additional visits for additional VF, HRT or IOP assessments may be necessary in addition to those listed – as per tightly defined criteria.

E.g. OHT at target may not require an 18 month visit while a patient with unstable severe OAG may be seen 3 or 4 monthly for 3 years.

7.13 Notes on Clinical Assessments

All clinical assessments (blood pressure, slit-lamp based tests eg IOP, HRT, Visual Field etc) will be conducted according to strict trial SOPs.

Gonioscopy will be done with a high magnification lens, eg Magnaview, in a darkened room. An ‘open angle’ for the purposes of this study will be defined as no irido-trabecular contact (ITC) in primary position without indentation. (This is more stringent than the widely accepted definition of angle closure, i.e. 6 clock hours of ITC, in order to further minimise the risk of mis-diagnosis).

Due to the wide range of inter-observer variability and test/retest variation in gonioscopy, patients with borderline narrow angles (*any* irido-trabecular contact or any uncertainty on the part of the recruiting clinician) will be reassessed by the principle investigator for that site.

If “Habitual VA” is < 6/12 (worse than 0.30 on logMAR) on baseline or study exit, the subject will have an auto-refraction and repeated VA assessment with the suggested refraction.

Blood pressure will be assessed at baseline and exit.

IOP will form an important trigger for treatment escalation. It is therefore vital to prevent any risk of observer bias, particularly as this is an unmasked study. Therefore an observer masked to the treatment allocation will make all IOP measurements after randomisation, for example the technician or optometrist performing HRT and VF measurements. Reliability of measurements will assured against the PI gold standard by a validation sample of 40 non-trial IOP measurements over a range of IOP values assessed on a Bland-Altman plot.

7.14 Table of study assessments

Investigation	Time of Follow Up*							
	Baseline	1st Post-Rx Check	3rd Visit (6 months)	1st year	18 ** months	2nd year...	... Patient Specific ...	... 3rd year
Clinical Exam (incl. disc & IOP)	Yes	Yes	Yes	Yes	Yes	Yes	Yes	Yes
Dilated Fundus Examination	Yes	-	Yes	Yes	Yes	-	Yes -	Yes -
Gonioscopy	Yes	-	-	Yes	-	Yes	Yes	Yes
Visual Field Test (HVF)	Yes	-	Yes	Yes	Yes	Yes	Yes	Yes
Optic Nerve Imaging (HRT)	Yes	-	Yes	Yes	Yes	Yes	Yes	Yes
EQ-5D questionnaire	Yes	-	-	Yes	-	Yes	Yes	Yes
GUI questionnaire	Yes	-	-	Yes	-	Yes	Yes	Yes
GSS [80] questionnaire	Yes	-	-	Yes	-	Yes	Yes	Yes
CSRI [81;82] questionnaire	Yes	-	-	Yes	-	Yes	Yes	Yes

7.15 Methods

7.15.1 Training of researchers and collection of data

All Trial staff will be carefully trained using trial SOPs developed specifically for this purpose with inter-observer comparisons of all observer-dependent measurements (e.g. Goldmann IOP). All *treating* ophthalmologists will be senior, fellowship-trained glaucoma specialists experienced in SLT with standardised training and direct observation of SLT procedures by the CI.

7.15.2 Laboratory procedures

Not applicable.

7.15.3 Radiology or other procedures

Not applicable.

7.16 Gantt Chart

See Appendix 12 (section 37) for Gantt Chart showing details of major project deadlines.

7.17 Definition of end of trial

The end of the trial will be when the last follow-up visit and self-completion questionnaire has been completed by the last participant after three years of follow-up.

7.18 Discontinuation/withdrawal of participants and 'stopping rules'

We have not defined stopping / discontinuation rules for early termination of the trial because the two treatment pathways are designed to generate equivalent attainment of treatment targets, with differences in treatment-related HRQL and cost and *not* vision. It is likely that the full effects of the different pathways on HRQL and cost will not become apparent until the full three years of follow-up and an early termination based on HRQL or cost might significantly under- or over- estimate the effect of the interventions. No difference in safety outcomes is expected, but of course will be reported as outlined below (section 9) and should the data monitoring committee request interim analyses these will be supplied at least two weeks prior to the meeting of the DMC and TSC.

Subjects would be withdrawn from the trial if they become pregnant, at the time when there is medical indication for an intervention not permitted by the trial algorithms (e.g. SLT for a patient in the Medicine-1st group) and thus withdrawal from the trial is within the best interests of the participant or child. This is a very unlikely event, since the majority of female participants will not be of child-bearing age. Follow-up data will continue to be

collected and analysed on an intention to treat basis. Withdrawal would be immediately notified to the trial sponsor and TSC. There is no plan to replace withdrawn subjects.

8 Name and description of all drugs used in the trial

This trial includes no investigational medicinal products. All medicines used within this study are registered products that form part of the standard medical care of glaucoma. This is a pragmatic study in which broad treatment guidelines define classes of drug that may be used but the specific preparation is chosen by the local PI.

All currently available medical treatments for open angle glaucoma from the following classes are permissible: prostaglandin analogues; beta-blockers; alpha-agonists; topical carbonic anhydrase inhibitors.

Preservative free and combination preparations are permissible.

Generic alternatives are permissible.

Systemic acetazolamide may only be used as a temporising measure before surgery. Pilocarpine (other than as pre-treatment prior to SLT if preferred), and epinephrine do not form part of best-practice treatment of OAG or OHT and are not permitted.

Anti-inflammatory drugs after SLT are restricted to Acular tds if required.

Post-surgical drug regimens are not constrained – any topical steroid / antibiotic drop combinations are permissible.

8.1 'Name and description of each IMP'

Not Applicable (not a CTIMP study).

8.2 'Source of IMPs including placebo'

Not Applicable (not a CTIMP study).

8.3 'Accountability procedures for the IMP, including placebo/comparator'

Not Applicable (not a CTIMP study).

8.4 'Route of administration, dosage, dosage regimen, and treatment period of the IMP'

Not Applicable (not a CTIMP study).

8.5 Dose modifications

This is a pragmatic trial mirroring normal best clinical practice as closely as possible> As such, treatment follows normal clinical practice as outlined in national and international treatment guidelines (NICE [1] and European Glaucoma Society (EGS) guidance [73]). Dose

modifications, for example in the case of certain of adverse events or inadequate IOP control, follow standard switch or add protocols derived from NICE [1] and EGS guidance [73] and are specified in detail in the appendices (see sections 30 and 31).

The absence of stopping rules is justified in section 7.18.

8.6 Assessment of compliance

This pragmatic trial aims to mirror best clinical practice. Participants will be trained in self-administration of eye-drops and asked about compliance following standardised protocols. There will be no additional monitoring of compliance.

8.7 Post-trial IMP arrangements

Not applicable as all medications used are available in normal clinical UK practice.

8.8 Name and description of each NIMP

All medications used within this study are NIMPs. The full range of registered available topical medications for OAG may be used (with the exception of pilocarpine and epinephrine) within the restrictions of the treatment escalation protocol (see appendix 5b, section 30.2). The non-investigational medicinal products (NIMPs) which may be used by the subjects are listed here. In addition topical non-steroidal drugs after SLT, systemic acetazolamide (Diamox) as a temporising measure pending surgery and immediately pre- and post-operative medications (steroids, antibiotics and non-steroidal anti-inflammatory drugs (NSAIDs)) may be used as required.

Any new medications for the treatment of OAG that become available during the course of the trial will be permitted after discussion at, and with the approval of, the TSC.

NIMP suspected Adverse Drug Reactions (ADR) or side effects will be reported through the yellow card system, as is normal practice.

Pilocarpine and depot steroids do not form part of the current standard treatment of OAG and will not be used in the course of this trial without additional indications.

First-Line Drugs: Prostaglandin analogues (PGA)

- Latanoprost
- Bimatoprost
- Travoprost
- Tafluprost

Second-Line Drugs: Beta blocker (once in the morning or in a PGA combination)

- Timolol (0.1%, 0.25%, 0.5%)
- Betaxolol

- Carteolol

Third and Fourth Line Drugs: Topical carbonic anhydrase inhibitor (CAI) and alpha-agonists

- Dorzolamide
- Brinzolamide
- Iopidine
- Brimonidine

Combination Preparations

- Cosopt
- Azarga
- Combigan
- Ganfort
- Duotrav
- Xalacom

Anti-inflammatories (steroids)

- Dexamethasone
- Prednisolone (topical and oral)
- Loteprednol
- Fluorometholone

Anti-inflammatories (NSAIDs)

- Ketorolac (Acular)
- Nepafenac (Nevanac)
- Bromfenac
- Diclofenac
- Flurbiprofen

Antibiotics

- Chloramphenicol
- Maxitrol
- Tobramycin

Acetazolamide and DIAMOX SR (oral)

Any preservative-free topical lubricants (‘artificial tears’).

9 Recording and reporting of adverse events and reactions

9.1 Definitions

Adverse event means any untoward medical occurrence in a subject to whom a medicinal product has been administered, including occurrences which are not necessarily caused by or related to that product;

Adverse reaction means any untoward and unintended response in a subject to an investigational medicinal product which is related to any dose administered to that subject;

Serious adverse event, ***serious adverse reaction*** or ***unexpected serious adverse reaction*** means any adverse event, adverse reaction or unexpected adverse reaction, respectively, that:

- results in death,
- is life-threatening,
- requires hospitalisation or prolongation of existing hospitalisation,
- results in persistent or significant disability or incapacity, or
- consists of a congenital anomaly or birth defect;

Important medical events that may not be immediately life-threatening or result in death or hospitalisation but may jeopardise the subject or may require intervention to prevent one of the outcomes listed in the definition of serious will also be considered serious.

Unexpected adverse reaction means an adverse reaction the nature and severity of which is not consistent with the information about the medicinal product in question set out in the summary of product characteristics (SmPc) for that product. The most up-to-date version of the SmPc will be used during the trial.

Suspected unexpected serious adverse reaction is also known as a **SUSAR**.

9.2 'Expected Adverse Events'

A number of different drugs may be used in this study (as listed in section 8.8 under NIMPS) and all are known to have potential adverse reactions. These are listed fully in the appropriate package inserts but the more common or more severe are listed here, by drug class.

SLT is also associated with some adverse reactions and these too are listed here.

9.2.1 Prostaglandin analogues (PGA):

Excessive lash growth, conjunctival injection, ocular irritation, allergy, peri-ocular pigmentation, discomfort, itching, rash, stinging on instillation, change in iris colour, macular oedema, reactivation of herpes simplex keratitis, worsening of uveitis.

9.2.2 Beta blocker (once in the morning or in a PGA combination):

Asthma, shortness of breath, heart block, cardiac arrhythmia, reduced exercise tolerance, allergy, dry eye, stinging on instillation, impotence, depression.

9.2.3 Topical carbonic anhydrase inhibitor (CAI):

Allergy, discomfort, itching, rash, stinging on instillation, taste disturbance

9.2.4 Alpha-agonist:

Allergy, discomfort, itching, rash, somnolence, worsening of angina, stinging on instillation, depression.

9.2.5 Anti-inflammatories- steroids:

Stinging on instillation, allergy, discomfort, itching, rash, raised intra-ocular pressure.

9.2.6 Anti-inflammatories- NSAIDs:

Stinging on instillation, allergy, discomfort, itching, rash.

9.2.7 Antibiotics:

Stinging on instillation, allergy, discomfort, itching, rash.

9.2.8 Acetazolamide:

Rash, diarrhoea, renal stones, hypokalaemia, Stevens-Johnsons syndrome, taste disturbance, parasthesiae, blood dyscrasias.

9.2.9 Any preservative-free topical lubricants ('artificial tears'):

Temporary blurred vision

9.2.10 SLT: intra-ocular pressure spike:

Inflammation; discomfort; blurred vision; endothelialitis (very rare), change in refraction (very rare), intra-ocular haemorrhage (very rare).

9.3 Recording adverse events

All adverse events will be recorded in the hospital notes in addition to the CRF.

A record will also be kept in the CRF of ALL adverse events, whether believed to be related or unrelated to the treatment.

The record of adverse events will include the following.

- Clinical symptoms: a simple, brief description.
- Severity. The following categories will be used:

  Mild: the adverse event does not interfere with the volunteer's daily routine, and does not require intervention; it causes slight discomfort.

  Moderate: the adverse event interferes with some aspects of the volunteer's routine, or requires intervention, but is not damaging to health; it causes moderate discomfort.

  Severe: the adverse event results in alteration, discomfort or disability which is clearly damaging to health.
- Relationship to treatment: The assessment of relationship of adverse events to the treatment received is a clinical decision based on all available information at the time of the completion of the case report form. The following categories will be used:

  Definitely: There is clear evidence to suggest a causal relationship, and other possible contributing factors can be ruled out.

  Probably: There is evidence to suggest a causal relationship, and the influence of other factors is unlikely.

  Possibly: There is some evidence to suggest a causal relationship (e.g. the event occurred within a reasonable time after administration of the laser or medication). However, the influence of other factors may have contributed to the event (e.g. the patient's clinical condition, other concomitant events).

  Unlikely: There is little evidence to suggest there is a causal relationship (e.g. the event did not occur within a reasonable time after administration of the trial medication). There is another reasonable explanation for the event (e.g. the patient's clinical condition, other concomitant treatments).

  Not related: There is no evidence of any causal relationship.

  Not Assessable
- Expectedness: The following categories will be used:

  Expected: An adverse event that is classed as serious *and* which is consistent with the information in the SmPC about the licensed drugs used in the treatment pathway, or clearly defined in this protocol.

  Unexpected: An adverse event that is classed as serious and which is not consistent with the information about in the SmPC for the licensed drugs used in the treatment pathway,

Seriousness as defined for an SAE in section 9.1.

Collection, recording and reporting of adverse events (including serious and non-serious events and reactions) to the sponsor will be done according to the sponsor's SOP.

9.4 Procedures for recording and reporting Serious Adverse Events

All the medicinal products and devices used in this trial are licensed in the UK (CE marked) and used within their marketing authorization,

All serious adverse events will be recorded in the hospital notes and the CRF, and the sponsor's SAE log. The SAE log must be reported to the sponsor at least once or twice per year.

The Chief or Principal Investigator will complete the sponsor's serious adverse event form and the form will be sent to the sponsor within one working day of his / her becoming aware of the event. The Chief or Principal Investigator will respond to any SAE queries raised by the sponsor as soon as possible.

All serious events occurring at collaborating sites will be reported to the CI in addition to the sponsor. Any safety information arising from these reports will be disseminated to collaborating PIs by email by the CI within a week of the decision to pass on the information.

All SUSARs will be notified to the sponsor immediately (or at least within one working day) according to the sponsor's written SOP.

Reporting to the sponsor will be done as per the sponsor's SOP.

9.4.1 Notification of deaths

No deaths are expected to be related to an ophthalmic treatment for glaucoma, however all deaths, including deaths deemed unrelated to the trial treatments, will be reported to the sponsor within one week of the PI being notified.

9.4.2 Reporting SUSARs

The sponsor will notify the main REC and MHRA of all SUSARs. SUSARs that are fatal or life-threatening must be notified to the MHRA and REC within 7 days after the sponsor has learned of them. Other SUSARs must be reported to the REC and MHRA within 15 days after the sponsor has learned of them.

9.4.3 Annual safety reports

The sponsor will provide the main REC and the MHRA with an annual safety report (ASR). The ASR will be prepared, using the sponsor's ASR form, by the Chief investigator or a delegated PI, reviewed by the sponsor and when necessary be referred to an independent

committee (independent to the trial) such as the safety committee. This will be done in accordance with the sponsor's SOP.

9.4.4 Annual progress reports

An annual progress report (APR) will be submitted to the REC within 30 days of the anniversary date on which the favourable opinion was given, and annually until the trial is declared ended.

The chief investigator will prepare the APR.

9.4.5 Pregnancy

Any participant falling pregnant during the study will exit the trial to allow permit a full choice of appropriate treatment options by the treating clinician (e.g. allowing SLT for a pregnant patient who is in the Medicine-1st arm). All such pregnancies will therefore be recorded and notified to the sponsor.

As no investigational medicinal product or intervention is used in this trial there will be no additional follow-up of pregnant subjects beyond normal clinical care, nor of children born to pregnant trial subjects. The subject will remain under close clinical supervision and, for purposes of safety reporting, continue to have outcome data recorded by the trial clinicians.

9.4.6 Reporting Urgent Safety Measures

Regulation 30 of the Medicines for Human Use (Clinical Trials) Regulations 2004 [Statutory Instrument 2004/1031], as amended by Statutory Instrument 2006/1928 states "the Sponsor and the Investigator may take appropriate urgent safety measures in order to protect the subjects of a clinical trial against any immediate hazard to their health or safety. If measures are taken, the Sponsor shall immediately and in any event no later than 3 days from the date the measures are taken, give written notice to the MHRA and the relevant REC of the measures taken and the circumstances giving rise to those measures."

In order to prevent any delays in the reporting timelines the sponsor has delegated this responsibility to each PI site. Therefore the PI must report any urgent safety measures to the MHRA directly, and in parallel to the sponsor.

9.4.7 Notification of Serious Breaches to GCP and/or the protocol

Any deviations, violations, potential serious breaches and urgent safety measures will be recorded in the trial log and reported to the sponsor immediately.

Regulation 29A of the Medicines for Human Use (Clinical Trials) Regulations2004 [Statutory Instrument 2004/1031], as amended by Statutory Instrument2006/1928,

contains a requirement for the notification of “serious breaches” of GCP or the trial protocol.

Thus:

(1) The sponsor of a clinical trial shall notify the licensing authority in writing of any serious breach of -(a) the conditions and principles of GCP in connection with that trial; or (b) the protocol relating to that trial, as amended from time to time in accordance with regulations 22 to 25, within 7 days of becoming aware of that breach.

(2) For the purposes of this regulation, a “serious breach” is a breach which is likely to effect to a significant degree –

(a) the safety or physical or mental integrity of the subjects of the trial; or

(b) the scientific value of the trial.

The sponsor will be notified immediately of any case where the above definition applies during the trial conduct phase. The sponsor’s SOP on the ‘Notification of violations, urgent safety measures and serious breaches’ will be followed.

9.5 The type and duration of the follow-up of subjects after adverse events.

Following an adverse drug reaction or complication of laser treatment within the trial subjects will be monitored for the remaining duration of their trial follow-up and thereafter continue under the care of the treating physician under normal NHS care. The CI will remain a point-of-contact for the reporting of any suspected late-onset complications for at least 5 years after trial completion.

Any SUSAR related to the IMP will need to be reported to the Sponsor irrespective of how long after treatment the reaction has occurred.

10 Data management and quality assurance

10.1 Confidentiality

No patient identifiable information (name, date of birth, address) will be retained within the trial records. Any personal data (such as medical history) will be identifiable only by a unique, private trial identification code. This code will be used for all trial investigations such as fields, imaging and trial records (CRFs). We shall comply with all GCP stipulations on confidentiality, the Standard Operating Protocols of our collaborating MHRA registered Clinical Trials Unit, PRIMENT, the Data Protection Act 1998 and the Trust Information Governance Policy of each collaborating site.

10.2 Data collection tools and data handling

Identical electronic and hard copy case report forms (CRF) will be designed and produced by the investigator, according to a standard CRF template and the final version will be approved by the sponsor.

A web-based database managed by 'SealedEnvelope' for PRIMENT CTU will be used for database entry with direct data entry at the time of patient visit. This will include extensive internal consistency and range checking, with hard copy backup CRF in case of IT failure. Records will be identifiable only by unique, confidential trial identification number without patient-identifiable information included. All data will be contemporaneously entered *either* directly into the web-based database CRF *or,* in the event of IT failure, onto hard copy CRF of identical lay-out with later entry onto the database (within 3 days of collection).

All electronic data-entries will be subject to an audit trail to record alterations (where permitted). Hard-copy entries will be made legibly in black ink with a ball-point pen. If an error is made, the error will be crossed through with a single line in such a way that the original entry can still be read. The correct entry will then be clearly inserted, and the alterations will be initialled and dated by the person making the alteration. Overwriting or use of correction fluid will not be permitted.

Data from patient completed questionnaires will be scanned upon receipt for e-copy back-up and entered onto the database within one week of receipt by the trial data-management officer. Questionnaire data are from validated, standardised tools (EQ-5D, GUI, GQL-15, GSS, CSRI). The questions to be asked are included as in Appendix 12 (below). It will be the responsibility of the investigator to ensure the accuracy of data entered in the CRFs. The delegation log will identify all those personnel with responsibilities for data collection and handling, including those who have access to the trial database.

Data for the primary outcome measure (EQ5D) will be double-entered and any discrepancy cross-checked against the original. A sample of 5% of all data will be error-checked.

11 Record keeping and archiving

No patient identifiable information (name, date of birth, address) will be retained within the trial records. Any personal data (such as medical history) will be identifiable only by a unique, private trial identification code. This code will be used for all trial investigations such as fields, imaging and trial records (CRFs). We shall comply with all GCP stipulations on confidentiality, the Standard Operating Protocols of our collaborating MHRA registered Clinical Trials Unit, PRIMENT, the Data Protection Act 1998 and the Trust Information Governance Policy of each collaborating site.

All trial records (master file, site files, CRFs and consent forms), physical and electronic, will be kept in locked premises at all times. Each site will require a secure, locked store for physical records that might potentially be able to be linked back to the patient (via the

unique trial identification code). All electronic records (via web-based entry to off-site data storage) will involve secure electronic encryption of off-site back-ups and databases via a third party research trials database management company ("SealedEnvelope"). The third party research trials database management company use secure fully-accredited servers certified to industry standards for security and safety (ISO 27001; ISAE 3402 Type II; PCI Data Security Standard (PCI DSS)).

The trial disaster recovery plan stipulates offsite storage of the main trial database (contemporaneous data collection will minimise risk of data loss) using secure encrypted mirrored data storage servers at a minimum of two separate geographical locations. The Chief Investigator is responsible for the secure archiving of the trial database and trial documents at each site.

12 Statistical Considerations

The Lead Trial Statistician is Dr Gareth Ambler, Lecturer in Medical Statistics at UCL & Joint UCL/UCLH Biomedical Research Unit, who has been involved in the design of the trial and will lead the analysis.

12.1 Outcome Measures

12.1.1 Primary Outcome Measure: Health-related Quality of Life

The primary outcome measure is Health Related Quality of Life (HRQL). We shall measure interview administered EQ-5D using the 5*3 descriptive system and convert the resulting health states into zero-one single summary indices using the appropriate UK-specific algorithm (Time Trade-Off valuation) [101]. We have specified a 3-year duration for the trial as this reflects the point at which we anticipate that 50% of laser-treated patients will have received additional therapy [64]. We have powered the study to look for superiority of EQ-5D at three years.

QALYs will be calculated for the total 36 month period using baseline and 6 month follow up EQ-5D [74] health states used to calculate utility scores as recommended by NICE [102]. We will collect EQ-5D data 6 monthly for each patient. Patient-specific utility profiles will be constructed assuming a straight line relation between each of the patient EQ-5D scores at each follow-up point. The QALYs experienced by each patient from baseline to three years will be calculated as the area underneath this profile. Multiple imputation by chained equations will be used to deal with missing EQ-5D and resource use values. Subsequent analyses of imputed data will include variance correction factors to account for additional variability introduced into parameter values as a result of the imputation process.

12.1.2 Secondary Outcome Measures

12.1.2.1 Treatment Pathway Cost and Cost-Effectiveness

We will undertake a detailed analysis of the cost and cost-effectiveness of Laser-1st compared to Medicine-1st.

Cost data will be ascertained directly from the record of trial-related treatment episodes and about additional contacts with healthcare providers via a modified 'Client Service Receipt Inventory' (CSRI). This is a validated method for designed to capture data on other healthcare contacts [99;100] which has been used successfully in a self-completion questionnaires format [99;100]. The CSRI questionnaire will be sent out at 6 monthly intervals at the time of the EQ5D collection (see Appendix 11, section 36 for details).

We will estimate cost and cost-effectiveness for the within-trial period (3 year/short-run model) and also over the expected lifetime of the patient (lifetime/long-run model). The analyses will be conducted from a health services perspective. The cost-effectiveness measure in the 3 year/short-run model will be the incremental cost per QALY gained of Laser 1st versus Medicine-1st. This will be calculated as the mean cost difference between Laser-1st and Medicine-1st divided by the mean QALY difference to give the incremental cost-effectiveness ratio (ICER). The cost components included in the analysis will consist of the cost of SLT (including annuitised capital costs), number of ophthalmologist visits, number and type of glaucoma medications, number and type of glaucoma surgeries, and all clinical tests, including IOP assessments, optic nerve assessments, retinal or macular examinations, slit lamp examinations and gonioscopy. Unit costs will be taken from standard published sources. The volume of resource use for each cost component will be measured directly in the trial from both patient records and patient diaries. QALYs for the full 3 year follow-up period will be calculated from EQ-5D data based on values from Dolan [101] and calculating the area under the curve, using the methodology stated above. As baseline utility scores are not controlled for prior to randomisation, utility scores may artificially differ between trial arms at baseline. Regression analysis will be used to control for differences in baseline utility scores.

We will use non-parametric methods for calculating confidence intervals around the ICER based on bootstrapped estimates of the mean cost and QALY differences [103]. The bootstrap replications will also be used to construct a cost-effectiveness acceptability curve, which will show the probability that Laser 1st is cost-effective compared to Medicine-1st at 3 years for different values of the NHS' willingness to pay for an additional QALY. We will also subject the results to extensive deterministic (one-, two- and multi-way) sensitivity analysis.

In the lifetime model cost-effectiveness will also be calculated in terms of the incremental cost per QALY gained of Laser-1st versus Medicine-1st. The model will be developed and populated based on available evidence, including the data collected during the trial. Based on previously identified models [22], the proposed design is a Markov state-transition model that allows movement between glaucoma states. Data from results of the LiGHT trial will be used to estimate values for the first 3 years of the model. Values in the model for 4

years until death will be estimated based on assumptions from the LiGHT findings, systematic searches of the literature to identify existing models, the results of comparable trials in other countries and national databases to estimate mortality and morbidity. The model will have cycles of one year duration and calculate expected costs and outcomes for a synthetic cohort of patients aged 40 years and older until all patients have died. Costs and QALYs will be discounted at 3.5% per year, in line with NICE guidelines [102]. Model states will include ‘mild glaucoma’, ‘moderate glaucoma’, ‘severe glaucoma’ and ‘visual impairment’, which will be clearly defined with associated costs and utility values [104;105]. Transition probabilities will be obtained from the LiGHT findings for the first 3 years of the model and a combination of published studies and LiGHT findings for the remaining years of the analysis. A review of a previously identified model [22] suggests that there are sufficient data for this to be feasible. Given the duration of follow-up in the trial, health status utility and annual costs associated with each Markov state will be based on within-trial data; mean utilities and costs for each state will be calculated based on the patient-level data in the three year follow-up period in the study. These values will then be utilized in the long-run model. The within-trial values will also be compared and supplemented with where appropriate data from published studies (see e.g., Traverso et al [106]). We will undertake deterministic (one-, two- and multi-way) and probabilistic sensitivity analysis, the latter assuming appropriate distributions and parameter values [107]. The values from the probabilistic sensitivity analysis will be used to construct a cost-effectiveness acceptability curve, which will show the probability that Laser 1st is cost-effective compared to Medicine-1st for the full life time of patients for a range of values of the NHS’ willingness to pay for an additional QALY.

12.1.2.2 Glaucoma-specific treatment-related quality of life: Glaucoma Utility Index [83] (GUI)

The Glaucoma Utility Index [83] (GUI) is utility-based glaucoma health outcome measure. specifically designed to capture the impact of glaucoma treatment and disease severity on HRQL. Five questions (each testing different ‘domains’) ask about difficulties with certain activities and are scored by the subject as *None*; *Some*; *Quite a lot* or *Severe.*

Utility estimates derived from the GUI decrease as expected with increasing severity, defined both subjectively (self reported) and objectively (classified by increasing visual field loss) [83].

12.1.2.3 Patient Reported Disease and Treatment Related Symptoms: Glaucoma Symptom Scale [84] (GSS)

The Glaucoma Symptom Scale [84] (GSS) consists of 2 subscales: 6 items that identify non-visual ocular symptoms (such as dryness or itching) and 4 items that identify visual ocular symptoms (such as difficulty seeing in dark places or halos around lights). The former subscale measures symptoms in and around the eye, unrelated to the visual function of the eye. The latter measures symptoms of visual disturbance, unrelated to the non-visual sensations experienced by the eye.

The GSS is generated for each eye: a 5-level score is generated, ranging from 0 (complaint present and very bothersome) to 4 (complaint absent). This score is then

transformed to a 0 to 100 scale, with 0 representing presence of a very bothersome problem and 100 representing absence of a problem. The final GSS score is an un-weighted average of the responses to all 10 items, averaged between the 2 eyes. Scores can be generated for each eye individually also. Final GSS subscale scores are an un-weighted average of all items that comprise the particular subscale, averaged between the 2 eyes.

GSS appears to be a valid and reliable measure across a broad range of treatment groups and disease severities.

12.1.2.4 Patient Reported Visual Function: Glaucoma Quality of Life - 15 [8] (GQL-15)

The Glaucoma Quality of Life-15 [8] (GQL-15) is a concise, easy to administer 15 item questionnaire [8;10] that independent reviews have described as one of the better glaucoma-specific instruments, with good patient acceptability [108;109]. Derived from a 62-item pilot instrument, the 15 items were chosen for their strong relationship with visual field loss in glaucoma patients. Several studies have used the GQL-15 [110-113] and found that it correlated well with objective measures of visual function and more severe glaucoma and discriminated between quality of life in patients with and without glaucoma. In patients with glaucoma or ocular hypertension the GQL-15 summary score was an independent risk factor for depression [110]. While the name of the instrument suggests that it measures vision-related quality of life, all the items actually refer to activity limitation (near vision, peripheral vision, mobility, and dark adaptation).

Its use as a secondary outcome measure in a planned SLT RCT in Australia will facilitate comparison of visual function outcomes (collaboration agreed, Prof Crowston, Melbourne).

12.1.2.5 Objective measures of pathway effectiveness and visual function

The Treat in Pursuit of Control design is expected to lead to a different intensity of intervention in each pathway. Objective measures will record the effectiveness of each arm in achieving the therapeutic aim of lowering IOP. Medical treatment is a risk factor for earlier cataract development, so we will also monitor cataract extractions.

Efficacy and intensity of the treatment pathways will be assessed at 3 years. We will measure following:

1. The number of clinic visits and medical contacts over 3 years.
2. The intensity of treatment used to achieve Target (number of patients with: multiple SLT treatments; multiple medications; number of patients receiving glaucoma surgery).
3. The *time* taken to reach Target and the *number of revisions* of the Target (if initial Target cannot be met without surgery) will measure the ability to achieve the Target for each group.
4. The *proportion* of patient achieving Target after each year of treatment.
5. The number of patients with confirmed deterioration of visual field or optic disc appearance in each group.

6. Rates of cataract and trabeculectomy surgery monitored by event reporting during the trial.

Objective measures of visual function (visual acuity, HVF) will also be assessed as part of standard clinical monitoring and as a safety measure in addition to patient reported measures (GQL-15, above). With the Treat in Pursuit of Control design we do not expect significant differences in the IOP achieved, only intensity of treatment. Visual outcomes are not expected to differ but will be measured.

12.1.2.6 Objective measures of the safety profiles of each pathway

Adverse events possibly associated with treatment will be recorded. These include (but are not restricted to): post-laser IOP spikes >30mmHg or >30% increase within the first 4 weeks; anterior uveitis requiring a change in treatment; treatment changes due to adverse drug reactions; any sight threatening adverse events; ocular surface diseases; drop intolerances causing treatment changes; subjective local side effects noted by patient but tolerated (stinging, hyper-pigmentation; hyperaemia etc); new diagnoses of asthma, COPD, heart block; cataract surgery. In addition we shall compare transient ocular discomfort (using a visual analogue scale ranging from 0-10); and IOP fluctuation (standard deviation of IOP at visits after the initial treatment will be used as a surrogate for IOP fluctuation).

Participants will be asked about possible treatment related side effects using a simple standardised series of closed and open questions at each visit (see Appendix 13, section 38).

12.1.2.7 Concordance / Compliance

A pair of questions will be asked about drop usage and compliance that have been validated in a large study of compliance with drop therapy in glaucoma (personal communication, Prof David Friedman, Johns Hopkins) and shown to be predictive of non-compliance.

12.2 Sample size and recruitment

12.2.1 Sample size calculation

The primary outcome measure is health-related quality of life (HRQL) powered to detect superiority of a treatment pathway at 36 months. As recommended by NICE methodological guidelines [102] when conducting economic evaluations in adults the HRQL, will be determined using EQ-5D profiles [74;101]. Since we expect there to be no survival difference between groups extrapolation of life expectancies will be applied to the EQ-5D-profile derived utilities to determine QALYs.

A clinically meaningful difference in EQ-5D utility was considered to be 0.05 in an ophthalmology study of glaucoma surgery [114] and 0.074 in a more general setting [115]. These are less than the difference in EQ-5D reported between mild (0.84+/- 0.17) and moderate (0.68 +/-0.26) glaucomatous visual field loss in UK patients [116]. We have

selected the 0.05 effect size used in the EAGLE study [114], an MRC funded trial of 400 patients looking at treatments for Angle Closure Glaucoma, as the more stringent margin.

The UK Glaucoma Treatment Study (UKGTS) recently recruited over 500 treatment-naive patients with mild glaucoma, similar to our patient group and from several of the centres participating in this study. UKGTS found mean EQ-5D utility at enrollment to be 0.858 (SD 0.197). This is close to values in the literature (0.76 ± 0.19 (SD)[116] and that used by EAGLE [114] (SD 0.14). Four of our five centres recruited for UKGTS with proven track records. A study with 311 participants in each group would have 90% power to detect at 5% significance level a difference in means of 0.05, assuming that the common standard deviation is 0.19 and using a two-sample t-test (we may gain precision using ANCOVA). Allowing for 15% loss to follow-up at 36 months, the total number required for the study is **718** (359 in each group). The sample size was calculated, for a t-test, using Stata 12.

The Treat in Pursuit of Target design is expected to produce comparable outcomes in IOP and visual function.

12.2.2 Planned recruitment rate

Details of recruitment rate assumptions and calculations are given in section 6 of this protocol and shown graphically in Appendix 2 (section 27).

To recruit 718 in 2 years we need to recruit an average 30 patients per month, from all 5 sites. This represents 28% of those eligible and 40% of those provisionally willing to participate in research. We assume an attrition rate of 15% at 3 years, based on our UKGTS experience and since this is a pragmatic trial without additional visits or examinations.

12.3 Statistical analysis plan

12.3.1 Summary of patient flow

The details of the number of eligible patients for the trial, number consenting and number randomised are given in section 6 above and shown graphically as a Consort flow-diagram in Appendix 2 (section 27).

12.3.2 Primary endpoint analysis

A single main analysis will be performed at the end of the trial when follow-up is complete. Interim analyses may be conducted for the DMC if requested as per agreed terms of reference but there is no planned interim analysis examining efficacy and hence no adjustment to inflate the sample size. The statistical analysis will be based on all participants as randomised, irrespective of subsequent compliance with allocated treatment (Intention To Treat Analysis). A CONSORT diagram will be used to describe the course of patients through the trial. Baseline characteristics will be summarised by randomised group. Summary measures for the baseline characteristics of each group will

be presented as mean and standard deviation for continuous (approximate) normally distributed variables, medians and inter-quartile ranges for non-normally distributed variables and frequencies and percentages for categorical variables. The primary outcome will be compared between treatment arms using regression methods (analysis of covariance) that adjust for the randomisation factors: severity and centre (as recommended in ICH E9, section 5.7) (and appropriate baseline values of outcome including laterality). Statistical significance will be at 5%.

We intend to use mixed models to investigate how primary and secondary outcomes change over time. Such models allow analysis of repeated outcome measurements data (recorded every 6 months) while taking into account the correlation between measurements from the same patient. By using interaction terms between randomisation group and time, we will to investigate differences between groups over time. Regression splines will be used to explore non-linear trajectories, if such exist. The mixed models will also provide estimates (with confidence intervals) of differences in outcomes at any point over the three years. A sample size calculation based on ANCOVA (as for the main analysis) suggests 91% and 92% power respectively to detect differences in EQ-5D at these time-points, assuming the same effect size proposed for the 3 year analysis and linear attrition. The mixed model should have similar or greater power due to efficient use of repeated measurements data.

Patients will inevitably be lost to follow-up by 36 months. Our sample size assumes 15% of patients would not provide an evaluable 36 month EQ-5D. If this rate occurs many patients will be only partially observed. Reasons for absence may be important and will be investigated using logistic regression of covariates on an indicator of absence. Missing data statistical modelling techniques will be used to make use of outcome assessments prior to 36 months and sensitivity analyses will be conducted to assess the appropriateness of the treatment estimates to these approaches. The unit of analysis for the primary outcome is the patient, with bilateral disease included as a covariate. Presentation of all findings will be in accord with the latest CONSORT statement [117]. Our choice of secondary outcome (GQL-15 questionnaire) will permit meta-analysis (already agreed with Prof Crowston, Melbourne) with a similar Australian laser RCT (although they will not use the real-world 'Treat in Pursuit of Target' strategy that we employ).

12.3.3 Secondary endpoint analysis

The use of hypothesis tests would be inappropriate as the study has not been powered to address these for secondary analyses and so these will be considered as hypothesis generating rather than providing firm conclusions.

12.4 Randomisation

Online randomisation (with blocking with random block sizes) will be used to randomise at the level the patient and be stratified by diagnosis (OHT/OAG) and treatment centre as stratification covariates with equal allocation between treatment arms. The primary

analysis will adjust for the stratification factors used in randomisation. Participants will randomised to one of the two study groups in equal proportion using a web-based randomisation service provided by a specialist company to achieve full allocation concealment (www.sealedenvelope.co.uk), available 24/7. ‘SealedEnvelope’ will also hold the randomisation list. A backup telephone service will be available.

12.5 Interim analysis

No interim analyses are planned in this study. A single main analysis will be performed at the end of the trial when follow-up is complete. Interim analyses may be conducted for the DMC if requested as per agreed terms of reference but there is no planned interim analysis examining efficacy (and hence no adjustment to inflate the sample size).

We have not defined stopping / discontinuation rules for early termination of the trial because the two treatment pathways are designed to generate equivalent attainment of treatment targets, with differences in treatment related HRQL and cost - *not* vision. No difference in safety outcomes is expected, but of course will be reported as outlined above (section 9) and should the data monitoring committee request interim analyses these will be supplied at least two weeks prior to the meeting of the DMC and TSC.

As this study is unblinded there is no arrangement for breaking of the randomisation code.

12.6 Other statistical considerations

All study analyses will be according to the Statistical Analysis Plan (StAP), agreed in advance by the Trial Steering Committee (TSC). In the event of a deviation from the original statistical plan, any deviation will be described and justified in the protocol and/or in the final report.

All statistical and health economic analyses will be made masked to the treatment allocation.

13 Committees involved in LiGHT

The trial will include a Trial Management Group (TMG), Independent Data Monitoring Committee (IDMC) and Trial Steering Committee (TSC) (members to be confirmed). The terms of reference for these committees follow Moorfields and PRIMENT(UCL) SOPs and are available upon request.

13.1 Study Co-Ordination In London (Central Trial Office)

The Trial Management Team will comprise Chief Investigator (CI), Central Trial Manager (CTM), Central Trial Fellow, Central Trial Technician and will meet a minimum of monthly for the duration of the trial, to ensure smooth running and trouble-shooting. The duties of the CTM are to support the organisation of the study (investigator meetings, TSC, and DMC meetings, training etc), have a study management role, including monitoring data

collection according to established milestones, maintaining trial records, coordination between local sites and central CTU data management, facilitation of user involvement in the project through Lay Advisory Group meetings, and working alongside the CTF facilitating the recruitment and follow-up of study participants. The Local Trial fellows will be directly accountable to the Local PIs. They are responsible for recruitment, treatment and follow-up of trial participants. There will be bi-monthly conference-calls to all local TFs and PIs to troubleshoot local issues. The CI will closely supervise the CTM and CTF with weekly meetings for the duration of the trial. The Trial Management Group (CI, CTM and co-investigators) will meet an average of 6 monthly after the first year.

13.2 Local Organisation In Centres

The local lead consultant ophthalmologist, all glaucoma sub-specialists, will be the local prime investigators (PI), coordinate local ethics approval and sit on the TSC. The local study coordinator will administrate the follow-up and recall of patients, liaising with the Central Trial Office. The local Trial Fellow will be an ophthalmologist or optometrist who will recruit, examine, treat (with local PI) and monitor the trial subjects. They will have monthly conference calls with the Central Trial Office Team for the duration of the study.

13.3 Trial Steering Committee (TSC)

The TSC will be comprised in accordance with GCP: we propose the following members: Prof John Sparrow, as Independent TSC Chair; Mr Gus Gazzard, (CI); Dr James Morgan, (An independent clinician with relevant expertise); Maria Hassard, (Sponsor representative); Dr Marta Buszewicz, (CEL CLRN representative); Dr Luke Vale, (Health Economist); Marta Van der Hoek “Garcia-Finana”, (independent Statistician); Susan Newell and T Sheila Page, (patient representatives). The trial manager, (Dr Amanda Davis), CI and trial statistician, (Dr Gareth Ambler) will be invited to report as required. The TSC will meet at least 6 monthly with minutes.

13.4 Data and Safety Monitoring (DMC)

Data and safety monitoring by the UCL PRIMENT CTU will include regular reports from the CTM including: recruitment and drop-out rates, adherence to SOPs, number failing to meet target or progressing and adverse events. The CI will maintain day to day responsibility for the trial with the CTM to ensure that the trial is conducted, recorded and reported in accordance with the protocol, GCP [118] guidelines and SOPs.

The DMC will be comprised of the following individuals in accordance with MRC GCP guidelines: 1) a DMC Chair, 2); Dr Chris Rogers an independent trials statistician, 3) two additional glaucoma or ophthalmic trials specialists. The Priment CTU will advise about potential independent expert trial statisticians to be approached once funding is confirmed. The DMC will meet at least annually or more often if appropriate, timed to report to the TSC. During recruitment, interim reports will be supplied to the DMC, together with any

analyses it requests. The safety, rights and well-being of the Trial Participants being paramount. DMC terms of reference will be available on request.

14 PPI and Lay Advisory Group ~ Service User Input

Glaucoma patients and relatives from the Cochrane Eyes and Vision Group Consumer Panel form our independent Lay Advisory Group (LAG). Consultation on trial design, choice of outcome measures, recruitment and treatment acceptability has taken place by email and through online discussions via the Facebook social-networking site (Group: "Public Eye - LIGHT Trial Discussion Forum"). All agreed with the importance of the fundamental question and applauded the use of HRQL and cost effectiveness as the primary outcome. We have incorporated their suggestions; e.g. requests to monitor all symptoms in detail, a safety concern about "rapid loss of pressure control" after LT and more explanation of the relationship between drops and surgical failure. We have recruited an "Expert Patient" with treated glaucoma who has reviewed and commented upon the study protocol as a service user member for the TSC and another service user representative from the International Glaucoma Association will be invited to join.

The LAG will contribute to the development of tailored information leaflets and consent forms with further consultation with service user groups and via the Friends of Moorfields Charity. We have a strong letter of support for the study from the IGA, an international glaucoma patients advice and advocacy organization. A survey of 100 new patients attending Moorfields to assess acceptability of an invitation to participate in such a trial had 70% positive response. As required by the NHS Constitution, in line with INVOLVE national guidelines and in accordance UKCRC policy we will publicise widely our trial and its results to patients, e.g. via NHS Choices and patient advocate groups (e.g. IGA) and seek to publish the findings in open access media.

15 Direct Access to Source Data/Documents

The investigators and hosting institutions will permit trial-related monitoring, audits, REC review, and regulatory inspections, providing direct access to source data/documents. Trial participants are informed of this during the informed consent discussion. Participants will consent to provide access to their medical notes.

16 Ethics and regulatory requirements

Neither drops nor laser treatment are cause more than minor discomfort or carry significant risk of visual loss due to treatment.

As is standard in normal clinical practice, careful screening questions will identify participants with systemic disease that might put them at risk from a given medicine and this would be excluded from that participants tailored treatment escalation algorithm.

All participants will be competent adults with no recruitment from vulnerable patient groups.

The sponsor will ensure that the trial protocol, patient information sheet, consent form, GP letter and submitted supporting documents are approved by the appropriate regulatory body (MHRA in UK) and a main research ethics committee, prior to any patient recruitment. The protocol and all agreed substantial protocol amendments, will be documented and submitted for ethical and regulatory approval prior to implementation.

Before sites can enrol patients into the trial, the Principal Investigator or designee will apply for Site Specific Assessment from Trust Research & Development (R&D) and be granted written NHS R&D approval. It is the responsibility of the Principal Investigator at each site to ensure that all subsequent amendments gain the necessary approval. This does not affect the individual clinician's responsibility to take immediate action if thought necessary to protect the health and interest of individual patients (see section 9.4.6 for reporting urgent safety measures).

In this study both treatment pathways to be assessed ('Laser-1st' and 'Drops-1st') are already in use and form part of current, available clinical practice, although selective laser trabeculoplasty is not routinely offered in most NHS clinics. Both pathways have well understood treatment side effect profiles and it is therefore possible to give clear advice about the risks and benefits of both treatment arms.

Within 90 days after the end of the trial, the CI and sponsor will ensure that the main REC and the MHRA are notified that the trial has finished. If the trial is terminated prematurely, those reports will be made within 15 days after the end of the trial.

The CI will supply a summary report of the clinical trial to the MHRA and main REC within 1 year after the end of the trial.

17 Monitoring plan for the trial

The trial will be monitored according to the monitoring plan agreed by the sponsor, based on the self-monitoring risk assessment template. This will include source data verification and trial conduct monitoring (e.g. of consent records) by an independent monitor by random sampling. It will be the responsibility of the CI to ensure that the sponsor's self-monitoring template is completed throughout the trial every six months and submitted to the sponsor at the regularity determined by the sponsor's risk assessment of the trial (every 6 months). It is the responsibility of the CI to determine the monitoring risk assessment and explain the rationale.

The PI at each site will also be required to complete a self-monitoring template and return the form at the same frequency, to the CI and sponsor in parallel for review. It is the CI's responsibility to ensure that any findings identified in a PI's monitoring report are acted upon in a timely manner and any violations of GCP or the protocol reported to the sponsor immediately. Any urgent safety measures at either the CI or a PI site must be reported by that site Investigator within 3 days, as per UK Regulations. The trial manager will go on site to check that the PI self-monitoring reports were completed accurately (AUDIT) and ensuring that appropriate corrective and preventive actions are carried out.

18 Finance

This trial is fully funded by the National Institute of Health Research Health Technology Assessment Panel (NIHR HTA), contactable via:

Alexa Cross (Programme Manager (Monitoring))

NETSCC - Health Technology Assessment,

Alpha House, University of Southampton Science Park, Southampton SO16 7NS

Tel: 023 8059 5594 (direct); Fax: 023 8059 5639 Email: A.Cross@soton.ac.uk

19 Insurance

Moorfields Eye Hospital NHS Foundation Trust ("Moorfields") holds insurance to cover participants for injury caused by their participation in the clinical trial. Participants may be able to claim compensation if they can prove that Moorfields has been negligent. This does not affect the participant's right to seek compensation via the non-negligence route.

Participants may also be able to claim compensation for injury caused by participation in this clinical trial without the need to prove negligence on the part of Moorfields or another party. Participants who sustain injury and wish to make a claim for compensation should do so in writing in the first instance to the Chief Investigator, who will pass the claim to the Sponsor's Insurers, via the Sponsor's office.

Hospitals selected to participate in this clinical trial shall provide clinical negligence insurance cover for harm caused by their employees and a copy of the relevant insurance policy or summary shall be provided to the sponsor, upon request.

The SLT laser to be used in the trial will also independently covered by the standard indemnity arrangements to cover the malfunction and breakdown of the device.

20 Statement of compliance

The trial will be conducted in compliance with the protocol, GCP, Moorfields' and PRIMENT (UCL) SOPs and the applicable regulatory requirements.

21 Greenhouse Gas Emissions

We shall maximise use of teleconferencing and schedule all TSC meetings to coincide with existing conferences, to minimise emissions. The SLT Laser-1st treatment pathway itself has the potential to reduce substantially total patient visits and thereby reduce travel and CO_2 emissions.

22 Protocol Training Log

Trial Title: LiGHT Trial

Sponsor ID N0:

Principle Investigator: Gus Gazzard

Trial N0:

Site name: Moorfields Eye Hospital NHS Foundation Trust

	Name of Staff (Capital letters):	Job Title & Department:	Training Date	I confirm that I understand & agree to work to this SOP. SIGNATURE:	Name of Trainer (Capital letters):	Signature	Date

	Name of Staff (Capital letters):	Job Title & Department:	Training Date	I confirm that I understand & agree to work to this SOP. SIGNATURE:	Name of Trainer (Capital letters):	Signature	Date

23 PI’s Log of Protocol &/or GCP Deviations/Violations/Potential serious breaches/Serious breaches/Urgent safety measures

**Is this a**<br>1. Deviation<br>2. Violation<br>3. “Potential Serious breach”<br>4. “Serious breach”<br>5. “Urgent Safety measure”<br>according to the 2 SI definitions below	**Date**<br>**1.** the event took place<br>**2**. the PI became aware of the event	**Corrective Actions**	**Preventative Actions**<br>(e.g. protocol amended, trial halted)	**Date**<br>the event was notified **by the PI to** the sponsor<br>*(if either SI definition are below met)*	**Date**<br>urgent safety measure was **reported by the PI** to the **MHRA and MREC**

1. Definition of "Serious Breach" according to Regulation 29A (SI 2006/1928)

(2) For the purposes of this regulation, a "**serious breach**" is a breach which is likely to effect to a significant degree –

(a) the safety or physical or mental integrity of the subjects of the trial; or (b) the scientific value of the trial".

2. Definition of "Potential serious breach": A breach which is investigated as a breach potentially meeting the definition of "serious breach" above.

3. Definition of "Urgent safety measures" according to Regulation 30 (SI 2004/1031).

The sponsor and investigator may take appropriate '**urgent safety measures'** in order to protect the subjects of a clinical trial against any immediate hazard to their health or safety. The sponsor shall immediately and in any event no later than 3 days from the date the measures are taken, give written notice to the licensing authority and the relevant ethics committee of the measures taken and the circumstances giving rise to those measures.

Regulation 30 of the Medicines for Human Use (Clinical Trials) Regulations 2004(SI 2004/1031) was amended by (SI 2009/1164):

For paragraph 2 of regulation 30 of the Medicines for Human Use (Clinical Trials) Regulations 2004 (urgent safety measures) (**a**), substitute the following paragraphs—

"(2) If measures are taken pursuant to paragraph (1), the sponsor shall—

(a) where paragraph (3) applies, as soon as possible; and

(b) in any other case, immediately, and in any event no later than 3 days from the date the measures are taken, give written notice to the licensing authority and the relevant ethics committee of the measures taken and the circumstances giving rise to those measures.

(3) This paragraph applies for any period during which a disease :

(a) is pandemic; and (b) is a serious risk to human health or potentially a serious risk to human health."

24 References

Reference List

1. National Institute for Health and Clinical Excellence, NICE L. NICE: Guidance on Glaucoma: Diagnosis and management of chronic open angle glaucoma and ocular hypertension. DoH 2010;www.nice.org.uk/CG85fullguideline.

2. Quigley HA, Broman AT. The number of people with glaucoma worldwide in 2010 and 2020. Br J Ophthalmol 2006;90:262-7.

3. Minassian DC, Reidy A, Coffey M, Minassian A. Utility of predictive equations for estimating the prevalence and incidence of primary open angle glaucoma in the UK. Br J Ophthalmol 2000;84:1159-61.

4. Bunce C, Wormald R. Leading causes of certification for blindness and partial sight in England & Wales. BMC Public Health 2006;6:58.

5. Haymes SA, LeBlanc RP, Nicolela MT, et al. Risk of Falls and Motor Vehicle Collisions in Glaucoma. Investigative Ophthalmology Visual Science 2007;48:1149-55.

6. Haymes SA, LeBlanc RP, Nicolela MT, et al. Glaucoma and on-road driving performance. Invest Ophthalmol Vis Sci 2008;49:3035-41.

7. Parrish RK, Gedde SJ, Scott IU, et al. Visual function and quality of life among patients with glaucoma. Arch Ophthalmol 1997;115:1447-55.

8. Nelson P, Aspinall P, Papasouliotis O, et al. Quality of life in glaucoma and its relationship with visual function. J Glaucoma 2003;12:139-50.

9. Gutierrez P, Wilson R, Johnson C, et al. Influence of Glaucomatous Visual Field Loss on Health-Related Quality of Life. Arch Ophthalmol 1997;115:777-84.

10. Nelson P, Aspinall P, O'Brien C. Patients' perception of visual impairment in glaucoma: a pilot study. Br J Ophthalmol 1999;83:546-52.

11. Higginbotham EJ, Gordon MO, Beiser JA, et al. The Ocular Hypertension Treatment Study: Topical Medication Delays or Prevents Primary Open-angle Glaucoma in African American Individuals. Arch Ophthalmol 2004;122:813-20.

12. Kass MA, Heuer DK, Higginbotham EJ, et al. The Ocular Hypertension Treatment Study: a randomized trial determines that topical ocular hypotensive medication delays or prevents the onset of primary open-angle glaucoma. Arch Ophthalmol 2002;120:701-13.

13. LOCSU. National Eye Health Epidemiological Model (NEHEM).Available at: http://www.eyehealthmodel.org.uk/MainApplication/Default.aspx# .

14. Gordon MO, Beiser JA, Brandt JD, et al. The Ocular Hypertension Treatment Study: Baseline factors that predict the onset of primary open-angle glaucoma. Arch Ophthalmol 2002;120:714-20.

15. Taylor HR, Crowston JG, Centre for Eye Research Australia. The Economic Impact of Primary Open Angle Glaucoma - *A Dynamic Economic Model*. Centre for Eye Research Australia 2008.

16. Kobelt G, Jonsson L. Modeling cost of treatment with new topical treatments for glaucoma. Results from France and the UK. Int J Technol Assess Health Care 1999;15:207-19.

17. Lee R, Hutnik CM. Projected cost comparison of selective laser trabeculoplasty versus glaucoma medication in the Ontario Health Plan. Can J Ophthalmol 2006;41:449-56.

18. Lee PP, Walt JG, Doyle JJ, et al. A multicenter, retrospective pilot study of resource use and costs associated with severity of disease in glaucoma. Arch Ophthalmol 2006;124:12-9.

19. Rein DB, Wittenborn JS, Lee PP, et al. The cost-effectiveness of routine office-based identification and subsequent medical treatment of primary open-angle glaucoma in the United States. Ophthalmology 2009;116:823-32.

20. Cantor LB, Katz LJ, Cheng JW, et al. Economic evaluation of medication, laser trabeculoplasty and filtering surgeries in treating patients with glaucoma in the US. Curr Med Res Opin 2008;24:2905-18.

21. Taylor HR. Glaucoma: where to now? Ophthalmology 2009;116:821-2.

22. Burr JM, Mowatt G, Hernandez R, et al. The clinical effectiveness and cost-effectiveness of screening for open angle glaucoma: a systematic review and economic evaluation. Health Technol Assess 2007;11:iii-x, 1.

23. National Institute for Health and Clinical Excellence, NICE L. NICE: Guidance on Glaucoma: Diagnosis and management of chronic open angle glaucoma and ocular hypertension - Costing report . DoH 2010;www.nice.org.uk/CG85fullguideline.

24. Owen CG, Carey IM, de WS, et al. The epidemiology of medical treatment for glaucoma and ocular hypertension in the United Kingdom: 1994 to 2003. Br J Ophthalmol 2006;90:861-8.

25. Collaborative Normal-Tension Glaucoma Study Group. Comparison of glaucomatous progression between untreated patients with normal-tension glaucoma and patients with therapeutically reduced intraocular pressures. Collaborative Normal-Tension Glaucoma Study Group. Am J Ophthalmol 1998;126:487-97.

26. Drance SM. The collaborative normal-tension glaucoma study and some of its lessons. Canadian Journal of Ophthalmology 1999;34:1-6.

27. Lichter PR, Musch DC, Gillespie BW, et al. Interim clinical outcomes in the Collaborative Initial Glaucoma Treatment Study comparing initial treatment randomized to medications or surgery. Ophthalmology 2001;108:1943-53.

28. Gaasterland DE, Ederer F, Beck A, et al. The Advanced Glaucoma Intervention Study (AGIS): 7. The relationship between control of intraocular pressure and visual field deterioration. Am J Ophthalmol 2000;130:429-40.

29. Leske MC, Heijl A, Hussein M, et al. Factors for Glaucoma Progression and the Effect of Treatment: The Early Manifest Glaucoma Trial. Arch Ophthalmol 2003;121:48-56.

30. Barton K. Bleb dysesthesia. J Glaucoma 2003;12:281-4.

31. Budenz DL, Hoffman K, Zacchei A. Glaucoma filtering bleb dysesthesia. Am J Ophthalmol 2001;131:626-30.

32. Kirwan JF, Nightingale JA, Bunce C, Wormald R. Beta blockers for glaucoma and excess risk of airways obstruction: population based cohort study. BMJ 2002;325:1396-7.

33. Fukuchi T, Wakai K, Suda K, et al. Incidence, severity and factors related to drug-induced keratoepitheliopathy with glaucoma medications. Clin Ophthalmol 2010;4:203-9.

34. Alm A, Grierson I, Shields MB. Side effects associated with prostaglandin analog therapy. Surv Ophthalmol 2008;53 Suppl1:S93-105.

35. Balkrishnan R, Bond JB, Byerly WG, et al. Medication-related predictors of health-related quality of life in glaucoma patients enrolled in a medicare health maintenance organization. Am J Geriatr Pharmacother 2003;1:75-81.

36. Rahman MQ, Abeysinghe SS, Kelly S, et al. Persistence of glaucoma medical therapy in the Glasgow Glaucoma Database. Br J Ophthalmol.

37. Rahman MQ, Montgomery DMI, Lazaridou MN. Surveillance of glaucoma medical therapy in a Glasgow teaching hospital: 26 years experience. Br J Ophthalmol 2009;93:1572-5.

38. Nordstrom BL, Friedman DS, Mozaffari E, et al. Persistence and adherence with topical glaucoma therapy. Am J Ophthalmol 2005;140:598-606.

39. Robin AL, Novack GD, Covert DW, et al. Adherence in glaucoma: objective measurements of once-daily and adjunctive medication use. Am J Ophthalmol 2007;144:533-40.

40. Tsai JC. Medication adherence in glaucoma: approaches for optimizing patient compliance. Current Opinion in Ophthalmology 2006;17:190-5.

41. Tsai JC. A comprehensive perspective on patient adherence to topical glaucoma therapy. Ophthalmology 2009;116:S30-S36.

42. Tsai JC, McClure CA, Ramos SE, et al. Compliance barriers in glaucoma: a systematic classification. Journal of Glaucoma 12(5):393-8, 2003.

43. Sharkawi E, Franks W. Glaucoma and mortality. Ophthalmology 2008;115:213-4.

44. Diggory P, Franks W. Medical treatment of glaucoma--a reappraisal of the risks. Br J Ophthalmol 1996;80:85-9.

45. Broadway DC, Grierson I, O'Brien C, Hitchings RA. Adverse effects of topical antiglaucoma medication. II. The outcome of filtration surgery. Arch Ophthalmol 1994;112:1446-54.

46. Broadway DC, Grierson I, O'Brien C, Hitchings RA. Adverse effects of topical antiglaucoma medication. I. The conjunctival cell profile. Arch Ophthalmol 1994;112:1437-45.

47. Sherwood MB, Grierson I, Millar L, Hitchings RA. Long-term morphologic effects of antiglaucoma drugs on the conjunctiva and Tenon's capsule in glaucomatous patients. Ophthalmology 1989;96:327-35.

48. Broadway DC, Chang LP. Trabeculectomy, risk factors for failure and the preoperative state of the conjunctiva. J Glaucoma 2001;10:237-49.

49. Morris DA, Peracha MO, Shin DH, et al. Risk factors for early filtration failure requiring suture release after primary glaucoma triple procedure with adjunctive mitomycin. Arch Ophthalmol 1999;117:1149-54.

50. Baudouin C. Mechanisms of failure in glaucoma filtering surgery: a consequence of antiglaucomatous drugs? Int J Clin Pharmacol Res 1996;16:29-41.

51. Kramer TR, Noecker RJ. Comparison of the morphologic changes after selective laser trabeculoplasty and argon laser trabeculoplasty in human eye bank eyes. Ophthalmology 2001;108:773-9.

52. Latina MA, Sibayan SA, Shin DH, et al. Q-switched 532-nm Nd:YAG laser trabeculoplasty (selective laser trabeculoplasty): a multicenter, pilot, clinical study. Ophthalmology 1998;105:2082-8.

53. Latina MA, Tumbocon JA. Selective laser trabeculoplasty: a new treatment option for open angle glaucoma. Current Opinion in Ophthalmology 2002;13:94-6.

54. Latina MA, Gulati V. Selective laser trabeculoplasty: stimulating the meshwork to mend its ways. International Ophthalmology Clinics 2004;44:93-103.

55. Latina MA, Park C. Selective targeting of trabecular meshwork cells: in vitro studies of pulsed and CW laser interactions. Exp Eye Res 1995;60:359-71.

56. Shazly TA, Latina MA. Intraocular Pressure Response to Selective Laser Trabeculoplasty in the First Treated Eye vs the Fellow Eye. Arch Ophthalmol 2011;129:699-702.

57. Acott TS, Samples JR, Bradley JM, et al. Trabecular repopulation by anterior trabecular meshwork cells after laser trabeculoplasty. Am J Ophthalmol 1989;107:1-6.

58. Bylsma SS, Samples JR, Acott TS, Van Buskirk EM. Trabecular cell division after argon laser trabeculoplasty. Arch Ophthalmol 1988;106:544-7.

59. Alvarado JA, Katz LJ, Trivedi S, Shifera AS. Monocyte modulation of aqueous outflow and recruitment to the trabecular meshwork following selective laser trabeculoplasty. Arch Ophthalmol 2010;128:731-7.

60. Glaucoma Laser Trial. The Glaucoma Laser Trial (GLT) and glaucoma laser trial follow- up study: 7. Results. Glaucoma Laser Trial Research Group. Am J Ophthalmol 1995;120:718-31.

61. Baez KA, Spaeth GL. Argon laser trabeculoplasty controls one third of patients with progressive, uncontrolled open-angle glaucoma for five years. Transactions of the American Ophthalmological Society 1991;89:47-56.

62. Damji KF, Shah KC, Rock WJ, et al. Selective laser trabeculoplasty v argon laser trabeculoplasty: a prospective randomised clinical trial. Br J Ophthalmol 1999;83:718-22.

63. Damji KF, Bovell AM, Hodge WG, et al. Selective laser trabeculoplasty versus argon laser trabeculoplasty: results from a 1-year randomised clinical trial. Br J Ophthalmol 2006;90:1490-4.

64. Juzych MS, Chopra V, Banitt MR, et al. Comparison of long-term outcomes of selective laser trabeculoplasty versus argon laser trabeculoplasty in open-angle glaucoma. Ophthalmology 2004;111:1853-9.

65. Hong BK, Winer JC, Martone JF, et al. Repeat selective laser trabeculoplasty. J Glaucoma 2009;18:180-3.

66. McIlraith I, Strasfeld M, Colev G, Hutnik CM. Selective laser trabeculoplasty as initial and adjunctive treatment for open-angle glaucoma. J Glaucoma 2006;15:124-30.

67. Nagar M, Ogunyomade A, O'Brart DPS, et al. A randomised, prospective study comparing selective laser trabeculoplasty with latanoprost for the control of intraocular pressure in ocular hypertension and open angle glaucoma. Br J Ophthalmol 2005;89:1413-7.

68. Melamed S, Ben Simon GJ, Levkovitch-Verbin H. Selective laser trabeculoplasty as primary treatment for open-angle glaucoma: a prospective, nonrandomized pilot study. Arch Ophthalmol 2003;121:957-60.

69. Francis BA, Ianchulev T, Schofield JK, Minckler DS. Selective laser trabeculoplasty as a replacement for medical therapy in open-angle glaucoma. Am J Ophthalmol 2005;140:524-5.

70. Rolim de MC, Paranhos A, Jr., Wormald R. Laser trabeculoplasty for open angle glaucoma. Cochrane Database Syst Rev 2007;CD003919.

71. Francis BA, Ianchulev S, Schofield JK, Minckler DA. Selective Laser Trabeculoplasty as a Replacement for Medical Therapy in Open-Angle Glaucoma. Am J Ophthalmol 2005;140:524-5.

72. Sharma A, Jofre-Bonet M, Panca M, et al. Hospital-based glaucoma clinics: what are the costs to patients? Eye 2009;24:999-1005.

73. European Glaucoma Society. Terminology and Guidelines for Glaucoma 3rd Ed. EGS 2008;http://www.eugs.org/eng/EGS_guidelines.asp.

74. EuroQol. EuroQol--a new facility for the measurement of health-related quality of life. The EuroQol Group. Health Policy 1990;16:199-208.

75. Cioffi GA, Latina MA, Schwartz GF. Argon versus selective laser trabeculoplasty. J Glaucoma 2004;13:174-7.

76. Damji KF, Behki R, Wang L. Canadian perspectives in glaucoma management: setting target intraocular pressure range. Can J Ophthalmol 2003;38:189-97.

77. Leske MC, Heijl A, Hyman L, Bengtsson B. Early Manifest Glaucoma Trial: design and baseline data. Ophthalmology 1999;106:2144-53.

78. Tan GS, Wong TY, Fong CW, Aung T. Diabetes, metabolic abnormalities, and glaucoma. Arch Ophthalmol 2009;127:1354-61.

79. Lin HC, Chien CW, Hu CC, Ho JD. Comparison of Comorbid Conditions between Open-Angle Glaucoma Patients and a Control Cohort A Case-Control Study. Ophthalmology 2010.

80. Imai K, Hamaguchi M, Mori K, et al. Metabolic syndrome as a risk factor for high-ocular tension. Int J Obes (Lond) 2010.

81. Hewitt AW, Wu J, Green CM, et al. Systemic disease associations of familial and sporadic glaucoma: the Glaucoma Inheritance Study in Tasmania. Acta Ophthalmol 2010;88:70-4.

82. Mills T, Law SK, Walt J, et al. Quality of life in glaucoma and three other chronic diseases: a systematic literature review. Drugs Aging 2009;26:933-50.

83. Burr J, Kilonzo M, Vale L, Ryan M. Developing a Preference-Based Glaucoma Utility Index Using a Discrete Choice Experiment. [Article]. Optometry & Vision Science 2007;84:E797-E809.

84. Lee BL, Gutierrez P, Gordon M, et al. The Glaucoma Symptom Scale. A brief index of glaucoma-specific symptoms. Arch Ophthalmol 1998;116:861-6.

85. American Academy of Ophthalmology. Primary Open-Angle Glaucoma: Preferred Practice Pattern. AAO 2005.

86. SEAGIG. South East Asia Gluacoma Interest Group: Asia Pacific Glaucoma Guidelines. SEAGIG 2003.

87. Jampel HD. Target pressure in glaucoma therapy. J Glaucoma 1997;6:133-8.

88. Palmberg P. How clinical trial results are changing our thinking about target pressures. Current Opinion in Ophthalmology 2002;13:85-8.

89. Realini T, Fechtner RD. Target intraocular pressure in glaucoma management. Ophthalmology Clinics of North America 2000;13:407-15.

90. Heijl A, Leske MC, Bengtsson B, et al. Reduction of intraocular pressure and glaucoma progression: results from the Early Manifest Glaucoma Trial. Arch Ophthalmol 2002;120:1268-79.

91. Collaborative Normal-Tension Glaucoma Study Group. The effectiveness of intraocular pressure reduction in the treatment of normal-tension glaucoma. Collaborative Normal-Tension Glaucoma Study Group. Am J Ophthalmol 1998;126:498-505.

92. Mills RP, Budenz DL, Lee PP, et al. Categorizing the stage of glaucoma from pre-diagnosis to end-stage disease. Am J Ophthalmol 2006;141:24-30.

93. Canadian Glaucoma Study. Canadian Glaucoma Study: 1. Study design, baseline characteristics, and preliminary analyses. Can J Ophthalmol 2006;41:566-75.

94. Hahn SR, Friedman DS, Quigley HA, et al. Effect of Patient-Centered Communication Training on Discussion and Detection of Nonadherence in Glaucoma. Ophthalmology 2010.

95. Friedman DS, Hahn SR, Gelb L, et al. Doctor-patient communication, health-related beliefs, and adherence in glaucoma results from the Glaucoma Adherence and Persistency Study. Ophthalmology 2008;115:1320-7, 1327.

96. Friedman DS. Introduction: new insights on enhancing adherence to topical glaucoma medications. Ophthalmology 2009;116:S29.

97. Chauhan BC, Hutchison DM, Artes PH, et al. Optic Disc Progression in Glaucoma: Comparison of Confocal Scanning Laser Tomography to Optic Disc Photographs in a Prospective Study. Investigative Ophthalmology Visual Science 2009;50:1682-91.

98. Strouthidis NG, Scott A, Peter NM, Garway-Heath DF. Optic disc and visual field progression in ocular hypertensive subjects: detection rates, specificity, and agreement. Investigative Ophthalmology & Visual Science 2006;47:2904-10.

99. Patel A, Rendu A, Moran P, et al. A comparison of two methods of collecting economic data in primary care. Family Practice 2005;22:323-7.

100. Beecham J, Knapp M. Costing psychiatric interventions. In: Thornicroft G, ed. Measuring Mental Health Needs. London: Gaskel; 2001.

101. Dolan P. Modeling valuations for EuroQol health states. Med Care 1997;35:1095-108.

102. National Institute for Health and Clinical Excellence. NICE: Guide to the methods of technology appraisal. NICE Guidance 2008.

103. Briggs AH, Wonderling DE, Mooney CZ. Pulling cost-effectiveness analysis up by its bootstraps: a non-parametric approach to confidence interval estimation. Health Econ 1997;6:327-40.

104. Crabb DP, Fitzke FW, Hitchings RA, Viswanathan AC. A practical approach to measuring the visual field component of fitness to drive. Br J Ophthalmol 2004;88:1191-6.

105. Crabb DP, Viswanathan AC. Integrated visual fields: a new approach to measuring the binocular field of view and visual disability. Graefes Archive for Clinical & Experimental Ophthalmology 2005;243:210-6.

106. Traverso CE, Walt JG, Kelly SP, et al. Direct costs of glaucoma and severity of the disease: a multinational long term study of resource utilisation in Europe. Br J Ophthalmol 2005;89:1245-9.

107. Briggs A, Sculpher M, Claxton K. Decision Modelling for Health Economic Evaluation. OUP 2006.

108. Walt JGM, Rendas-Baum RM, Kosinski MM, Patel VP. Psychometric Evaluation of the Glaucoma Symptom Identifier. J Glaucoma 2011;20:148-59.

109. Hirneiss C., Kampik A, Neubauer AS. Value-based medicine bei Glaukom. Der Ophthalmologe 2010;107:223-7.

110. Skalicky S, Goldberg I. Depression and Quality of Life in Patients With Glaucoma: A Cross-sectional Analysis Using the Geriatric Depression Scale-15, Assessment of Function Related to Vision, and the Glaucoma Quality of Life-15. [Article]. J Glaucoma 2008;17:546-51.

111. Goldberg. I, Clement CIM, Chiang THP, et al. Assessing Quality of Life in Patients With Glaucoma Using the Glaucoma Quality of Life-15 (GQL-15) Questionnaire. J Glaucoma 2009;18:6-12.

112. van Gestel A, Webers CAB, Beckers HJM, et al. The relationship between visual field loss in glaucoma and health-related quality-of-life. Eye 2010;24:1759-69.

113. Skalicky SE, Goldberg I, McCluskey P. Ocular Surface Disease and Quality of Life in Patients With Glaucoma. American Journal of Ophthalmology 153[1], 1-9. 1-1-2012.
Ref Type: Abstract

114. Azuara-Blanco A, Burr JM. 09PRT/4779: The effectiveness of early lens extraction with intraocular lens implantation for the treatment of primary angle-closure glaucoma: a randomised controlled trial (EAGLE) (ISRCTN 44464607). Lancet 2010.

115. Walters SJ, Brazier JE. Comparison of the minimally important difference for two health state utility measures: EQ-5D and SF-6D. Qual Life Res 2005;14:1523-32.

116. Aspinall PA, Johnson ZK, Azuara-Blanco A, et al. Evaluation of Quality of Life and Priorities of Patients with Glaucoma. Investigative Ophthalmology Visual Science 2008;49:1907-15.

117. Schulz KF, Altman DG, Moher D. CONSORT 2010 statement: updated guidelines for reporting parallel group randomised trials. BMJ 2010;340:c332.

118. MHRA. Good Clinical Practice. MHRA 2010.

25 Appendices Contents

Appendix 1: Overall Trial Design Flow-chart

Appendix 2: Recruitment Rate Prediction

Appendix 3: Stratification of Patients by Severity of Their Disease

Appendix 4: Frequency of Follow-Up According to Severity of Disease

Appendix 5: Details of Treatment

(1) Laser-1st

(2) Details of Treatment ~ Medicine-1st

(3) Details of Surgical Treatment, if other treatments fail

Appendix 6: Changing Treatment

(1) Definition of 'Failure to Meet Target'

(2) Process for Treatment-Target Reassessment

(3) Treatment escalation to glaucoma surgery

Appendix 7: Detection of Progressive Glaucoma Damage

(1) Visual Field progression

(2) Optic Disc progression

Appendix 8: How to set the Treatment Target IOP & Flow-chart

Appendix 9a: If Target is not met: when to escalate treatment & when to reassess Target for OHT

Appendix 9b: If Target is not met: when to escalate treatment & when to reassess Target for OAG

Appendix 10: How to Escalate Treatment

Appendix 11: Questionnaires Delivery and Follow-up

Appendix 12: Side Effects : Standardised History Taking

Appendix 13: Gantt Chart

Appendix 14: Video Script

26 Appendix 1 ~ Trial Design Flow-chart

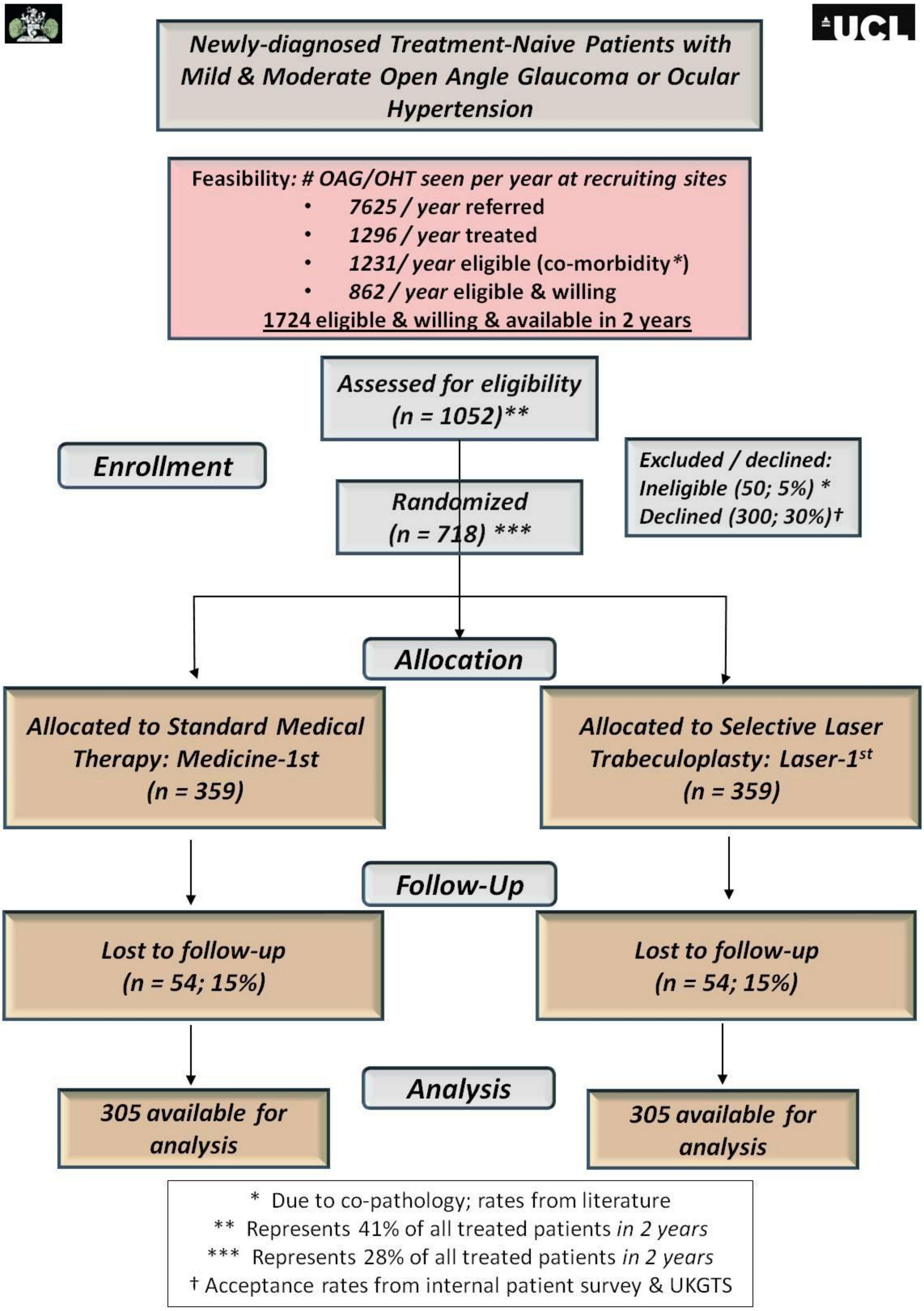

27 Appendix 2 ~ Predicted recruitment rate

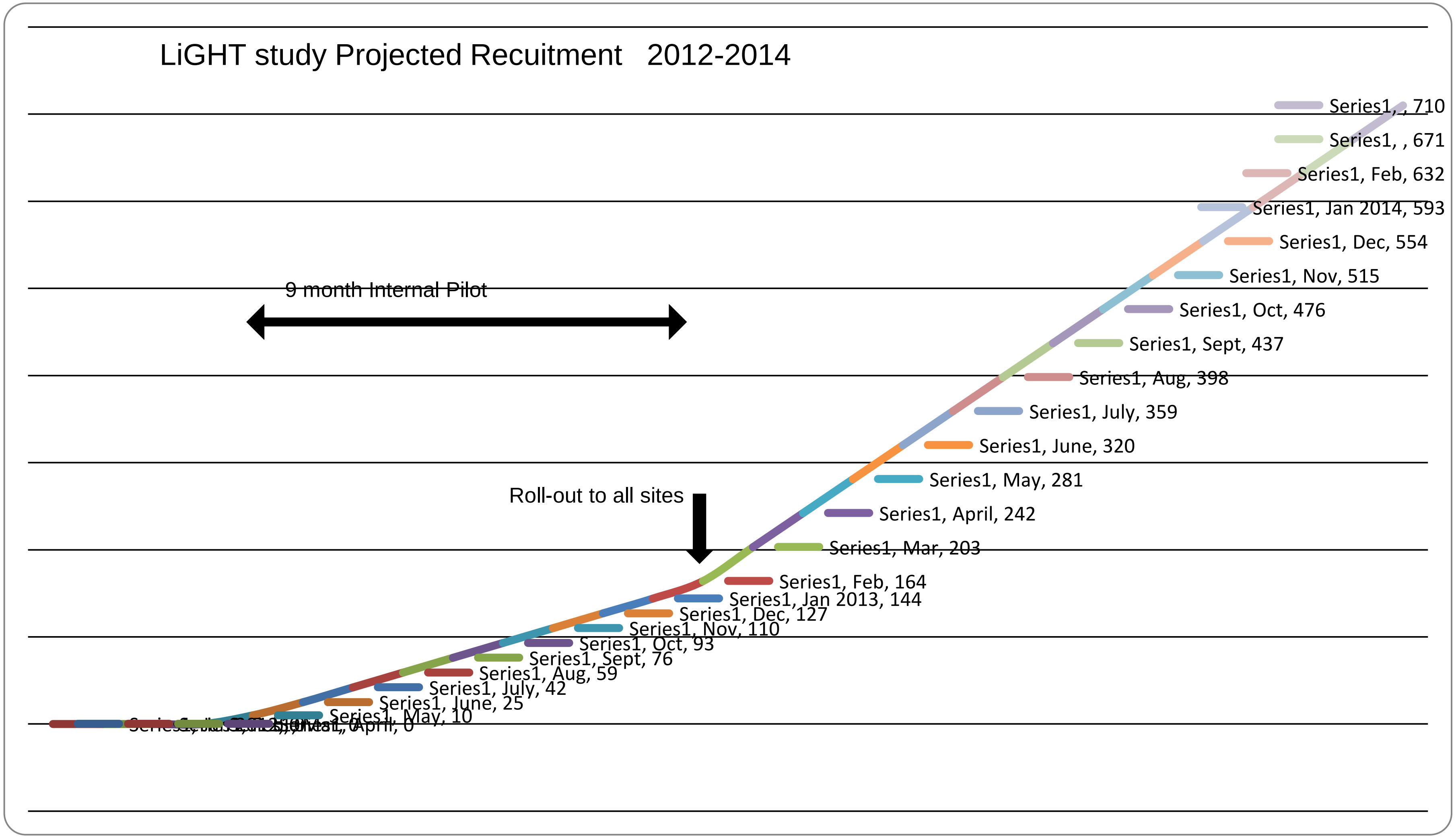

28 Appendix 3 ~ Stratification of Patients by Severity of Their Disease

**Severity**	**Definition for Treatment Target IOP**				
	Optic Nerve Appearance (Vertical Cup Disc Ratio)		Mean deviation (MD)		Central Scotoma on HVF* (Central 10 deg)
**OHT**	Healthy Optic disc		Any		No GON related VFL
**Mild OAG**	GON	+	> -6dB	+	None
**Moderate OAG**	GON	+	-6dB < & < -12dB	*or*	At least 1 central 5º point <15d but none <0dB & only 1 hemifield with central point <15dB[92]
**Severe OAG**	GON	+	< -12dB	*or*	Any central 5º point with sensitivity <0dB<br>Both hemifields contain point(s) <15dB within 5º of fixation

Severity criteria for setting Treatment Target IOP from the "Canadian Target IOP Workshop" [76] (with central field criteria defined according to Mills) [92]. (See also notes on next page)

Stratification by Disease Severity: Notes

Severity stratification will be calculated throughout the trial and influence follow-up frequency but any change in severity category will *not* affect the target IOP after it is set at the baseline visit. Target IOP is altered only in response to progression at target IOP or a lack of progression while above target on MMT.

Non-glaucoma reasons for an increased MD that would not otherwise lead to exclusion from the trial (such as a paramacular scar with good vision and no prior surgery) will be considered on a case-by-case basis and requires discussion with the CI before inclusion in the study.

The effects of cataract on MD require special mention. The presence of visually significant *and* symptomatic cataract in patients who desire surgery will exclude them from the study. Some, however, will have lens opacities that reduce MD but do not affect VA enough for the patient to want surgery. In this case we shall proceed with the MD derived severity categorisation in the knowledge that there is a theoretical risk of slightly more intense intervention in this case. If the decision to proceed to glaucoma surgery arises then this will be taken into account at that time by the treating clinician.

Any non-glaucoma causes for increases in MD during the course of the trial (eg BRVO), could lead to an incorrect severity category and the generation of incorrect follow-up intervals. These will be detected clinically and lead to an over-ruling of the Treatment Algorithm as deemed clinically appropriate for that patient.

29 Appendix 4 ~ Frequency of Follow-Up According to Severity of Disease (Stable Patients)

Disease Severity Category	Routine follow-up intervals, in months, following Initial Treatment**							
	1st visit	2nd visit	3rd	4th	5th	6th	7th	8th...
**OHT**	1st Rx	2*	4	6	12	12	12	12 ...
**Mild OAG**	1st Rx	2*	4	6	6	12	12	12 ...
**Moderate OAG**	1st Rx	2*	4	6	6	6	6	6 ...
**Severe OAG**	1st Rx	1-2*	4	6	6	6	6	6 ...
**Time from baseline (months)**	0	1-2	6	12	.... Patient-Specific			

Routine follow-up schedule by category of disease severity for *stable* patients

- This is the planned routine schedule of appointments for subjects who remain at Target without Progression or treatment change and have no adverse effects requiring earlier assessment.
- These comply with NICE Guidance [1] (www.nice.org.uk/CG85fullguideline), though intervals are more closely specified.
- Additional VF tests will be permissible at any visit if necessary to confirm possible progression, as per usual clinical practice.
- ** The only difference in standard follow-up schedules between Medicine-1st and Laser-1st pathways is that the first follow-up (*) occurs at 2 weeks for laser-1st , not 1-2 months, except for Severe disease for which first review is at 4-8 weeks, on safety criteria.
- Variation in follow-up intervals is permitted to accommodate patient choice at +/- 25% of planned interval.

30 Appendix 5 ~ Details of Treatment

30.1 Appendix 5a ~ Trial arm 1: "Laser-1st" Pathway, SLT

Training will be given to all treating surgeons before recruitment and at least one laser treatment observed by the CI. The treating surgeon will be the local PI or a fellowship trained glaucoma specialist eligible to apply for a UK consultant surgeon post or for inclusion on the UK GMC Specialist Register, who has performed at least 25 previous SLT treatments.

SLT will be delivered to 360º of the trabecular meshwork with one 360° re-treatment as the first escalation of treatment if required. Which model of SLT laser may be used is not restricted, the wavelength and spot size are the same. To minimise variation between surgeons standardisation will be achieved by a stringent protocol defining laser settings and technique.

Pre-treatment with Iopidine (0.5% or 1%) at least 15 minutes before laser is mandatory, unless contra-indicated for medical reasons when alternative medications such as oral acetazolamide may be used. If no prophylaxis against IOP spikes is used close post-treatment monitoring of IOP for 2 hours is necessary.

One hundred non-overlapping shots (25 per quadrant) of a preset 3 nanoseconds duration and a preset 400µm spot size will be used, with the laser energy varied from 0.3 to 1.4mJ by the clinician using any laser gonioscopy lens (as long as the appropriate magnification is observed: eg 'Latina' acceptable but 'Magnaview' not). The desired end-point is the production of a few fine "champagne bubbles": larger gas bubbles or TM blanching are not acceptable, and if seen the operator should titrate the power downwards in 0.1mJ increments.

Pigmented TM will require lower energy (from 0.3mJ to 1.2mJ) than non-pigmented and it is advisable to start treatments at 0.4mJ.

The GAT IOP will be measured 1hour after treatment.

After treatment with SLT patients will not be asked to use anti-inflammatory eye-drops *routinely* but *will* be provided with a bottle of topical non-steroidal anti-inflammatory eye-drops for use only if they are in significant discomfort, despite simple oral analgesia such as paracetamol. (This is now standard practice in most units worldwide, *personal communication from Mark Latina).* Topical steroids will not be permitted post-laser. Demonstrations of correct drop technique will be given at baseline and whenever needed thereafter.

Post-operative IOP rises may occur immediately or (rarely) at subsequent assessments. Any rise of IOP >10mmHg *or* that puts the patient at risk of visual loss may be treated at the discretion of the treating ophthalmologist with an earlier recheck of IOP (e.g. at 2 hours, 1 day or 1 week) and/or a short-term course of topical or systemic aqueous

suppressants as necessary. An IOP rise needing medical treatment or an extra visit alone would constitute an adverse event and be independently logged as such.

First post-SLT follow-up is at 2 weeks for IOP check and assessment of potential side-effects. No re-intervention / treatment escalation decisions for non-response will be made at this point – a further follow-up 6 weeks later is to allow time for the full effects of laser to occur. Patients at Target eight weeks after SLT will be subsequently reviewed as per the interval determined by the severity category. Patients not at Target after a single SLT will receive another treatment of 360º (100 spots) at the same energy settings, with re-evaluation after 2 weeks. After *re*-treatment a 6 week follow-up will be given whether at Target or not, *unless* a dangerously high IOP poses a significant risk to vision in the opinion of the treating clinician, in which case earlier follow-up will be allowed to avoid an unsafe delay in medical therapy.

It is possible that an IOP rise following SLT could be severe enough to prevent safe repeat SLT should the Target not be met in future, particularly with more severe GON. In this case Treatment escalation with drops rather than repeating the SLT will be permitted. This requires an algorithm over-ride and thus will be automatically logged and monitored. This may occur as an immediate (but transient) or persistent post-SLT pressure rise. Any immediate IOP rise above 40mmHg despite pre-treatment Iopidine or any rise of over 5mmHg that persists 8 weeks after laser would usually prevent further SLT treatment.

After two SLT treatments the Laser-1st Pathway embarks on medical treatment and follows the Medicine-1st algorithms (below).

If the participant subsequently undergoes drainage surgery which fails in the course of the trial the step-wise *medical* intervention algorithm begins again and further SLT will not be permitted.

30.2 Appendix 5b ~ Trial arm 2: "Medicine-1st" Pathway, Drops

Medical treatment of glaucoma involves several distinct steps that require standardisation: choice of drugs; number of agents permitted; rules for switching between or adding drugs.

International best practice guidelines [73;85;86] advocate changing medication if the target is not reached, with the addition or switching of medication (based on the magnitude of initial response). Surgery is offered once a maximum treatment intensity is reached: "Maximum Medical Therapy (MMT)". This varies between patients but requires definition to minimise inter-surgeon variation.

Post-medical treatment change the IOP will be reviewed at 6-8 weeks.

If treatment in discontinued for any reason (e.g. allergy) the next review visit will be at the discretion the treating clinician depending on severity and other clinical factors but must be *no later* than 8 weeks after the discontinuation.

30.2.1 Maximum Medical Therapy – MMT

MMT is the most intensive combination of drops that a given individual can reasonably, reliably and safely use. MMT varies between patients depending on co-morbidities, side effects and patient-specific compliance factors. While there is surgeon variation in attitudes to poly-pharmacy it is widely accepted that additional medications result in progressively lower percentage reductions in IOP and evidence shows there are profound reductions in compliance with complex dosage schedules.

NICE Guidance [1] recommends offering surgery after only two drugs have failed to control IOP. We shall limit treatment with multiple different medications and define MMT in terms of the maximum number of drugs (3) and dosages per day (5 drops). MMT will often be *less* than this, due to drug intolerance, contra-indications and patient factors.

Patient choice: some patients will decline surgery when offered, even if deteriorating on MMT. In this situation more than 3 medications or 5 dosages will be permitted if treatment escalation is indicated.

All decisions to offer surgery will be consultant-led.

30.2.2 Choice of Agent

No mainstream medications are prohibited but drugs classes for 1st, 2nd, or 3rd line treatment are defined as per NICE [1] and EGS guidance [73]:

1st line: Prostaglandin analogue (PGA)

2nd line: Beta blocker (once in the morning or in a PGA combination)

3rd or 4th line: Topical carbonic anhydrase inhibitor (CAI) or alpha-agonist (alone or in combination with a beta blocker)

Systemic CAIs are only permitted as a temporising measure while awaiting surgery and will not influence treatment escalation. Pilocarpine is not an accepted medication for OAG. All choices are assuming no contra-indications such as heart block, asthma, allergy etc in which case the next line drugs would be considered.

Preservative-free preparations will be permitted where they form part of the treating clinician's usual clinical practice.

Where unilateral PGA use might be undesirable then Beta blocker use will be permitted as initial therapy according to the practice of the treating clinician.

New medications or preparations will likely occur during this study. We shall permit all new formulations to maximise generalisability, but after discussion at the Trial Steering Committee of the appropriate usage within the escalation hierarchy.

30.2.3 To Add or Switch Medicines?

The Incremental Escalation of Treatment protocol defines stepwise increase in treatment. Patients may be *switched* if the pre- and post- treatment IOP difference is no greater than measurement error. If there is a greater reduction but the eye is still not at Target then the next medication may be *added*. Progression of GON when at Target will also trigger a stepwise increase of treatment *and* a lowering of the target (as above).

Switches *within class* are permitted for intolerance to a drop component (e.g. from a preserved to a preservative-free preparation) *or* for lack of efficacy (i.e. the pre- and post-treatment IOP difference is no greater than measurement error) but *only* when that is the treating clinicians usual practice *and* where evidence for a non-responder group is well described in the literature (e.g. switch from latanoprost to bimatoprost is permitted for poor response and bimatoprost to latanoprost for PGA intolerance).

More than one switch within a class is not permitted unless the patient subsequently undergoes failed drainage surgery in the course of the trial at which point the step-wise medical intervention algorithm begins again.

30.2.4 Training and Education in Drop Treatment

Variation in compliance between groups (where patients in both are using drops) is a potential source of bias – particularly since 'compliance' can be affected by many subtle factors including communication skills of the treating physician and the level of pre-treatment education given. Thus an attempt will be made to minimise this by standardising the pre-treatment education using the International Glaucoma Association (IGA) 'compliance kit' as a template. Drop technique will be demonstrated and then observed by the treating clinician when any participant is started on medication for the first time, "compliance-aids" will be offered and standardised 'Ask-Tell-Ask' techniques used to assess and improve compliance.

30.2.5 Other Ophthalmic Medications

Topical non-steroidal drugs after SLT, systemic acetazolamide (Diamox) as a temporising measure pending surgery and other immediately pre- and post-operative medications (steroids, antibiotics and non-steroidal anti-inflammatory drugs (NSAIDs)) may be used as required.

Topical lubricant drops (‘artificial tears’) will be permitted without restriction as long as preservative-free preparations are used (as per best clinical practice in this setting).

The treatment of new incident ophthalmic conditions (such as retinal vein occlusions, diabetic retinopathy or macular degeneration) may also require additional treatments that will be recorded in detail.

30.3 Appendix 5c ~ Details of Other Surgical Treatments

Some patients will progress to glaucoma drainage surgery after drop / laser treatments fail to control the IOP or prevent progressive glaucoma damage. Although it is likely that these will be comparatively few, such cases are likely to contribute disproportionately to the average cost in each treatment arm and may potentially experience more extreme values for HR-QoL.

To minimise variation between centres the first surgical intervention will be standardised. Participants will undergo mitomycin augmented trabeculectomy by whatever technique the surgeon prefers and under whatever is the patient and surgeon's preferred anaesthetic. This will be performed by a senior glaucoma-fellowship trained surgeon eligible to apply for a UK consultant surgeon post or for registration on the UK GMC Specialist Register. There will be no limit on the number of post-operative 5FU injections that may be given, but no more than two needle revisions ("needlings") of a trabeculectomy will be permitted, with or without 5FU. For the purposes of this study we shall exclude primary tube surgery as it is not yet widely accepted treatment for this patient group.

In the unlikely event of surgical failure following medical treatment failure within the 3 years follow-up then either repeat trabeculectomy or insertion of a glaucoma tube drainage implant will be permitted as per clinical need.

Other surgical interventions, such as cataract extraction or unexpected operations such as retinal detachment repair will occur. No constraints are placed on timing or techniques for these procedures other than that phacoemulsification cataract extraction must be performed and elective extra-capsular surgery will not be permitted. (Pre-existing indications for elective extra-capsular surgery would be an exclusion criterion on other grounds).

"Algorithm Holiday"

During the immediate post-operative period it would be inappropriate to use the algorithm to determine re-intervention or follow-up interval: IOPs will vary widely, acuity fluctuations will effect field tests and IOP swings may alter HRT NRR area measurements. Post-operatively there will be an 'Algorithm Holiday' of four months during which time the follow-up and treatment will be at the discretion of the clinician.

After 4 months the patient re-enters the algorithm pathway, although this may be extended at the discretion of the treating clinician if the patient is judged to be unstable due to delayed post-operative recovery (e.g. in the event of hypotony). Any extension of the "algorithm holiday" period must be

reported to Trial Manager. Short-term hypotensive topical treatment will be permitted during the algorithm holiday. Post-trabeculectomy needling will be permitted after surgery at the discretion of the treating clinician but reported to the Trial Manager.

31 Appendix 6 ~ Changing Treatment & Defining Failure

Treatment will be escalated under the following circumstances:

- ‘Strong Evidence’ of progression (irrespective of IOP)
- IOP above Target by more than a certain threshold at a single visit (irrespective of evidence for progression)
- IOP above Target by less than threshold plus “Less Strong Evidence” for progression.

If the IOP is above Target by less than threshold with no evidence for progression, then the 'Treatment Target IOP' will be re-evaluated.

See Appendix 9 for details of treatment escalation and Target re-evaluation.

31.1 Definition of ‘Failure to Meet Target’ (See flow-charts, Appendix 8)

Diurnal fluctuation and measurement error both lead to variation in measured IOP. We shall minimise the former by performing follow-up tests at a similar time of day. We shall minimise the latter through regular instrument calibration, careful observer training and robust mechanisms to demonstrate good inter-observer agreement. Kotecha *et al* have shown that inter-visit variation may nonetheless be as much as +/-4mmHg. To prevent an inappropriate escalation to more intensive treatment it is therefore important to repeat measurements that deviate only slightly from Target. Criteria for failure to meet target and to reassess Target follow those of the CGS [93], with additional steps where not specified in the CGS:

a) If an eye is ≥2mmHg but <4mmHg above Target for 2 consecutive visits and shows *possible* or *definite* progression then the treatment is intensified and the Target remains unchanged.

b) If an eye is ≥2mmHg and <4mmHg above Target for 2 consecutive visits and shows *no progression (with* a minimum of 4 fields required to confirm progression, as per EMGT [77]) then the target will be adjusted as below. If fewer than 4 VFs have been done additional visits are required to confirm stability before the Target is relaxed.

c) If an eye is ≥4^{mm}Hg from Target at any visit then the eye will be considered to *have fail*ed *to reach* Target and be advanced to the next level of treatment intensity (not on Maximum Medical Therapy (MMT, see below)), irrespective of any progression, unless the *cl*inician detects poor compliance. The Target remains unchanged. In the presence of poor compliance and the absence of progression additional measures to improve compliance before escalation of treatment will be permitted, as in clinical practice.

d) If an eye *on MMT* is ≥4mmHg from Target and shows *definite* progression then glaucoma drainage surgery will be offered to the patient.

e) If an eye *on MMT* is ≥4mmHg from Target and shows *possible* progression then the follow-up frequency will be intensified until progression is either confirmed or ruled out.

f) If an eye is ≥4mmHg from Target *and* below Maximal IOP *and* on MMT *and* shows **no** progression (with at least 4 VFs) then the Target will be adjusted with an increase in follow-up (VF) frequency. If fewer than 4 VFs have been done then additional visits will be required to confirm stability.

g) An eye that is above Maximal IOP may be offered surgery without progression (unless OHT in which case subjects with < 35mmHg will be watched and ≥ 35mmHg *offered* surgery at the discretion of the treating surgeon).

31.2 Process for Treatment-Target Reassessment

Accurately predicting a safe level of IOP for a given patient is inherently difficult before individual data on rates of nerve damage are available. International Treatment Guidelines recommend that IOP Targets are revised as further data are collected [73;85;86]. In other words, *guidelines derived from population data are refined for the individual, based on data from that individual.*

Reassessment of Target will be undertaken by the masked TRC as follows:

a) When there is failure to meet target (within 4mmHg) but no Progression: the Target will be revised to the mean of the previous 3 visits over which Progression has not occurred (a minimum of 4 fields is required to confirm progression, as per EMGT [77]).

b) When there is failure to meet target (>4mmHg) on MMT and with no Progression: the Target will be revised to the mean of the previous 3 visits over which Progression has not occurred. Any decision to *increase* the Target IOP needs ratification by the responsible consultant (usually the local PI) within two weeks and will be reported to the Chief Investigator that week.

c) When there is Progression at Target: Target will be reduced by 20% (as per Canadian Glaucoma Study (CGS) [93]) with a lower limit of 8mmHg, and treatment intensified.

31.3 Treatment escalation to glaucoma surgery

More stringent criteria are applied before undergoing surgery than administer[ing] laser or intensifying medical treatment. This reflects the greater risk to vision from surgical complications. Strong evidence of progression +/- failure to meet Target is usually

required in all but the most severe disease. However, extreme elevations of IOP may require surgery even without progression, with lower thresholds in more damaged eyes. We define '*Maximal IOP'* as that above which surgery may be offered even without progression: OHT 35mmHg; Mild glaucoma: 24mmHg; Moderate and Severe glaucoma 21mmHg. In accordance with patient-centred care the ultimate decision to operate is always a collaboration between clinician and individual patient. When an intra-ocular pressure lowering surgical intervention is indicated, cataract surgery will be permitted (in the presence of cataract, i.e. not clear lens extraction) when this is the consultant's usual practice.

32 Appendix 7 ~ Detection of Progressive Glaucoma Damage

Detection of progressive nerve damage is trigger to increasing treatment intensity. We follow NICE recommendations on follow-up intervals [1], with Humphrey Visual Field (HVF) tests and Heidelberg Retina Tomography (HRT) digital optic disc imaging at trial entry and each visit.

Progression of Glaucoma is defined as: ‘Strong evidence': GPA 'Likely progression' and/or HRT rim area >1% per year (at *P* <0.001) and ‘Less strong evidence' = GPA 'Possible progression' and/or HRT rim area >1% per year (at *P* <0.01).

32.1 Visual Field Progression

Worsening of visual field loss (VFL) will be defined as ‘Likely’ or ‘Possible’, in the absence of any identifiable retinal or neurological cause. The ‘minimum dataset’ to determine VF progression is 2 reliable baseline VF followed by 3 follow-up VF and will take 1 to 2 years from enrolment to confirm.

‘Likely VF Progression’ is 3 points or more on the HVF Glaucoma Progression Analysis (GPA) software at <0.05 probability for change on 3 consecutive occasions.

‘Possible VF Progression’ is 3 points or more on HVF Glaucoma Progression Analysis (GPA) software at <0.05 probability for change on 2 consecutive occasions.

32.2 Optic Disc Progression

Chauhan showed that sequential HRT-3 disc assessment did as well or better than ‘experts’ judging monoscopic photos [97]. While simultaneous stereoscopic disc photography has been considered a gold-standard it is not widely available and is not in regular clinical use. Worsening of disc damage will therefore be defined as a rate of neuro-retinal rim loss exceeding 1% of baseline rim area/year on a minimum of 5 repeat HRT images. This slope value is selected as it is approximately double the value of age-related RA loss [98] and gives a similar specificity to that of VF trend analyses. Images will be independently reviewed for progression by masked observers at the TRC using automated assessment algorithms.

If the treating clinician suspects disc progression in the absence of HRT deterioration and or change in GPA (e.g. due to focal NRR notching) then the HRT images will be reviewed, masked to treatment allocation and IOP data, by the TMG. The TMG will adjudicate on the clinical indication for treatment escalation in the absence of algorithm dictated escalation.

32.3 Resetting of Visual Field and Optic Disc Baselines

If treatment is escalated because of progressive glaucomatous damage as detected by either visual field or optic disc change then the 'baseline' against which future tests are compared will be reset. The measurements taken on the visit at which treatment changes are instigated will be the new baseline.

Follow-up examinations to detect continued progression will be performed at the IOP check visit (6-8 weeks post treatment change) and the next assessment. Since the patient is undergoing deterioration of HVF or HRT then the next follow-up will be at one interval sooner than would have otherwise been the case if determined by severity alone [e.g. 6 instead of 12 months, 4 instead of 6].

Escalation due to failure to reach IOP target *alone* will not result in any change to HVF or HRT baselines.

Progressive field or HRT damage at IOP above Target would also trigger a resetting of the relevant baseline.

32.4 Unreliable or Unavailable VF & HRT – dealing with missing data

32.4.1 Missing VF/HRT Data

Occasions will arise where no HRT or VF is possible e.g. patients refuse, are unwell or unreliable, or the machine is broken. However, the treatment algorithm requires a VF & HRT input.

In the case of missing HRT data the MPHSD value of 100 and a rim area of 4.0 will be used for the algorithm HRT fields and the algorithm will then ignore HRT for that visit.

If no VF is available other data will be used to determine treatment escalation (i.e. IOP wrt to Target IOP and HRT if available) as below (34. 5a)

32.4.2 Unreliable VF/HRT Data

If HRT data are unreliable (MPHSD high) or the VF data are unreliable (False Positives > 15%) the algorithm will deal with that internally and discount that data.

However repeated VF or HRT may give better data and so should be repeated upto 3 times where clinically indicated, on the same or a separate visit within a month if deemed clinically appropriate/necessary. If assessments generate consistently poor quality data the investigation may then be abandoned for future visits.

33 Appendix 8 ~ How to set the Treatment Target IOP

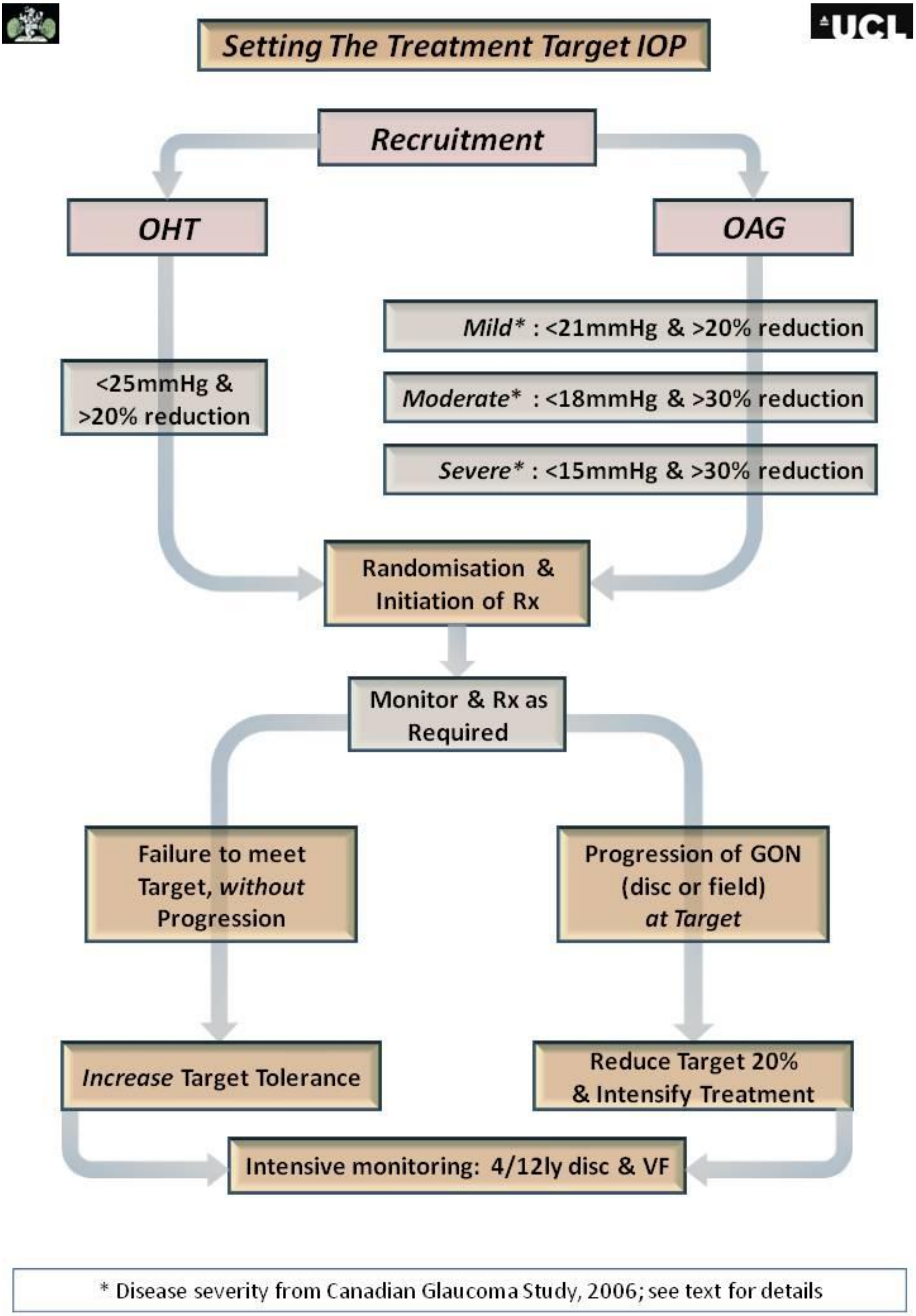

Appendix 8 (continued) ~ How to set the Treatment Target IOP

We use the Canadian Target IOP Workshop's algorithm to set the Treatment Target IOP [76]: it has clear criteria and a robust evidence base drawing on multiple larges RCTs [11;27-29;90;91]. We have added a definition of central visual field loss lacking from the original, as per Mills *et al* [92]. The Target will be either an absolute reduction to below a specified level, or a percentage reduction from baseline, whichever is lower (see flow chart above). The Target is objectively defined to avoid bias from unmasked treating ophthalmologists.

Greater reductions are required for greater disease severity as defined by Canadian Glaucoma Study criteria [93].

Surgical risks increase with low Targets. The lowest permitted Target is 8mmHg.

In accordance with NICE [1], "Glaucoma Suspects" in whom OHT is present but a definite diagnosis of GON cannot be either made or ruled out will be treated according to the OHT category.

A Target IOP algorithm will be used that is defined for each individual eye, since severity of glaucomatous optic neuropathy is important in determining the treatment target and is eye-specific.

CCT is not included in our algorithm for setting Target IOP (see main text for detailed explanation why not).

34 Appendix 9 ~ If Target is not met: when to escalate treatment and when to reassess Target for OHT (9a) and POAG (9b)

1. **What defines progression?** *Either* HRT *or* VF *or both.* If either test triggers an early review for increased frequency of testing then *both* HRT and VF will need to be done on that visit. Every time a HVF or HRT is done the algorithm must be run.
2. **'Progression' for OHT:** is also 'conversion to OAG' and may happen at any stage of the treatment escalation and will be defined as confirmed change in HRT (as per standard trial criteria) or development of *any* new visual field defect.
3. **How many tests are needed?** If there are fewer than 4 fields or HRTs available to define progression then treatment should continue until more fields / HRT are available, unless absolute IOP criteria are met independent of requirement to define progression (e.g. >maximal IOP). Additional VF tests will be permissible at any visit if necessary to confirm possible progression, as per usual clinical practice.
4. **Possible Progression at or within 2mmHg of Target** will increase the follow-up frequency by reducing the interval to review (from 12 to 6 and 6 to 4 months).
5. **Visual Fields and HRT reliability**
    a. **If Visual Fields are consistently unreliable** (as per Zeiss inbuilt GPA software criteria, i.e. false positive responses) then we will use IOP criteria and/or HRT progression. In the *absence* of HRT progression:
        i. If IOP ≤ 2mmHg above Target then continue
        ii. If IOP ≥ 2mmHg above Target not on MMT then increase treatment
        iii. If IOP ≥ 2mmHg and < 4mmHg above Target on MMT then continue and decrease review frequency
        iv. If IOP ≥ 4mmHg above Target on MMT then offer surgery (if OAG)
        v. If IOP ≥ 4mmHg above Target on MMT *and* IOP < 35mmHg then continue and decrease review frequency (if OHT)
        vi. If IOP ≥ 4mmHg above Target on MMT *and* IOP ≥ 35mmHg then consider offering surgery (if OHT)
    b. **If HRT scans are consistently unreliable** (as per accepted criteria, i.e. mean pixel height SD >40; due e.g. to cataract) then:
        i. If visual fields are available they will be used to define progression
        ii. If neither are available then IOP alone will guide treatment escalation (as per part 5.a above)

6. **Changing Severity Category:** subjects will *remain* in the category in which they started the trial for purposes of setting **Target IOP** even if they deteriorate and become eligible for a more severe category. This is necessary to avoid 'flip-flopping' of borderline cases between severity classes with attendant Target IOP changes due to natural variation in the Mean Deviation of visual field tests due to long-term fluctuation (ie 'noise' rather than trend).

This will not deny any subject more intensive treatment because the Target Setting and Escalation Algorithms will themselves lead to the required intensification of therapy.

Follow-up interval will however use the *current* severity category so that deteriorated patients are seen more often even after stabilising.

Maximal IOP will, likewise, be determined by the *current* severity category.

Thus a deteriorating visual field can trigger an escalation of treatment by *both* reaching GPA threshold for deterioration *and* by changing the maximal IOP due to a severity category change.

7. **New or worsening co-pathology, including cataract:** any escalation based upon progression will require the exclusion of new or worsening co-pathology, including cataract, as a possible cause by the responsible clinician.

34.1 Appendix 9a ~ If Target is not met (OHT) : when to escalate treatment and when to reassess Target

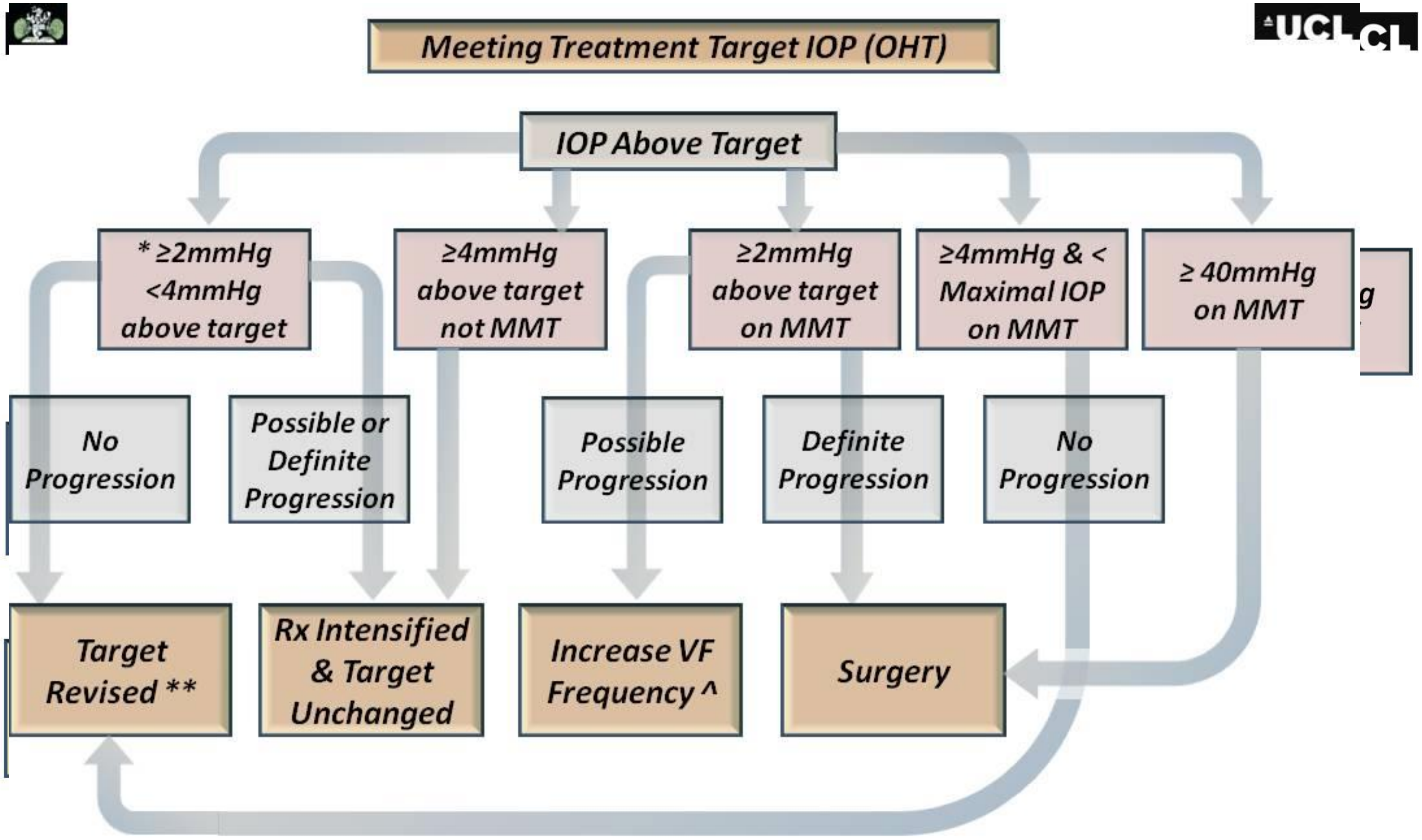

*On 2 consecutive visits; ** as per revision protocol; ^ until progression confirmed/refuted; all VF progression requires 4 VF
'Maximal IOP' = IOP above which surgery offered even without progression (see text) = 40mmHg for OHT

'Maximal IOP' = IOP above which surgery offered even without progression (see text) = 40mmHg for OHT

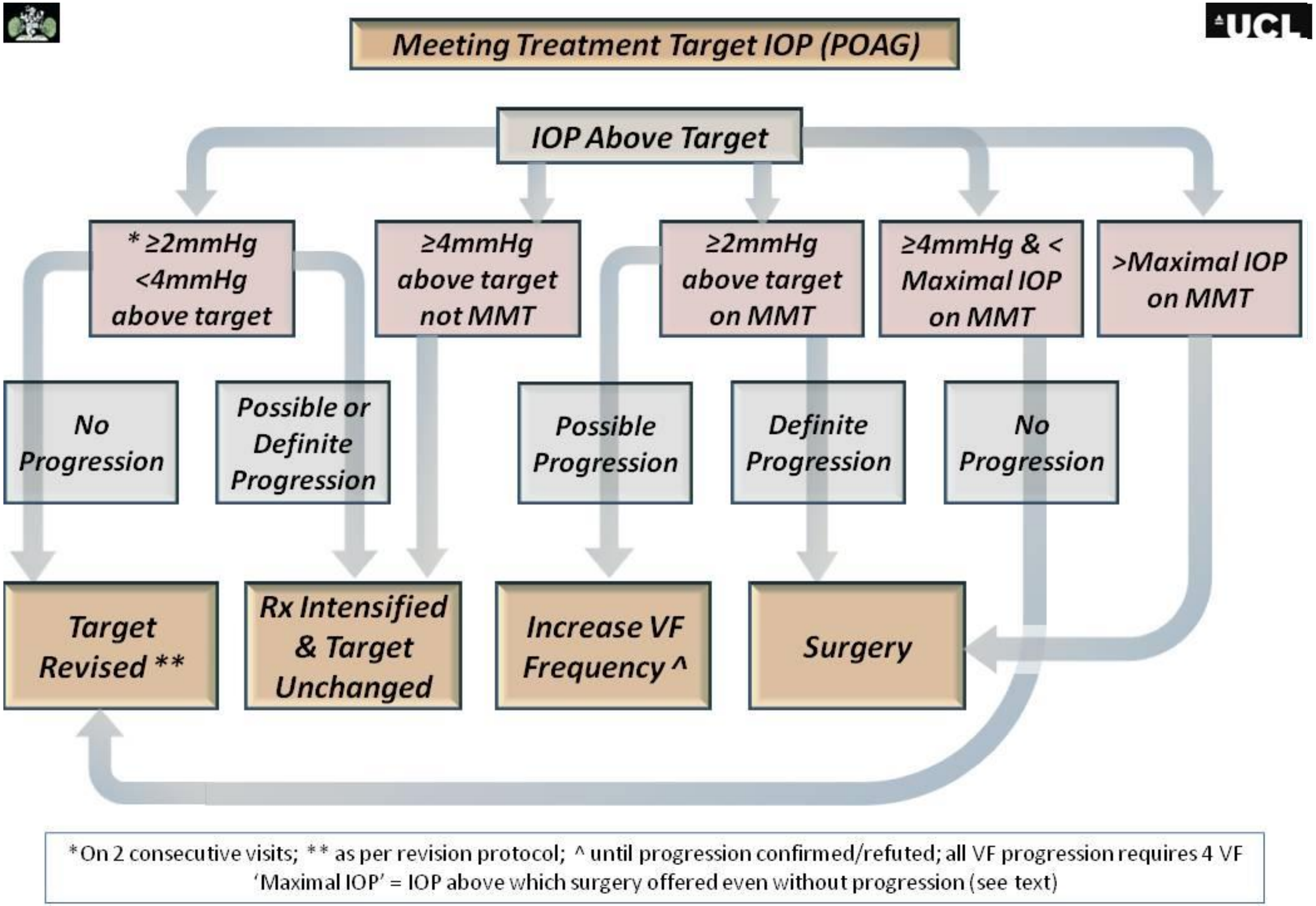

34.2 Appendix 9b ~ If Target is not met (POAG): *when* to escalate treatment and when to reassess Target

35 Appendix 10 ~ *How* to Escalate Treatment

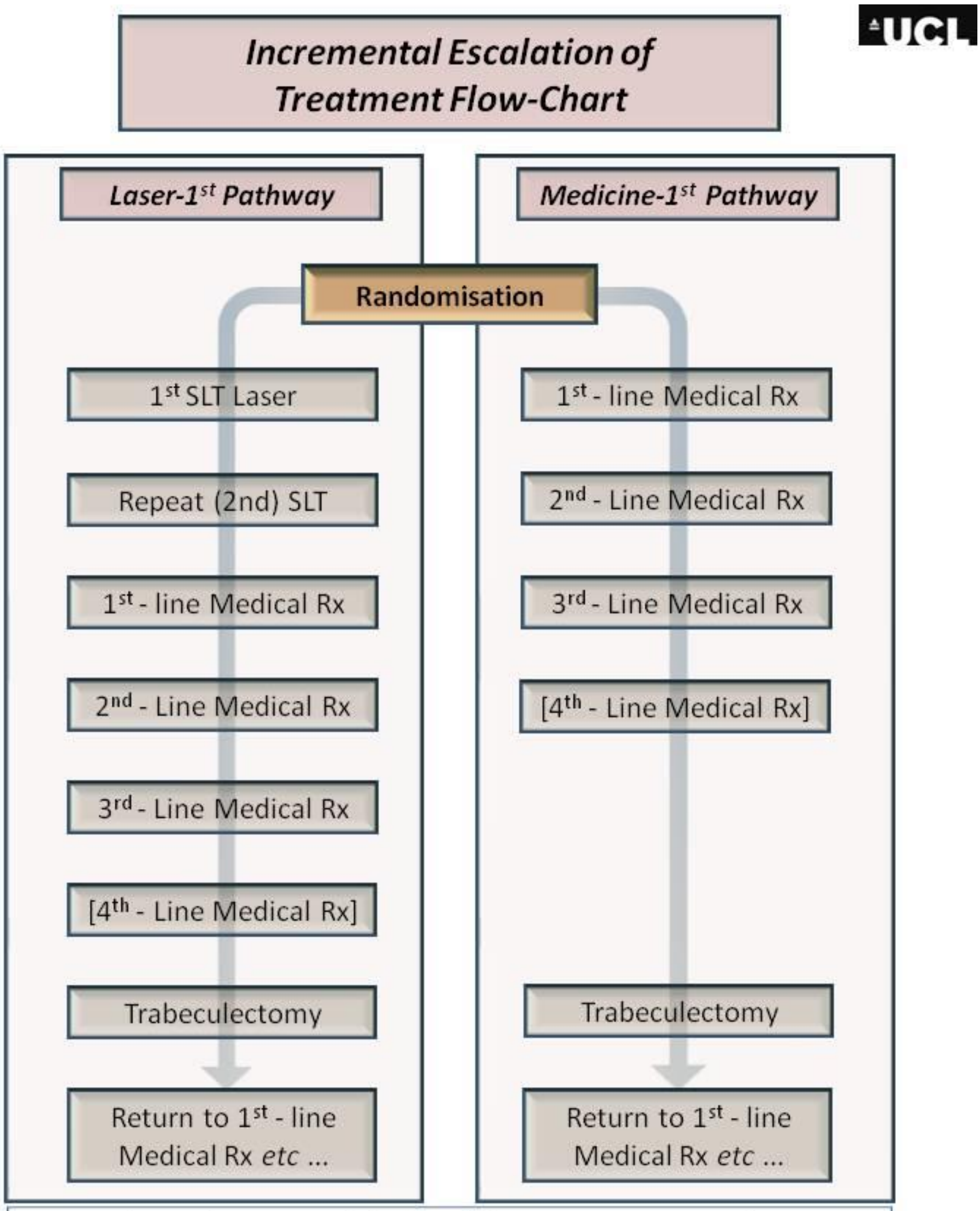

- Progression to the next step is indicated if Treatment Target IOP is not reached and/or progression of GON occurs.
- Target IOP may be redefined in the light of additional data on VF stability.
- Treatment may be switched or added at each stage
- Drugs suitable for 1st, 2nd line etc. and surgical parameters defined in text
- If SLT in the 1st eye is complicated, laser may be contraindicated in the 2nd eye. The patient remains in the laser pathway but is classified as a treatment failure for laser and enters the medication route.
- Any treatment *reductions* will be made clinically and *not* by algorithm

36 Appendix 11 ~ Questionnaires

36.1 Delivery and Follow-up

The Baseline Questionnaires will be self-administered, in a private room, at the time of enrolment after informed consent has been given but *before* randomisation. Participants are required to have sufficient English that translation is not required - practical assistance with the lay-out and completion of the form only will be permitted.

Subsequent questionnaires will be sent out by post for self-completion at 6 monthly intervals with up to two written reminders and then one telephone follow-up in the case of non-response. In the event of telephone follow-up the primary outcome measure will be collected first.

36.2 Questionnaire Content

The content of the questionnaires in this trial is determined by the use of a number of validated, widely accepted existing questionnaires, namely: EQ-5D, GUI, GSS, GQL-15.

The additional questions to be included will be those of the modified CSRI and finally a question concerning about concordance / compliance at exit from the study.

Final questionnaire layout and clarity will be reviewed by the PPI group to ensure ease of completion.

36.2.1 EQ-5D [74]

Participants are asked: “Under each heading, please tick the ONE box that best describes your health TODAY”, and then to complete an analogue score.

MOBILITY
- I have no problems in walking about
- I have slight problems in walking about
- I have moderate problems in walking about
- I have severe problems in walking about
- I am unable to walk about

SELF-CARE
- I have no problems washing or dressing myself
- I have slight problems washing or dressing myself
- I have moderate problems washing or dressing myself
- I have severe problems washing or dressing myself
- I am unable to wash or dress myself

USUAL ACTIVITIES *(e.g. work, study, housework, family or leisure activities)*
- I have no problems doing my usual activities
- I have slight problems doing my usual activities
- I have moderate problems doing my usual activities
- I have severe problems doing my usual activities
- I am unable to do my usual activities

PAIN / DISCOMFORT

I have no pain or discomfort
I have slight pain or discomfort
I have moderate pain or discomfort
I have severe pain or discomfort
I have extreme pain or discomfort

ANXIETY / DEPRESSION

I am not anxious or depressed
I am slightly anxious or depressed
I am moderately anxious or depressed
I am severely anxious or depressed
I am extremely anxious or depressed"

36.2.1.1 EQ5D Analogue Scale

- We would like to know how good or bad your health is TODAY.
- This scale is numbered from 0 to 100.
- 100 means the best health you can imagine.
  0 means the worst health you can imagine.
- Mark an X on the scale to indicate how your health is TODAY.
- Now, please write the number you marked on the scale in the box below.

YOUR HEALTH TODAY = ☐

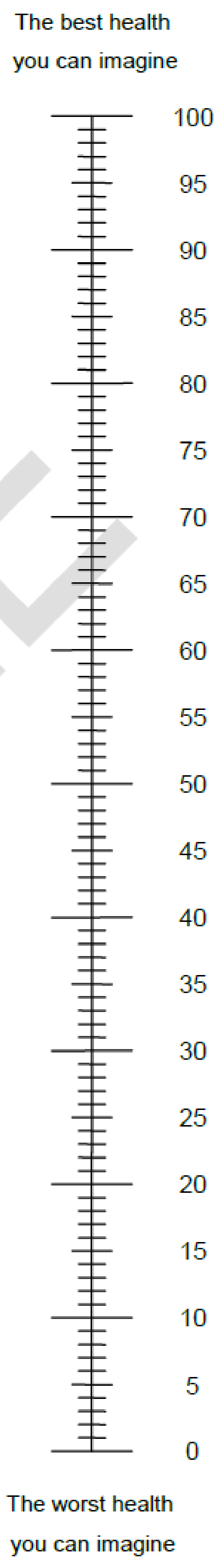

SAMPLE

36.2.2 Glaucoma Utility Index [83] (GUI)

Five questions (each testing different 'domains') ask about difficulties with certain activities and are scored by the subject as *None*; *Some*; *Quite a lot* or *Severe.*

"Tick one box, for each of the categories 1-6, which best describes any difficulties you have had in the last month with your eyes or vision, wearing your usual glasses.

You may need to refer back to the guide "Guide to aspects of quality life that may be affected in glaucoma and associated levels of difficulty" to help you answer these questions."

1. **Central and Near Vision**

   For example difficulties with reading, writing, watching TV, reading dials on clocks?

2. **Lighting and glare**
   For example difficulties with adjusting from light to dark and vice-versa, bright lights may dazzle, difficulties seeing in dim light?

3. **Mobility**
   For example difficulties with crossing roads, driving, negotiating steps, kerbs, busy pavements etc?

4. **Activities of daily living**
   For example difficulties with household or DIY tasks, pouring liquids into containers, putting crockery into cupboards, shaving etc?

5. **Eye discomfort**
   For example difficulties with gritty, sore, tired eyes?

6. **Other effects**
   For example fatigue, shortness of breath, dry mouth, bitter taste etc?

36.2.3 Glaucoma Symptom Scale [84] (GSS)

Participants are asked: "Have you experienced any of the following problems in the last 4 weeks?" ("Please respond for both the left and right eye.")

1. Burning, Smarting, Stinging
2. Tearing
3. Dryness
4. Itching
5. Soreness, Tiredness
6. Blurry/Dim Vision
7. Feeling of Something in Your Eye
8. Hard to See in Daylight

9. Hard to See in Dark Places
10. Halos Around Lights

Right / Left Eye: Yes / No   If Yes, how bothersome has it been?

- Very
- Somewhat
- A Little
- Not at All

36.2.4 Glaucoma Quality of Life - 15 [8] (GQL-15)

All the items in the GQL-15 are scored on a five-category difficulty scale, as follows:

1 no difficulty

2 a little bit of difficulty

3 some difficult

4 quite a lot of difficulty

5 severe difficult

0 do not perform for non-visual reasons

1. Reading newspapers
2. Walking after dark
3. Seeing at night
4. Walking on uneven ground
5. Adjusting to bright lights
6. Adjusting to dim lights
7. Going from light to dark room or vice versa
8. Tripping over objects
9. Seeing objects coming from the side
10. Crossing the road
11. Walking on steps/stairs
12. Bumping into objects
13. Judging distance of foot to step/curb
14. Finding dropped objects
15. Recognizing faces

36.2.5 Client Services Receipt Inventory [99;100] (CSRI)

Please enter the number of times you have been in contact with each health care service in the last **6 months**.

- **Eye related services**
  Number of times in last 6 months:
  Optician / Optometrist
  Hospital Eye Clinic
- **Specialist/Acute hospital services**
  Number of admissions in last 6 months & Total number of nights in hospital:
  - Overnight inpatient stay
    Planned inpatient stay
    Emergency inpatient stay
    Intensive care / High dependency unit
  - Outpatient appointment
  - Day patient procedure/test
  - Accident and Emergency visit
    And admitted to hospital
    Not admitted to hospital
- **General Practitioner (GP) and community services**
  Number of times in last 6 months & eye-related vs not-eye-related
  - GP Contacts
    In the GP practice
    By telephone
    At a home visit
  - GP Practice Nurse
  - Social Worker
  - Other home care worker (e.g. district nurse)
  - Other Community services (Please give details)
- **Medication (tablets, pills, capsules and all medicines)**
  Drug Name (either trade name or medical name)
  Duration of use (number of days, or put continuous is on it for the whole time)
  Daily Dose (Dose and number each day)
- **Private Medicine and Health Insurance**
  Did you access private health care in the last 6 months? Yes / No
  (either through health insurance or independently)
  If yes, list below the private health care services you used in the last 6 months

36.2.6 Concordance / Compliance

A pair of questions will be asked about drop usage and compliance, that have been validated in a large study of compliance with drop therapy in glaucoma (personal communication, Prof David Friedman, Johns Hopkins) and shown to be predictive of non-compliance:

1. "Over the past month, what percentage of your drops do you think you took correctly?"
2. On a Likert scale (of: strongly disagree, somewhat disagree, neither agree nor disagree, somewhat agree, and strongly agree) participants will be asked to respond to the following statement): "I'm the sort of person who follows doctors' orders exactly."

37 Appendix 12 Project Gantt Chart

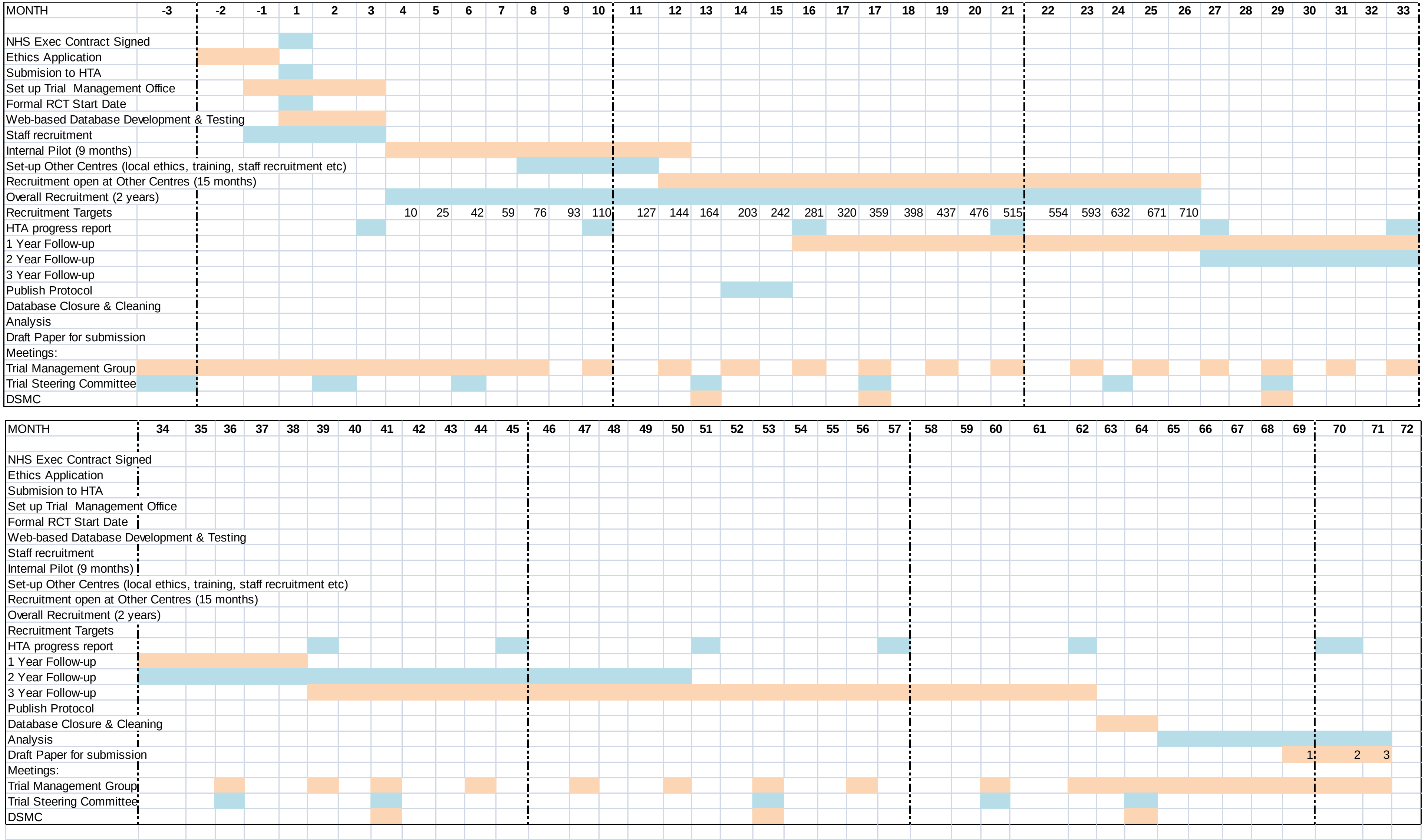

All milestones to start at beginning of first month listed and be complete by end of month listed.

TSC dates have been chosen to coincide with (and take place at) major academic meetings where possible to minimise travel costs (May ARVO & Dec UKEGS)

38 Appendix 13 "Guidelines on questions to ask in clinic for clinical management"

Questions for every visit

1 Have you had any problems with your eyes since we saw you last?
2 Are you having any other problems (addition)

Questions for patients on medication

3 Have you had any difficulties in taking your eye-drops? (if being used)
4 Have you had any shortness of breath, wheeze or used an inhaler since your last visit? (all patients, including those on beta-blockers)
5 Have you had any problems from a dry mouth since we saw you?
6 Have you developed or had any worsening of angina? (all patients, including those not on alpha-agonists)

7 Have you been excessively tired or sleepy?

39 Appendix 14 " Video Script – presented by Gus Gazzard

This video has been designed to inform you about a research study that is ongoing at Moorfieds Eye Hospital. The video will introduce you to Glaucoma and Ocular Hypertension, the various treatment options that are available and will eventually invite you to take part in a research study investigating the quality of your life after treatment.

We would be grateful if you could spend 5-10 minutes watching this video.

Glaucoma is a disease of the optic nerve, which connects the eye to the brain. Glaucoma slowly progresses over a period of years; at the early stages people may not notice anything abnormal, but in advanced disease people may notice loss of vision. At the early stages glaucoma can be treated with eye drops or a laser treatment, which aim to control the condition and minimise future damage. Early diagnosis is important because any damage cannot be reversed. If Glaucoma is left untreated it can cause visual impairment. Glaucoma may be caused by raised eye pressure, but sometimes Glaucoma develops despite a normal pressure inside the eyes, due to a poorer blood supply or a weaker optic nerve.

Ocular hypertension is a condition where the pressure of the eyes is above normal limits, without, however, this causing any damage to the optic nerve. Some people have higher

pressures than others. It has been shown that ocular hypertension puts people at a higher risk for developing glaucoma. Some people with ocular hypertension may, however, never develop Glaucoma.

The pressure of your eyes is important, as your eyes function properly under a certain amount of pressure. If this pressure increases the optic nerve can be damaged. The amount of damage may depend on how high the pressure is, how long it lasts, and whether there is a poor blood supply or other weaknesses of the optic nerve. By lowering the pressure damage is slowed down.

At the moment in the NHS nearly all patients who have Glaucoma or Ocular Hypertension are treated by eye drops. Once started eye drops usually have to be continued for life. Not all patients like using drops daily, however, and these patients might be suitable to a gentle laser therapy called Selective Laser Trabeculoplasty. This laser treatment is not experimental; it is used commonly in the UK and for a number of years in the United States and its efficacy has been proven. At the moment the laser treatment is not offered as a first line treatment in the NHS.

This study is designed to investigate the quality of life in patients treated first time either with drops or with laser and is being funded by the National Institute of Health Research. The study will use questionnaires that the patients will have to fill in every 6 months. A secondary aim is to assess the cost of these treatments to the NHS.

Because we don't know which treatment will prove preferable for the patients' quality of life or which treatment is more cost effective for the NHS, patients in this study will be assigned randomly to one of the two treatments and the two groups will then be compared. This type of study is called a randomised controlled trial, where half the patients will be randomly assigned by a computer to drops and half will be assigned to laser treatment. If we assigned you to a group we might show preference to a specific treatment and if you were to choose, you might be biased towards one treatment.

Participation in this study is entirely voluntary. If you decide not to take part this will have no effect on the quality of care you receive at Moorfields Eye Hospital. If you decide to take part you will only be asked to attend the clinic one extra time compared to the usual clinic, but we can reimburse your travel expenses. The reason we will ask you to come one extra time is to give you time to think about the study and allow you to ask us any questions you might have.

If you decide to take part in this study you will be monitored by our specially trained Optometrist and you will do the exact same tests you would do in a normalclinic. This is because the study is a real life study, investigating your quality of life. This study will last in total 5 years, but each patient will be monitored for 3 years after the treatment is started. For this period of time you will be asked to attend the clinic between 5 and 7 times. If you do not take part in the study you will be asked to attend the clinic 6 to 7 times. After the end of the study you will continued to be monitored by the glaucoma clinics at Moorfields Eye Hospital as usual.

If you decide to take part you will be asked to fill in a questionnaire about your health and about your eyes. You will then be assigned to having drops or laser and you will be seen a few weeks later to assess if the treatment is working, just as we would if you were not in the study. Twice a year we will send you a questionnaire by post, which after filling in you can return to us in a pre-paid envelope that we will also send.

As every treatment, the treatments in this study might have some side effects. Eye drops are used for approximately 30 years and can have mild or more severe side effects. Drops can cause mild discomfort or redness of the eyes, which usually settle soon, but in some cases they might make asthma worse. We will make sure we ask you all the necessary questions about your health before prescribing any drops. Some types of eye drops should not be given to pregnant women. Any woman who is pregnant or who is planning to become pregnant should therefore not take part in this study. If a woman taking part in the study becomes pregnant she should let the research team know immediately. If the drops are not lowering your eye pressure enough you will be offered different or additional eye drops.

The laser has been used successfully for 10-20 years. It is not a surgery and it is safe, easy and painless to deliver. In some people it might cause a small discomfort for a few days, which can be treated with anti-inflammatory eye drops. Very rarely the laser might cause the eyes to be blurry for a few weeks or it may cause the pressure of the eyes to increase. If this happens we will give you drops to use for a few days. Laser treatment is effective in 80-90% of patients and its effect might wear off after a few years. If this happens the laser can be repeated once more. If for some reason we still need to reduce your eye pressure we will give you drops.

This study has no direct risks or benefits to you, as it is designed to mimic normal clinical practise. This means that you will be doing exactly what you would be doing in a normal glaucoma clinic and nothing additional to that. If during the study you decide you don't want to take part any more you can withdraw without providing a reason and without this affecting your care at Moorfields Eye Hospital. If you choose to withdraw you will then be monitored by a normal clinic. aIf you decide to take part you will be assigned an ID and all the information will be kept strictly confidential.

Two of the patients who have already taken part in our study have kindly agreed to explain the reasons they decided to participate and their experience so far.

Patient experiences – presented by patients who are currently enrolled in the study

Having explained why Moorfields Eye Hospital is conducting this study I hope you will be willing to consider taking part. My colleagues will now give you an information sheet about this study and will be able to answer any further questions you might have in relation to the study. Thank you very much for your time.
