# Supplementary material for: Selective Laser Trabeculoplasty After Medical Treatment for Glaucoma or Ocular Hypertension
Source: JAMA Ophthalmol. 2025 Feb 20;143(4):295–302. doi: 10.1001/jamaophthalmol.2024.6492 (PMC11843460; doi:10.1001/jamaophthalmol.2024.6492)
Supplement: Supplement 3. — eFigure 1. Histogram of Timing of the Secondary SLT Treatments in eyes That Had Originally Received Primary Medical Treatment (IOP-Lowering Eye-Drops) eFigure 2. Medication-Load Profile at 6 Years for the Eyes at Target That Were Switched to SLT Following Primary Medical Treatment Compared to the Eyes That Continued Medical Treatment Alone eTable 1. Timing and Disease Severity at the Time of Secondary SLT for Eyes That Switched to SLT and Eyes That Escalated to SLT After a Period of at Least 3 Years in Primary Medical Treatment eTable 2. Characteristics for the Participants Remaining on IOP Lowering Eye-Drops or Receiving Secondary SLT After a Period of at Least 3 Years and Those Receiving Primary SLT eTable 3. Mean and Percentage IOP Reduction (From Baseline) for the 69 Eyes Switched to SLT After Primary Medical Treatment, Needing No Medication or Surgery at the End of the Trial eTable 4. Number of Eyes Reaching Target IOP at the End of the Trial and Need for Subsequent Trabeculectomy in Eyes Escalating to SLT, by Treatment Intensity Before Escalation eTable 5. Absolute and Percentage IOP Reduction Before and After SLT for Eyes That Escalated to SLT [file jamaophthalmol-e246492-s003.pdf]

Supplemental Online Content

Konstantakopoulou E, Gazzard G, Garway-Heath D, et al; the LiGHT Trial Study Group. Selective laser trabeculoplasty after medical treatment for glaucoma or ocular hypertension. *JAMA Ophthalmol*. Published online February 20, 2025. doi:10.1001/jamaophthalmol.2024.6492

eFigure 1. Histogram of Timing of the Secondary SLT Treatments in eyes That Had Originally Received Primary Medical Treatment (IOP-Lowering Eye-Drops)

eFigure 2. Medication-Load Profile at 6 Years for the Eyes at Target That Were Switched to SLT Following Primary Medical Treatment Compared to the Eyes That Continued Medical Treatment Alone

eTable 1. Timing and Disease Severity at the Time of Secondary SLT for Eyes That Switched to SLT and Eyes That Escalated to SLT After a Period of at Least 3 Years in Primary Medical Treatment

eTable 2. Characteristics for the Participants Remaining on IOP Lowering Eye-Drops or Receiving Secondary SLT After a Period of at Least 3 Years and Those Receiving Primary SLT

eTable 3. Mean and Percentage IOP Reduction (From Baseline) for the 69 Eyes Switched to SLT After Primary Medical Treatment, Needing No Medication or Surgery at the End of the Trial

eTable 4. Number of Eyes Reaching Target IOP at the End of the Trial and Need for Subsequent Trabeculectomy in Eyes Escalating to SLT, by Treatment Intensity Before Escalation

eTable 5. Absolute and Percentage IOP Reduction Before and After SLT for Eyes That Escalated to SLT

This supplemental material has been provided by the authors to give readers additional information about their work.

© 2025 Konstantakopoulou E et al. *JAMA Ophthalmology*.

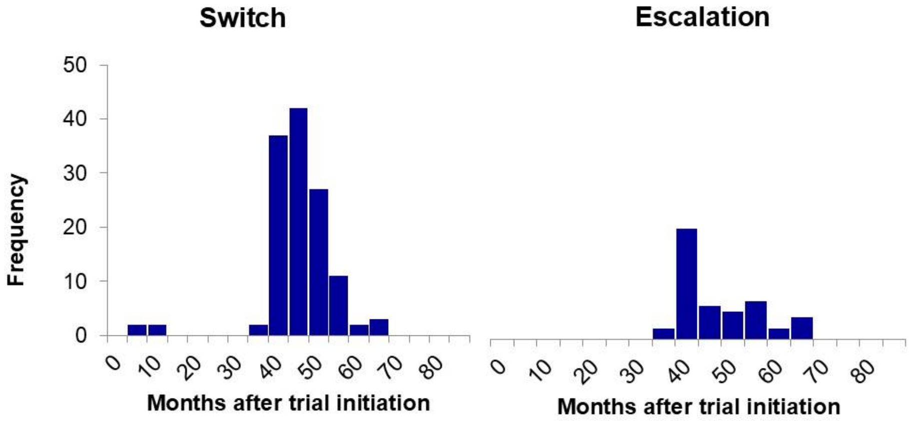

e-Figure 1: Histogram of timing of the secondary SLT treatments in eyes that had originally received primary medical treatment (IOP-lowering eye-drops). Switch – median (IQR) months from baseline: 42.7 months (38.6 to 46.4). Escalation – median (IQR) months from baseline: 42.4 months (37.2 to 50.5). One patient (2 eyes) decided to receive SLT after randomization to primary medical treatment (month 0), as reported previously[1].

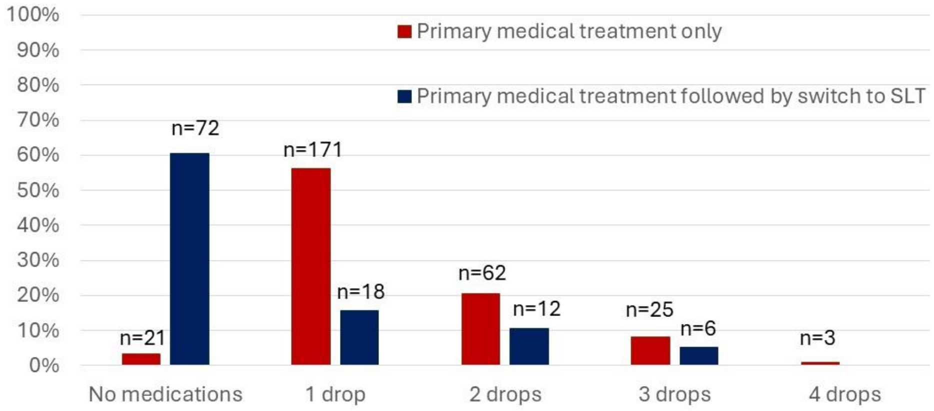

e-Figure 2 Medication-load profile at 6 years for the eyes at target that were switched to SLT following primary medical treatment compared to the eyes that remained on medical treatment alone. Eyes that needed surgery are excluded.

© 2025 Konstantakopoulou E et al. *JAMA Ophthalmology*.

	Primary medical treatment	
	**Switch to SLT**	**Escalations to SLT**
**Total** (eyes)	128	48
**Total** (participants)	83	42
**Diagnosis at time of SLT switch/escalation**		
OHT	39 (30.5%)	8 (16.7%)
Mild OAG	67 (52.3%)	20 (41.7%)
Moderate OAG	12 (9.4%)	9 (18.8%)
Severe OAG	10 (7.8%)	11 (22.9%)
Timing of SLT (months from baseline) (median, IQR)	42.7 (38.6 to 46.4)	42.4 (37.2 to 50.5).

e-Table 1: Timing and disease severity at the time of secondary SLT for eyes that *switched* to SLT and eyes that *escalated* to SLT after a period of at least 3 years in primary medical treatment. *2 eyes of 1 participant had SLT at baseline and 2 eyes of 1 participant had SLT in the first 3 years; these are counted as treatment switches; 13 participants escalated in one eye and switched in the other eye.

	**Primary medical treatment** (320 participants)		**Primary SLT** (313 participants)
	**Drops only (n=208)**	**Drops & secondary SLT(n=112)**	
Age - Mean(SD)	63.4 (11.7)	62.7 (11.0)	63.1 (12.0)
Sex			
Male	112 (54.8%)	58 (51.8%)	178 (56.8%)
Female	96 (46.2%)	54 (48.2%)	135 (43.1%)
Ethnic Origin			
Asian	16 (7.7%)	10 (8.9%)	23 (7.3%)
Black	36 (17.3%)	21 (18.8%)	67 (21.4%)
White	152 (73.1%)	79 (70.5%)	211 (67.4%)
Other	4 (1.9%)	2 (1.8%)	12 (3.8%)
Diagnosis			
OHT	44 (21.2%)	25 (22.3%)	71 (22.7%)
OAG	164 (78.8%)	87 (77.7%)	242 (77.3%)
Family history of glaucoma of 1st degree relative			
Yes	59 (28.4%)	35 (31.3%)	100 (31.9%)
No	149 (71.6%)	77 (68.7%)	212* (67.7%)

e-Table 2: Characteristics for the participants remaining on IOP lowering eye-drops or receiving secondary SLT after a period of at least 3 years and those receiving primary SLT. Numbers and percentages are shown, unless otherwise stated. * data for 1 participant missing

© 2025 Konstantakopoulou E et al. *JAMA Ophthalmology*.

Switch to SLT	Mean (SD)	Median (IQR)
IOP reduction	8.7 (4.6)	8 (6 to 10)
% IOP reduction	33.9 (11.3)	32.1 (25.9)
Time to SLT from baseline (years)	3.6 (0.7)	3.6 (3.2 to 3.9)

e-Table 3 Mean and % IOP reduction (from baseline) for the 69 eyes switched to SLT after primary medical treatment, needing no medication or surgery at the end of the trial (72-months). 61 eyes were on 1 medication before SLT, 6 eyes were on 2 medications and 1 eye was on 3 medications before switching to SLT.

	N of eyes	N (%) reaching target*	N (%) needing trabeculectomy
1 drug pre-SLT	19	11 (57.9%)	4 (21.05%)
2 drugs pre SLT	21	14 (66.6%)	5 (23.8%)
3+ drugs pre SLT	8	5 (62.5%)^	0

e-Table 4 Number of eyes reaching Target IOP at the end of the study and need for subsequent trabeculectomy during the course of the study, in eyes escalating to SLT, by treatment intensity before escalation. *6 eyes do not have 72-month data; ^3 eyes did not reach target at 72-month: one eye had additional SLT, one eye had additional medication and one eye had a medication change.

	N	IOP reduction Mean (SD)	% IOP reduction Mean (SD)
**All eyes**	47	4.6 (4.7)	21.8 (23.0)
**1 drug pre-SLT**	18*	4.8 (4.4)	23.8 (23.4)
**2 drugs pre-SLT**	21	5.0 (4.4)	22.8 (20.3)
**3+ drugs pre-SLT**	8	2.6 (4.7)	15.0 (25.1)

e-Table 5 Absolute and % IOP reduction before and after SLT for eyes that escalated to SLT. * 1 eye is missing post-SLT IOP data.

© 2025 Konstantakopoulou E et al. *JAMA Ophthalmology*.
