# Supplementary material for: Selective Laser Trabeculoplasty After Medical Treatment for Glaucoma or Ocular Hypertension
Source: JAMA Ophthalmol. 2025 Feb 20;143(4):295–302. doi: 10.1001/jamaophthalmol.2024.6492 (PMC11843460; doi:10.1001/jamaophthalmol.2024.6492)
Supplement: Supplement 4. — Data Sharing Statement [file jamaophthalmol-e246492-s004.pdf]

Data Sharing Statement

Konstantakopoulou. Selective Laser Trabeculoplasty After Medical Treatment for Glaucoma or Ocular Hypertension. *JAMA Ophthalmol*. Published February 20, 2025.
doi:10.1001/jamaophthalmol.2024.6492

Data
Additional Information: This study is registered at controlled-trials.com (ISRCTN32038223).
Data available: No

Additional Information
Explanation for why data not available: Data might be available upon request.
